# Highly modified and immunoactive N-glycans of the canine heartworm

**DOI:** 10.1038/s41467-018-07948-7

**Published:** 2019-01-08

**Authors:** Francesca Martini, Barbara Eckmair, Saša Štefanić, Chunsheng Jin, Monika Garg, Shi Yan, Carmen Jiménez-Castells, Alba Hykollari, Christine Neupert, Luigi Venco, Daniel Varón Silva, Iain B. H. Wilson, Katharina Paschinger

**Affiliations:** 1 Institut für Microbiologie, ETH Zürich, Zürich, 8093 Switzerland; 2 Department für Chemie, Universität für Bodenkultur, Muthgasse 18, 1190 Wien, Austria; 3 Institute of Parasitology, Universität Zürich, Winterthurerstraße 266a, 8057 Zürich, Switzerland; 4 Institutionen för Biomedicin, Göteborgs Universitet, 405 30 Göteborg, Sweden; 5 Max-Planck-Institut für Kolloid- und Grenzflächenforschung, Biomolekulare Systeme, 14424 Potsdam, Germany; 6 Institut für Parasitologie, Veterinärmedizinische Universität, 1210 Wien, Austria; 7 Malcisbo AG, Wagistrasse 27a, 8952 Schlieren, Switzerland; 8 Clinica Veterinaria Lago Maggiore, Arona, 28040 Italy

## Abstract

The canine heartworm (*Dirofilaria immitis*) is a mosquito-borne parasitic nematode whose range is extending due to climate change. In a four-dimensional analysis involving HPLC, MALDI-TOF–MS and MS/MS in combination with chemical and enzymatic digestions, we here reveal an N-glycome of unprecedented complexity. We detect N-glycans of up to 7000 Da, which contain long fucosylated HexNAc-based repeats, as well as glucuronylated structures. While some modifications including LacdiNAc, chitobiose, α1,3-fucose and phosphorylcholine are familiar, anionic N-glycans have previously not been reported in nematodes. Glycan array data show that the neutral glycans are preferentially recognised by IgM in dog sera or by mannose binding lectin when antennal fucose and phosphorylcholine residues are removed; this pattern of reactivity is reversed for mammalian C-reactive protein, which can in turn be bound by the complement component C1q. Thereby, the N-glycans of *D. immitis* contain features which may either mediate immunomodulation of the host or confer the ability to avoid immune surveillance.

## Abstract (web summary)

The glycome of parasites can have immunomodulatory properties or help to avoid immune surveillance, but details are unknown. Here, Martini et al. characterize the N-glycome of the canine heartworm, reveal an unprecedented complexity, particularly in anionic N-glycans, and determine recognition by components of the immune system.

## Introduction

Many species of nematodes are parasites of mammals and have specific tissue and host tropisms. The dog heartworm, *Dirofilaria immitis*, has specialised in infestation of pulmonary artery and heart; it infects canine and feline species causing severe disease, but has also been reported in humans especially in the Americas and Japan^1,2^. *D. immitis* is spread via mosquitoes and thus has a lifecycle not very dissimilar from other insect-borne filarial worms including *Onchocerca volvulus* and *Wuchereria bancrofti*, which though reside, respectively, in the human subcutaneous and lymphatic tissues. Owing to the expansion of the geographical range of the relevant insect vectors, insect-borne nematode diseases are spreading across Europe and so *D. immitis* is an example of a zoonotic threat emerging due to climate change^3,4^. On the other hand, as for many nematodes, treatment of dirofilariasis is based on macrocyclic lactones such as ivermectin, but cases of resistance have now been reported in parts of the United States^5^; thus, other treatment or prevention strategies, including vaccination, have been considered^6–8^.

There is some evidence that glycoproteins of the heartworm are immunogenic as judged by studies on preparation of monoclonal and polyclonal antibodies^9–11^, although these antigens may not be necessarily accessible on the surface of adult *D. immitis*. Furthermore, as some nematode glycoconjugates are immunomodulatory, especially those modified by phosphorylcholine^12^, the analysis of the protein-linked glycans of nematodes is of interest not just for inter-species comparison, but also to understand their potential biological activity. In the case of *D. immitis*, there is fragmentary information regarding its glycomic capacity (i.e. the range of possible glycan modifications) with data based on radiolabelling and lectin affinity indicating the potential presence of core and antennal fucose on tri- and tetra-antennary N-glycans, some of which are capped with *N-*acetyl-galactosamine^13^, as well as of oligomannosidic structures^14^.

Considering the potential biological relevance of the glycans of this species, we embarked on a glycomic analysis using mass spectrometric-based methods of male and female adult heartworms. Not only were the expected fucosylated and non-fucosylated forms of LacdiNAc (GalNAcβ1,4GlcNAc) motifs found, but also high molecular weight N-glycans with long *N-*acetylhexosamine (HexNAc)-based repeat units with modifications by fucose (Fuc), phosphorylcholine (PC) or glucuronic acid (GlcA). Particularly, the presence of poly-HexNAc-based antennae capped with glucuronic acid is unprecedented in any species, especially as anionic N-glycans have previously not been found in nematodes. Furthermore, for the first time, we test the ability of selected lectins, immunoglobulins, a pentraxin (C-reactive protein) and the complement component C1q to directly or indirectly bind natural parasitic nematode glycans in an array format, which paves the way for structure-informed functional studies.

## Results

### General strategy for Dirofilaria N-glycomics

N-glycans were released from proteins of female and male *Dirofilaria immitis* using serial PNGase F and A digestion and separated into neutral and anionic pools (see Supplementary Figure 1 for workflow). Reflector mode MALDI-TOF–MS with or without permethylation (see Supplementary Figures 2 and 3) initially suggested a rather limited range of mannosidic glycans with Hex_3_HexNAc_2_Fuc_1_ (*m*/*z* 1135) as the major species, but linear mode MALDI-TOF–MS of the neutral pools indicated the presence of glycans of 5000 Da or more with series based on *Δm*/*z* 349 (corresponding to *N*-acetyl-hexosamine and fucose), which was simplified upon hydrofluoric acid treatment due to removal of antennal fucose (Supplementary Figure 2B and C). This is compatible with the occurrence of very large glycans with more than eighteen HexNAc residues on the antennae. Also, surprisingly for a nematode, anionic N-glycans appeared to be present forming a series of up to 4700 Da with *Δm*/*z* 203, which was an indication that these could also be based on multiple HexNAc residues; hydrofluoric acid treatment had, however, only a minor effect on the spectrum of the anionic pool, possibly due to less antennal fucose than on the neutral glycans (Supplementary Figure 2 E–H).

**Fig. 1:**
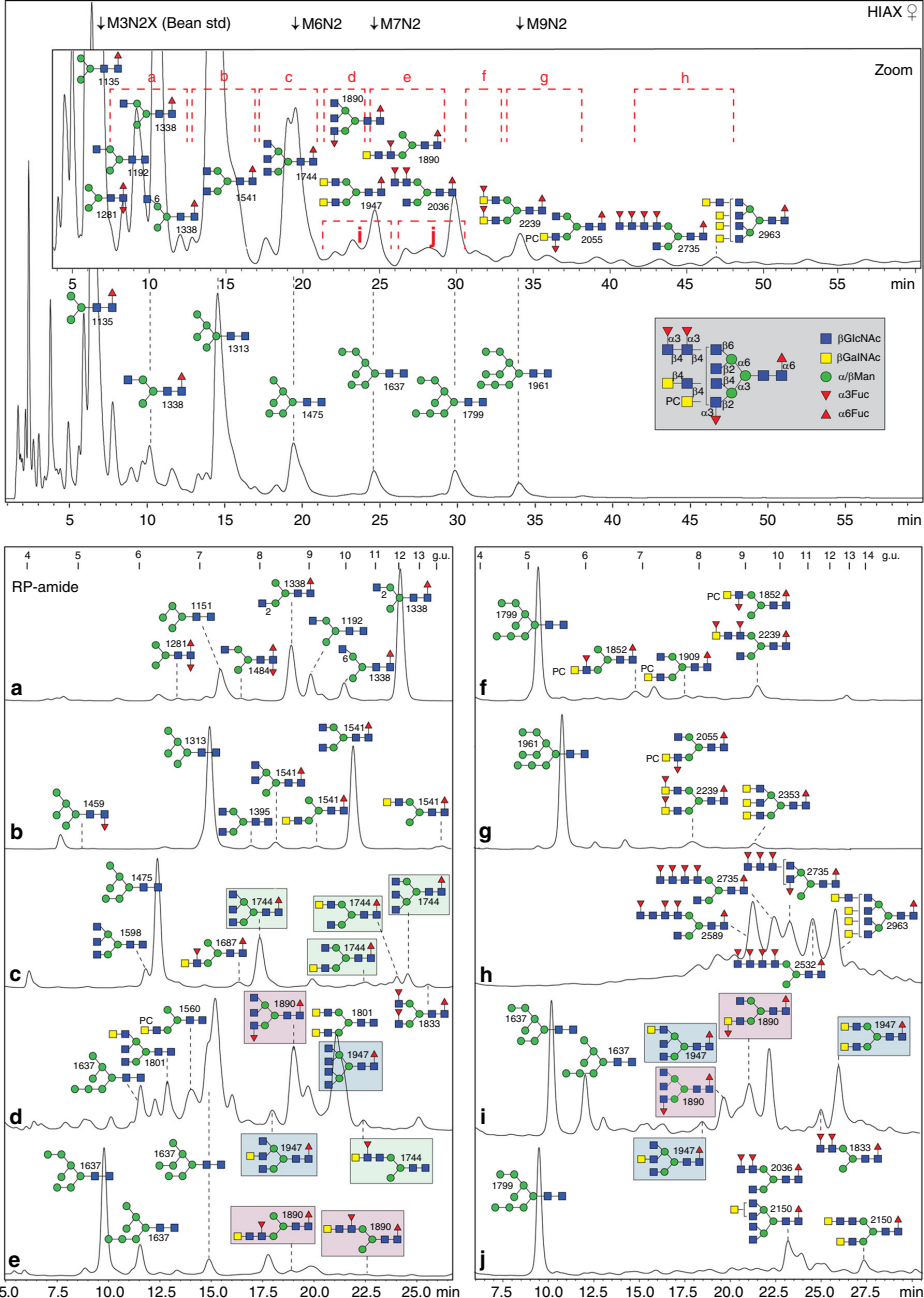
Two-dimensional HPLC fractionation of neutral N-glycans from *Dirofilaria immitis*. For the first dimension, the pyridylaminated N-glycans were separated by HIAX (calibrated with a set of glycans from white beans, e.g. Man_3_GlcNAc_2_Xyl_1_ and Man_6-9_GlcNAc_2_) and detected by fluorescence; shown in the top panel is the overall profile of one female N-glycome, whereas the zoom shows more detail of a second preparation. The male N-glycome had a similar profile (see also the MALDI-TOF–MS and 1D-RP-amide HPLC data in Supplementary Figures 2 and 4). Indicated are pooled regions whose second-dimension RP-amide HPLC profiles are shown in **a**–**j**, whereby chromatograms **a**–**c**, **i** and **j** are from the male glycome and **d**–**h** from a female preparation (note that subfraction **j** contains Man_8_GlcNAc_2_, but not Man_7_GlcNAc_2_ isomers as present in the subfractions **d**, **e** and **i** of overlapping retention time). The RP-amide chromatograms, calibrated in terms of glucose units (g.u.), are annotated with selected glycan structures according to the Symbol Nomenclature for Glycans (see also grey inset; mannose is depicted by green circles, GalNAc/GlcNAc by yellow or blue squares, fucose by red triangles and phosphorylcholine by PC, whereas the angle of the GlcNAc-Man bond for tri-/tetra-antennary structures is indicative of either a 2-, 4- or 6-linkage^70^). The *m*/*z* for the protonated ions as detected by MALDI-TOF–MS are also given with the depicted structures based on conclusions derived from elution time, chemical and/or enzymatic digestion and MS/MS data of which selected examples are shown in Figs. 2–4 and Supplementary Figures 5–9. The light green, pink and blue boxes highlight isomers of *m*/*z* 1744, 1890 and 1947 differing in the positions of antennal fucose and HexNAc residues. Based on integrated fluorescence intensity, the HIAX peaks eluting later than 22 mins are estimated to account for 8% of the total N-glycome, of which half (i.e. 4% of the total) are larger non-oligomannosidic structures of over 1800 Da with novel and/or multiple antennae

The different N-glycan pools (male and female, both neutral and anionic) were separately labelled by pyridylamination prior to HPLC; due to the poorly-resolved later regions of the RP-amide HPLC chromatograms containing many glycan species (see Supplementary Figures 4A/B and 11), a 2D-HPLC approach was employed. Thereby, a size/charge-based separation by HIAX was followed by a second dimension on the RP-amide column in order to achieve isomeric separation (Fig. 1). The structural proposals, summarised in various figures, are based on manually interpreted MS/MS data before and after chemical and enzymatic digests (for theoretical *m*/*z* values refer to Supplementary Tables 1 and 2).

### Paucimannosidic and other simple structures

In terms of MALDI-TOF–MS intensity and RP-amide HPLC data, the most abundant glycan is the paucimannosidic Man_3_GlcNAc_2_Fuc_1_ structure (*m*/*z* 1135 eluting at 9.2 g.u.; see also Supplementary Figures 4 and 5A and Supplementary Note 1). Additionally, other paucimannosidic, oligomannosidic, hybrid, pseudohybrid and small core di-fucosylated N-glycans were detected as well as one minor structure with galactosylated core fucose (see Fig. 1 and Supplementary Figure 5 B, C and E). As such glycans are very common in other nematodes (as well as invertebrates in general) and the RP-amide retention times in terms of glucose units as well as MS/MS data were comparable to those in previous studies^15–17^, there was no major effort to further re-characterise these.

### Antennal LacdiNAc and longer HexNAc-based extensions

**Fig. 2:**
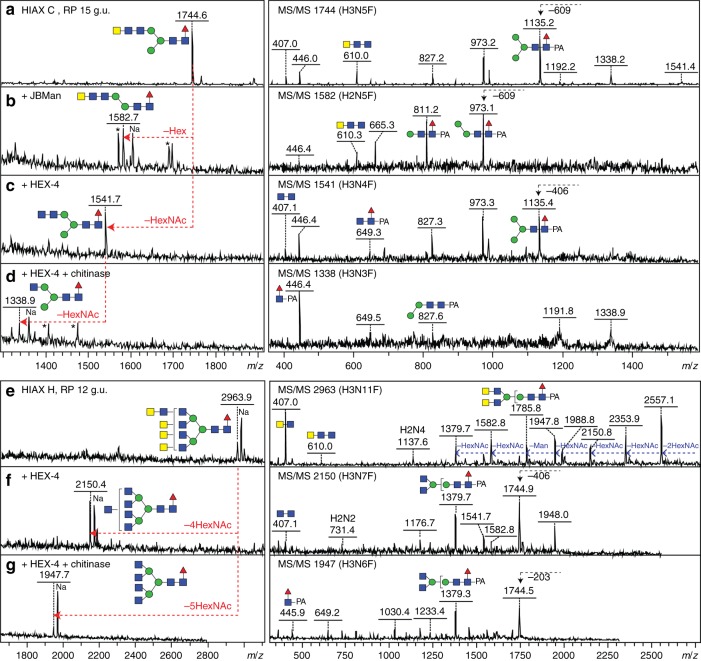
Example glycosidase digestions of N-glycans with tri- and di-HexNAc motifs. Pyridylaminated Hex_3_HexNAc_5_Fuc_1_ (*m*/*z* 1744) and Hex_3_HexNAc_11_Fuc_1_ (*m*/*z* 2963) glycans isolated in different 2D-HPLC fractions (Fig. 1, HIAX C and H) were subject to positive mode MALDI-TOF–MS (left) and MS/MS (right) before (**a**, **e**) and after treatment with either jack bean α-mannosidase (**b**), *C. elegans* HEX-4 (**c**, **f**) or *C. elegans* HEX-4 followed by *Streptomyces* chitinase (**d**, **g**). Contaminant peaks in enzyme digests (B and D; *m/z* 1570, 1689, 1696, 1407 and 1476) are indicated by asterisks. The MS/MS spectra are annotated with selected B- and Y-ion fragments (the latter, e.g. *m*/*z* 446, 973 or 1176, containing the reducing terminal PA label with the possible structure for the selected fragments based on the assumption of a single fragmentation event) as well as relevant serial losses (indicated in blue) or a single loss of 203, 406 or 609 from the parent (i.e. HexNAc_1-3_; indicated with black arrows); the loss of *m*/*z* 407 or 610 B-ions (HexNAc_2-3_) upon HEX-4 or chitinase digestions are indicative of removal of antennal GalNAc or GlcNAc residues. As defined by this and other data (including Supplementary Figures 6, 8 and 23, previous publications as well as controls with LacdiNAc and chitobiose-glycoconjugates and standard N-glycans), *C. elegans* HEX-4 is specific for β1,4-linked GalNAc and chitinase for either β1,4-linked HexNAc. Thereby, the nature of the HexNAc_2_ and HexNAc_3_ motifs of the *m*/*z* 1744 and 2963 glycans can be defined

In the overall neutral spectra of *D. immitis*, Fuc/HexNAc-based glycan series were found; indeed, both fucosylated and unfucosylated forms of LacdiNAc (GalNAcβ1,4GlcNAc) have previously been observed as a feature of some nematodes and proposed as modifications of N-glycans in *D. immitis* on the basis of lectin binding and monosaccharide composition^13^. 2D-HPLC could resolve multiple isomers of, e.g. Hex_3_HexNAc_4-6_Fuc_0-1_ (*m*/*z* 1541, 1744, 1801 and 1947). While some of these co-eluted with previously-defined bi- and tri-antennary structures decorated with single GlcNAc residues^16,18^, MS/MS of others resulted in signature B fragments at *m*/*z* 407 and 610 indicating the presence of two or three *N-*acetylhexosamines in series on one antenna (Fig. 2, Supplementary Figure 5 F–T and Supplementary Figure 6). For instance, it appears that Hex_3_HexNAc_5_Fuc_1_ (*m/z* 1744) can occur either in tri-antennary form (two isomers; with the upper arm β1,6-GlcNAc-modified one being the most common as opposed to small amounts of the later-eluting lower arm β1,4-GlcNAc-modified form; Fig. 1c) or in mono-/bi-antennary forms with HexNAc_2-3_ motifs; furthermore, one form of Hex_3_HexNAc_6_Fuc_1_ is tetra-antennary (*m*/*z* 1947; Fig. 1d). Also, the products of HF/chitinase digestion, which removed antennal fucose and serial HexNAc residues (Supplementary Figure 4D; see also below), show that more-or-less all possible combinations of HexNAc-containing antennae in *Dirofilaria* are based on hybrid, pseudohybrid, bi-, tri- and tetra-antennary structures.

Various hexosaminidases were used to test for the presence of certain *N-*acetylgalactosamine and *N-*acetylglucosamine modifications^19,20^. Most glycans with either an *m*/*z* 407 (HexNAc_2_) or 610 (HexNAc_3_) fragment were sensitive to HEX-4. In the case of HexNAc_3_ motifs, the terminal GalNAc was removed by *C. elegans* HEX-4, but the underlying residue only by the more unspecific *Streptomyces* chitinase (Fig. 2c, d, f and g). This led to the conclusion that GlcNAcβ1,4GlcNAc motifs can be capped with β1,4-linked GalNAc. On the other hand, *Xanthomonas* β-*N*-acetylglucosaminidase only removed unsubstituted β1,2-GlcNAc residues directly linked to mannose, which was useful in defining the antennal configuration (see, e.g., data in in Supplementary Figures 6–9 and Supplementary Notes 1 and 2).

### Fucosylated antennae

**Fig. 3:**
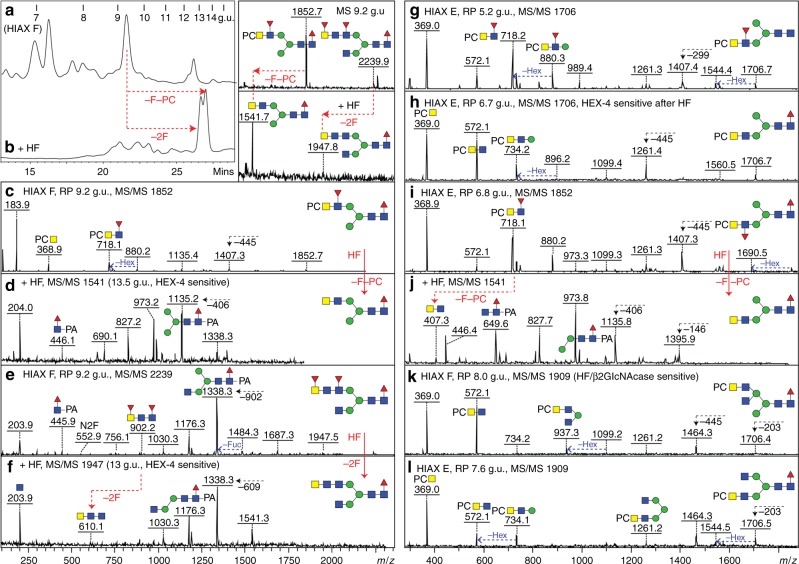
Analysis of example phosphorylcholine and fucose-modified N-glycans. **a**–**f** RP-amide HPLC, MALDI-TOF–MS and MS/MS analysis of an example 2D-isolated fraction before (**a**, **c**, **e**) and after (**b**, **d**, **f**) hydrofluoric acid treatment. A shift to higher elution due to altered hydrophobicity of the glycans (**b**) and alterations in the MS/MS spectra (removal of one fucose and one phosphorylcholine from *m*/*z* 1852 or of two fucoses from *m/z* 2239 correlating with losses of the *m*/*z* 184 (PC + H_2_O), 369 (GlcNAc_1_PC), 718 (HexNAc_2_Fuc_1_PC) and 902 (HexNAc_3_Fuc_2_) B-fragment ions) verify the proposed compositions, whereas the ability to remove one GalNAc from both with HEX-4 (data not shown) enabled the proposition of the isomeric structure. **g**–**l** MS/MS of other phosphorylcholine-containing glycans as well as one example (**j**) after hydrofluoric acid treatment; other sensitivities to this reagent or to hexosaminidases are noted. The contrast in the spectra of the two isomers of Hex_3_HexNAc_4_Fuc_1_PC_1_ (*m*/*z* 1706; **g**, **h**) is due to the position of the fucose residue. Selected B- and Y-fragments are annotated as well as certain losses of fucose or hexose; due to the excellent ionisation of PC-containing fragments (e.g. at *m*/*z* 718 and 572), the presence of core fucose is shown by the loss of 445 Da from the parent and by the presence of an *m*/*z* 446 Y-fragment observable after hydrofluoric acid treatment. The terminal position of phosphorylcholine is inferred by the minimal fragment containing both the PC and a hexose (i.e. *m*/*z* 734 [Hex_1_HexNAc_2_PC_1_], rather than *m*/*z* 531 [Hex_1_HexNAc_1_PC_1_] as in other nematodes). See Fig. 4 and Supplementary Figure 9 for further examples of data on phosphorylcholine-modified N-glycans

In nematodes, modifications of the N-glycan core chitobiosyl region with up to three fucose residues are known^20^; here, in the case of *Dirofilaria*, some of the *m*/*z* values indicated di- and tri-fucosylation (e.g. 1484, 1687, 1833, 1890, 2036 and 2239; Hex_3_HexNAc_3-6_Fuc_2-3_), but MS/MS of these revealed an *m/z* 446 Y-fragment indicative of only one fucose on the core (Fig. 3e and Supplementary Figures 5D, 7E/G, 8C/E/H and 9M). Thus, the second or third fucoses were presumed to be antennal, as confirmed by alterations in the MS/MS pattern upon defucosylation with hydrofluoric acid (i.e. loss of *m*/*z* 553, 699 or 902 B fragments; Fig. 3b, e and f and Supplementary Figures 7–9). As judged by the pattern of HEX-4 and chitinase resistance or sensitivity after hydrofluoric acid treatment, the underlying motifs were concluded to be normally GalNAcβ1,4GlcNAc motifs, but sometimes GlcNAcβ1,4GlcNAc was present (Supplementary Figure 8).

**Fig. 4:**
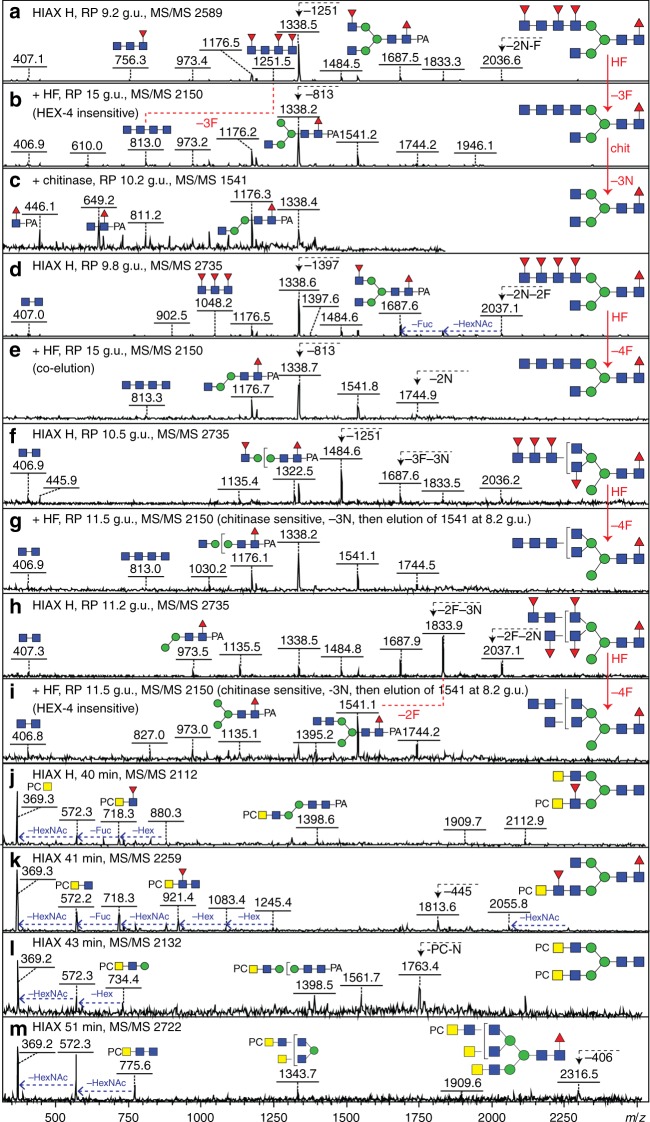
Analysis of larger fucosylated and phosphorylcholine-modified N-glycans. **a**–**i** Fucosylated glycans from HIAX pool H (see Fig. 1, 42-48 min) were separated by RP-amide HPLC prior to MALDI-TOF–MS/MS before (**a**, **d**, **f**, **h**) and after treatment with hydrofluoric acid (**b**, **e**, **g**, **i**) and hexosaminidases (example shown in **c**); the RP-amide retention times for the original and treated glycans (as glucose units) aid the structural interpretation. Hydrofluoric acid results in loss of three or four antennal fucose residues which correlates with loss of B-fragments such as those at *m*/*z* 756, 902, 1251 and 1397 and appearance of ones at *m*/*z* 610 or 813 (HexNAc_3-4_), whereas core fucosylated Y-fragments containing the PA label (*m*/*z* 973, 1176 and 1338) were unaffected. The HEX-4 insensitivity, but chitinase sensitivity, of the defucosylated products led to the conclusion that the HexNAc chains contain GlcNAc only and are not capped with GalNAc; uncertainty regarding the elongation of the 2- or 6-arm is indicated by a bracket (**f**–**i**). For all four glycans (**a**, **d**, **f**, **h**), loss of three or four Fuc yields glycans of *m*/*z* 2150 (see **b**, **e**, **g**, **i**) of different elution times and the subsequent removal of three GlcNAc residues (**c**, **g**, **i**) results in co-elution with two different *m*/*z* 1541 Hex_3_HexNAc_4_Fuc_1_ isomers (with either two β1,2- or one each of β1,2/6-GlcNAc residues eluting at 10.2 and 8.2 g.u.; compare with Fig. 1b, Supplementary Figures 4D and 5G/I). Example MS/MS of longer fucosylated glycans are shown in Supplementary Figure 10. **j**–**m** Phosphorylcholine-modified glycans from individual HIAX fractions were subject to MS/MS revealing PC substitution of HexNAc_2-3_ motifs with or without fucose; in comparison to other phosphorylcholine-modified glycans from this species (as shown by HEX-4 sensitivity after hydrofluoric acid treatment; see, e.g. Fig. 3, also for similar B-fragments such as *m*/*z* 572 and 718) and by the minimal fragment containing the phosphorylcholine and a hexose of the trimannosyl core, it is assumed that the zwitterion substitutes a terminal GalNAc, unlike the PC-GlcNAc motifs found in many other nematodes; selected losses and mass differences are highlighted

In later-eluting HIAX fractions (i.e. higher molecular weight regions), there were various glycans with four or more fucose residues; each *m*/*z* was detected in multiple 2D-HPLC fractions, which was indicative of many isomers being present (Fig. 1h). Hydrofluoric acid was again used to defucosylate these structures, prior to re-chromatography and/or hexosaminidase digestion. MS/MS of defucosylated glycans revealed HexNAc_2-4_-based fragments of *m*/*z* 407, 610 and 813 replacing fucosylated fragments at, e.g. *m*/*z* 756, 902, 1048 or 1251 (HexNAc_3-4_Fuc_1-3_); for instance, in the case of fractions containing species of *m*/*z* 2589 and 2735 (Hex_3_HexNAc_7_Fuc_4-5_), three or four fucose residues were removed with hydrofluoric acid resulting in a fragment of *m*/*z* 813 (compare Fig. 4a/b, d/e and f/g). Thus, also due to the HEX-4 resistance and chitinase sensitivity of the underlying HexNAc_4_ (Fig. 4c), the fucosylated antennae of these N-glycans were concluded to be based on a chito-oligomer.

Further larger glycans were detected, but due to relatively low amounts as well as isomeric diversity meant that exact structures could not be proposed (Supplementary Figure 10 A–C). However, for some glycans of between 3000 and 5000 Da, MS/MS data could be obtained before and after hydrofluoric acid treatment and were indicative of extended poly-*N-*acetylhexosamine chains modified with fucose residues (Supplementary Figure 10 D-H). In another approach, the entire pool of female N-glycans was separated by RP-amide HPLC after hydrofluoric acid treatment in order to remove antennal α1,3-fucose residues (Supplementary Figure 4C); a subset of these fractions were treated with chitinase resulting in a relatively simple chromatogram containing various hybrid, bi-, tri- and tetra-antennary glycans (Supplementary Figure 4D), which were assessed by various glycosidase digestions and MS/MS; the unusual *m*/*z* 1541 product, eluting at 8.2 g.u., with upper arm β1,2- and β1,6-linked GlcNAc residues is the basis for the polyfucosylated forms shown in Fig. 4. The cumulative dataset suggests that long fucosylated poly-HexNAc chains modify *D. immitis* glycans with up to four antennae.

### Phosphorylcholine-modified N-glycans

Phosphorylcholine modifications in nematodes are often associated with immunomodulation^12^ and a hallmark for this moiety on N-glycans is the B positive mode fragment at *m/z* 369 (HexNAc_1_PC_1_; Figs. 3, 4j–m and Supplementary Figure 9J); the next fragment in series was always one at *m*/*z* 572 (HexNAc_2_PC_1_), whereas other zwitterionic fragments included signals at *m*/*z* 718, 734 and 775 (respectively, HexNAc_2_Fuc_1_PC_1_, Hex_1_HexNAc_2_PC_1_ and HexNAc_3_PC_1_). Such fragment ions suggested that the phosphorylcholine was always terminally associated with HexNAc_2_, HexNAc_3_ or HexNAc_2_Fuc_1_ motifs, but not on GlcNAc residues directly modifying mannose. Hydrofluoric acid treatment was performed as this is known to efficiently remove phosphodiesters in addition to α1,3-fucose (Fig. 3). Together with re-chromatography and/or enzymatic digestions (e.g. HEX-4, chitinase or *Xanthomonas N*-acetylglucosaminidase; see, e.g. Supplementary Figure 9), the underlying structures could be fixed as being a range of bi- and tri-antennary or hybrid structures with terminal *N-*acetylgalactosamine, the latter being the site of substitution with the phosphorylcholine residue.

### Glucuronylated N-glycans

The big surprise in this study, in comparison to other nematodes, was the presence of anionic N-glycans for which a modification of 176 Da as compared to some of the neutral structures could be calculated; as the glycans were most easily detected in the negative-ion mode, it was assumed that this 176 Da modification is a hexuronic acid rather than a methylhexose. MS/MS resulted typically in an *m*/*z* 583 fragment in positive mode (*m*/*z* 581 in negative mode; HexA_1_HexNAc_2_), but also sometimes in one at *m*/*z* 786 (HexA_1_HexNAc_3_); this contrasts with the *m*/*z* 542 and 745 fragments observed with glucuronylated insect N-glycans^21^. Thus, the underlying residue was concluded to be a HexNAc rather than a β1,3-galactose as found in insects. As for the neutral pool, an initial trial with RP-amide HPLC alone indicated a poorly-resolved glycome (Supplementary Figure 11). Structures of up to nearly 5000 Da were detected after removal of antennal α1,3-fucose with hydrofluoric acid, suggesting that a non-fucosylated backbone of HexA_1_Hex_3_HexNAc_18_ was possible amongst monoglucuronylated structures (Supplementary Figure 2F and G). Furthermore, jack bean β-*N-*acetylhexosaminidase treatment of an aliquot of the entire anionic pool resulted in a major monoglucuronylated product (*m*/*z* 1918; Hex_3_HexNAc_5_Fuc_1_HexA_1_) with some residual antennal fucosylated structures remaining and subsequent bovine fucosidase digestion primarily resulted in the loss of a core fucose; re-chromatography enabled resolution of some major monoglucuronylated glycans (Supplementary Figure 11 A–C). Thus, it was assumed that a subset of anionic glycans contain rather long HexNAc-based chains which can be capped with glucuronic acid or are otherwise modified with fucose and, sometimes, phosphorylcholine.

**Fig. 5:**
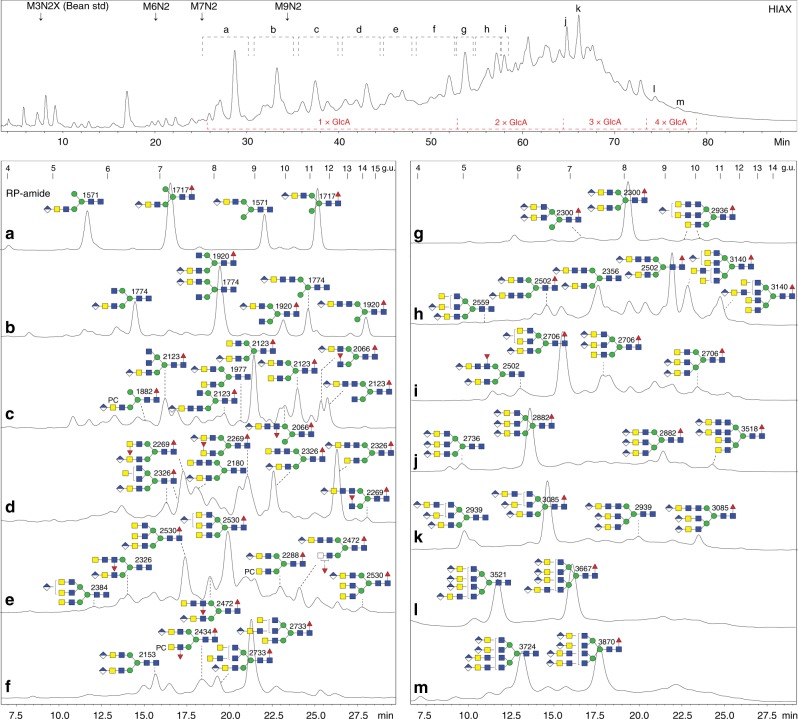
Two-dimensional HPLC fractionation of *Dirofilaria immitis* anionic N-glycans. As for the neutral preparations, the pyridylaminated N-glycans were separated by HIAX as calibrated with a set of glycans from white beans (Man_3_GlcNAc_2_Xyl_1_ and Man_6-9_GlcNAc_2_) and detected by fluorescence. Indicated are pooled regions whose second-dimension RP-amide HPLC profiles (normalised in terms of fluorescence) are shown in **a**–**m** as well as the regions in which mono-, di-, tri- and tetra-glucuronylated glycans elute from the HIAX column in a size- and charge-dependent manner. The RP-amide HPLC chromatograms, calibrated in terms of glucose units (g.u.), are annotated with glycan structures according to the Standard Nomenclature for Glycans (mannose is depicted by green circles, GalNAc/GlcNAc by yellow or blue squares, fucose by red triangles, glucuronic acid by diamonds and phosphorylcholine by PC) as well as the *m*/*z* for the [M+H]^+^ ions as detected by MALDI-TOF–MS; the depicted structures are based on conclusions derived from RP-amide elution time, chemical and/or enzymatic digestion and MS/MS data of which a selection is shown in Figs. 6–8 and Supplementary Figures 12–15. Overall MALDI-TOF–MS and 1D-HPLC profiles of an anionic glycan pool are shown in Supplementary Figures 2 and 11. Based on summed fluorescence intensities as compared to the neutral chromatograms, it is estimated that 5% of the N-glycome is in the anionic pool

**Fig. 6:**
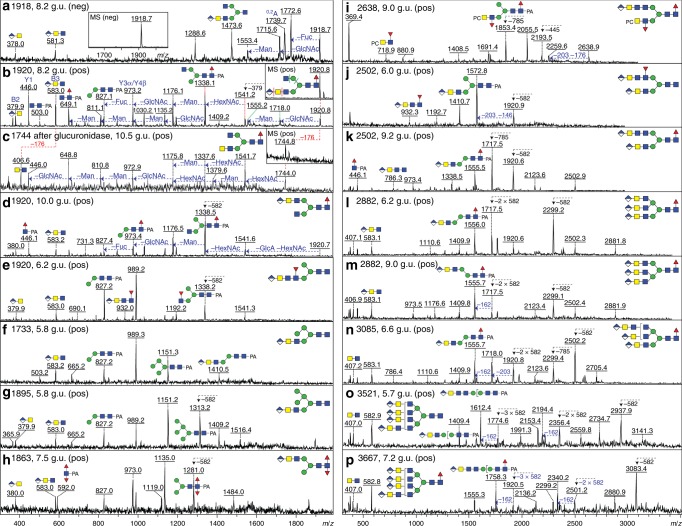
Example MS/MS data of glucuronylated N-glycans. **a**–**c** MALDI-TOF–MS/MS (with MS in insets) of the 2D-HPLC purified *m*/*z* 1918 (negative) or 1920 (positive) glycan eluting at 8.2 g.u. (Fig. 5b) before (**a**, **b**) and after (**c**) *Helix pomatia* β-glucuronidase treatment; removal of the glucuronic acid residue correlates with loss of 176 Da from the parent and of the positive mode B-fragment ion at *m*/*z* 583 (HexA_1_HexNAc_2_). In general, the positive ion mode MS/MS spectra of the protonated forms of glucuronylated glycans were more intense and more informative than the negative-ion mode MS/MS spectra, despite the excellent ionisation in negative mode of the parent species. **d**–**i** Positive mode MS/MS of monoglucuronylated N-glycans, including two isomeric forms of *m*/*z* 1920 differing in the position of the fucose residue (as shown by the *m*/*z* 932 HexA_1_HexNAc_3_Fuc_1_ fragment for one form), two hybrid, one core di-fucosylated and one phosphorylcholine-modified structures found in RP-amide fractions derived from HIAX anionic pools **b**, **c** and **h**; the presence of an upper arm antenna on the di-fucosylated glycan (**h**) is compatible with the requirement of nematode core α1,3-fucosyltransferase for an unsubstituted α1,3-mannose residue, whereas the *m*/*z* 592 Y-fragment defines the core difucose modification (see also Supplementary Figure 5B and C). **j**–**p** Positive ion mode MS/MS of 2D-HPLC-purified di-, tri- and tetra-glucuronylated N-glycans (see Fig. 5h, j–l). Losses of one, two or three HexA_1_HexNAc_2-3_ motifs (*Δm*/*z* 582 or 785) are indicated in addition to selected antennal B- and pyridylamino-containing Y-fragment ions. The concluded structures reflect the presence of corresponding non-glucuronylated tri- and tetra-antennary forms in the neutral pools with 4-linked GlcNAc leading to later RP-amide elution than a 6-linked GlcNAc. The two *m*/*z* 2502 glycans (**j**, **k**) differ in the location of the fucose residue, while the *m*/*z* 3521 glycan (**o**) elutes earlier than that of *m*/*z* 3667 (**p**) due to the retentive effect of the core α1,6-fucose; for further characterisation of bi-antennary forms including glycosidase digests, refer to Fig. 8 and Supplementary Figures 13–15

For a closer examination, the 2D-HPLC approach was employed to fractionate the anionic glycome, i.e. HIAX size/charge-based separation followed by RP-amide HPLC of selected pools (Fig. 5). Enzymatic digestion with *H. pomatia* β-glucuronidase was employed in time-limited digestions prior to re-chromatography. Thereby, glucuronic acid was shown to be removed from various example glycans resulting in neutral forms no longer ionisable in the negative mode and accompanied by loss of the *m/z* 583 B-fragment and appearance of one at *m*/*z* 407 (Fig. 6a–c and Supplementary Figure 12).

**Fig. 7:**
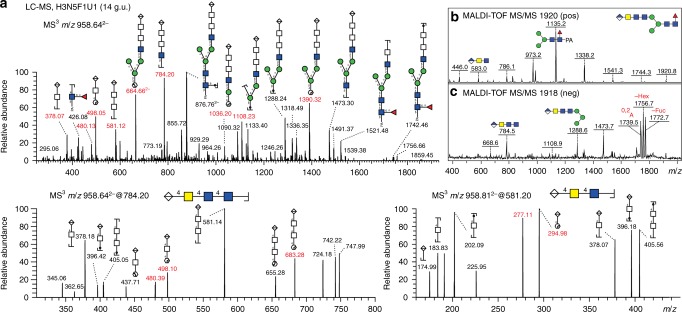
Negative mode LC-ESI–MS^*n*^ of a glucuronylated glycan. **a** The Hex_3_HexNAc_5_Fuc_1_HexA_1_ *m*/*z* 1920 glycan eluting at 14 g.u. (see also corresponding positive and negative mode MALDI-TOF–MS/MS data in **b** and **c**, whereby the latter shows some of the same non-cross-ring fragments as the ESI-MS/MS) and containing a HexA1-4HexNAc-4HexNAc-4GlcNAc motif as shown by MS^2^ of ions at *m/z* 958.64 (Hex_3_HexNAc_5_HexA_1_Fuc_1_-PA, [M−2H]^2−^) and MS^3^ of ions at *m*/*z* 784.20 (HexNAc_3_HexA_1_, [M−H]^−^) and at *m*/*z* 581.20 (HexNAc_2_HexA_1_, [M−H]^−^). Fragmentation ions at *m*/*z* 664 and 1390 were annotated as ^2,4^A and ^0,2^A cleavage of penultimate GlcNAc, both cleavages are diagnostic ions for N-glycans^69^. The presence of fragmentation ions at *m*/*z* 1036 (^0,3^A of βMan) and “D ions” at *m*/*z* 1090/1108 suggest the HexNAc_3_HexA_1_ motif linked to Man on the 6-antenna. No fragments are compatible with the presence of terminal HexNAc, while a series of B ions at *m*/*z* 378, 581 and 784 suggests a linear HexA-HexNAc-HexNAc linked to β1,2-linked GlcNAc on the 6-antenna. To determine the linkage between HexA-HexNAc-HexNAc, MS^3^ of the fragmentation ions at *m/z* 581, which contain HexNAc_2_HexA_1_, was performed. Fragments at *m*/*z* 295/277 (^0,2^A_HexNAc_/^0,2^A_HexNAc_-H_2_O) suggest terminal HexA linked to C_4_ of HexNAc; the ions at *m*/*z* 295 would be compatible with either a 3-, 4- or 6-linkage, but the m/z 277 fragment is most compatible with a 4-linkage. In addition, fragmentation ions at *m*/*z* 498/480 (^0,2^A_HexNAc_/^0,2^A_HexNAc_-H_2_O) indicate the presence of HexA-4HexNAc linked to C4 of HexNAc. Taken together, this special N-glycan antennal motif was annotated as a HexA-4HexNAc-4HexNAc sequence linked to the β1,2-linked GlcNAc on the 6-antenna

Even amongst the simple, monoglucuronylated forms, there were different isomers as exemplified by the RP-amide-separated structures of *m*/*z* 1774 and 1920 with either lower or upper arm (α1,3- or α1,6) antennal modifications being present (Fig. 5b); the *m*/*z* 1920 structures were, like many of the glucuronylated glycans, modified with one core α1,6-fucose as shown by the relatively late elution time of glycans with an *m*/*z* 446 Y-fragment (Fuc_1_GlcNAc_1_-PA; Figs. 6b, d and 7 and Supplementary Figure 12). Some structures were also of the hybrid type (*m*/*z* 1733 and 1895; Fig. 6f and g) and a further example (*m*/*z* 1863) was di-fucosylated on the core, as defined by the *m*/*z* 592 Y-fragment (Fuc_2_GlcNAc_1_-PA; Fig. 6h). In accordance with the presence of tetra-antennary glycans in the neutral glycome, the assumption that up to four glucuronic acid residues were present on *D. immitis* N-glycans was verified by the observation upon MS/MS in positive ion mode of serial losses of 582 or 785 Da (Fig. 6j–p).

An exact isomeric analysis of many anionic glycans was difficult due to the presence of four antennae based on HexNAc_2-3_ motifs with the maximal variations amongst the monoglucuronylated species. Based on the data on the neutral glycans, the presence of either GalNAcβ1,4GlcNAc or GalNAcβ1,4GlcNAcβ1,4GlcNAc motifs, some capped with glucuronic acid was assumed. In some cases, these motifs carried additional antennal fucose or phosphorylcholine residues, which could be removed by HF treatment with alterations in the RP-amide retention time and MS/MS spectra (Fig. 6e and i as well as Supplementary Figures 13 and 14G and H). Exemplifying the isomeric separation of such glycans, it was possible to identify multiple versions of *m*/*z* 2123 and 2326 (HexA_1_Hex_3_HexNAc_6-7_Fuc_1_; HIAX pools C and D as shown in Fig. 5) on the basis of MS/MS as well as of retention time shifts upon selected digestions with specific hexosaminidases to remove unsubstituted GalNAc residues (Supplementary Figure 14A-F and I-L; see also Supplementary Note 1).

**Fig. 8:**
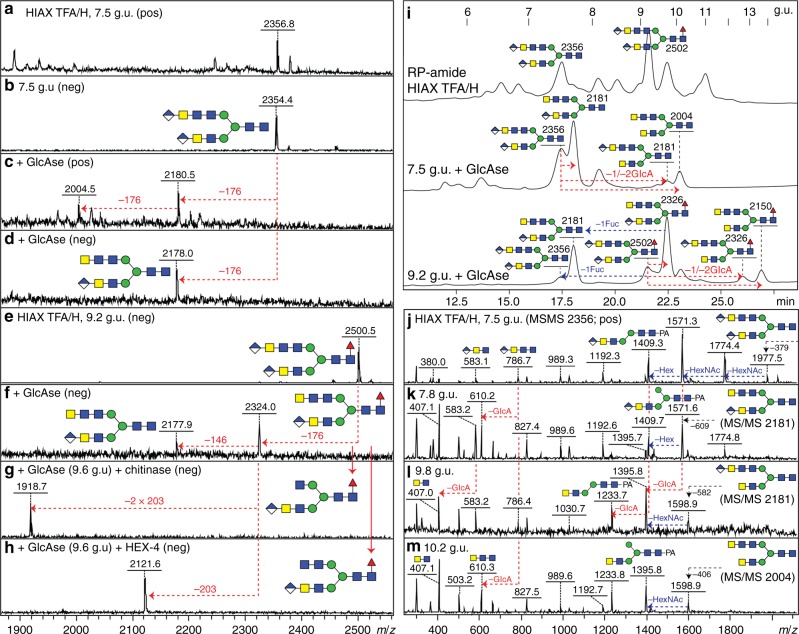
Effect of glucuronidase treatment on diglucuronylated N-glycans. **a**–**f** Two RP-amide HPLC example fractions from anionic HIAX pool H (see Fig. 5) were treated for 75 min with a commercial *H. pomatia* β-glucuronidase (GlcAse) prior to re-analysis by MALDI-TOF–MS (see also chromatograms in **i**) showing removal or one or two glucuronic acid residues (losses of 176 Da) as well as, due to an impurity in the enzyme preparation, partial defucosylation (loss of 146 Da). **g**, **h** Subsequent chitinase and HEX-4 treatment of the underlying exposed HexNAc_3_ motif followed by negative mode MALDI-TOF–MS reveals that the glucuronic acid on the upper antenna substitutes a GalNAcβ1,4GlcNAcβ1,4GlcNAc motif, which correlates also with LC–MS^*n*^ data (see Fig. 7). **i** Removal of glucuronic acid and fucose (as defined by changes in *m*/*z* as shown in **c**, **d** and **f**) correlates with shifts in RP-amide HPLC elution time as compared to the original chromatogram for anionic HIAX pool H (see arrows showing the forward shift caused by defucosylation (blue) and the backward shift after deglucuronylation (red), which is larger when the GlcA residue is lost from the lower arm); structures are annotated with *m*/*z* values for the [M+H]^+^ ions. **j**–**m** Positive mode MALDI-TOF–MS/MS of the 7.5 g.u. glycan before and after glucuronidase treatment revealing differences in fragmentation between the original glycan (**j**) and the partially (**k** and **l**) and fully deglucuronylated (**m**) products of different elution times; key changes in the B- and Y-fragments are indicated (e.g. loss of B-ions at *m*/*z* 583 and 786, appearance of ones lacking glucuronic acid at *m*/*z* 407 and 610 and shifts in the pyridylamino-containing Y-ions). For the MS/MS of the untreated and treated forms of the *m*/*z* 2502 glycan (refer to Fig. 6k and Supplementary Figure 15)

A final proof that glucuronic acid actually capped β1,4-linked *N-*acetylgalactosamine came from performing *H. pomatia* β*-*glucuronidase treatment of two bi-antennary biglucuronylated glycans followed by HPLC and further exoglycosidase digests. Under time-limited digestion conditions, primarily one glucuronic acid was removed, which resulted in shifts in retention time and in MS/MS fragmentation (Fig. 8). Subsequent linkage-specific HEX-4 and chitinase digestions led to the respective loss of 203 or 406 Da (i.e. of one or two *N*-acetylhexosamine residues), thereby verifying that the underlying HexNAc_3_ motif was based on GalNAcβ1,4GlcNAcβ1,4GlcNAc (Fig. 8g and h as well as Supplementary Figure 15). Also, LC-ESI–MS^*n*^ of glycans carrying one glucuronic acid indicated that the glucuronic acid is β1,4-linked to the underlying HexNAc_2-3_ motifs (Fig. 7 and Supplementary Figure 12); therefore, in combination with the MS^*n*^ data, it is concluded that GlcAβ1,4GalNAcβ1,4GlcNAcβ1,4GlcNAc represents the longest anomerically proven glucuronylated antenna in *D. immitis*.

### Glyco-epitopes in Dirofilaria adults and larvae

**Fig. 9:**
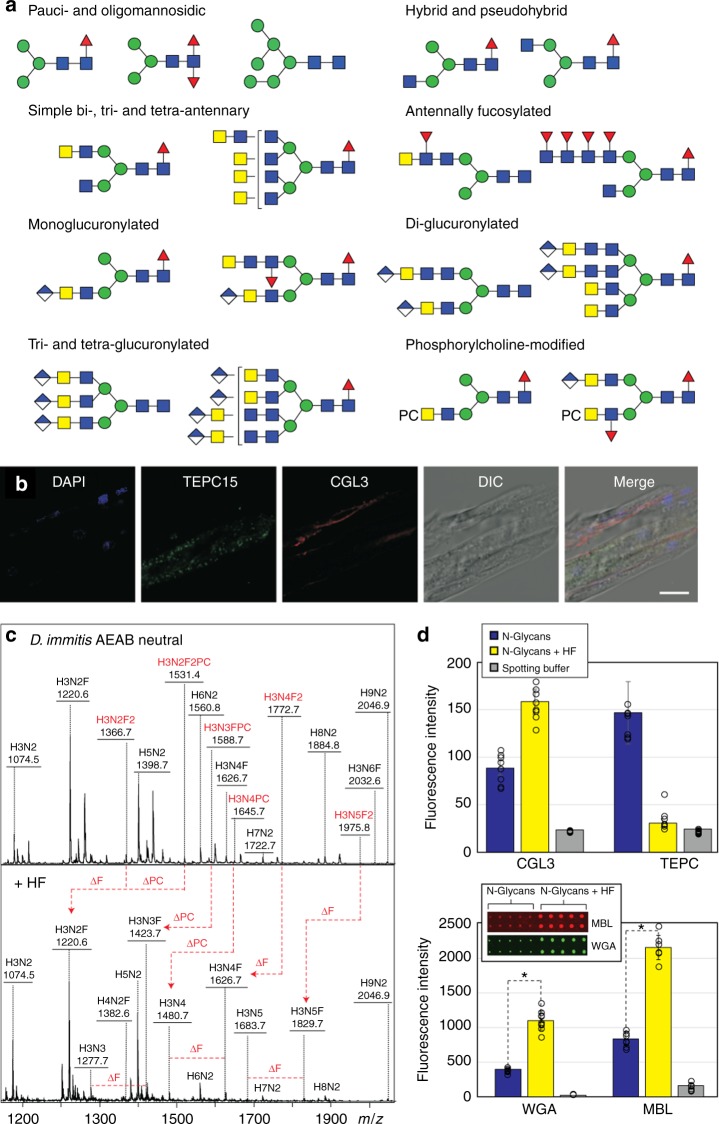
Glycan epitopes in *D. immitis*. **a** A summary of neutral and anionic N-glycan structures highlighting major antennal modifications; for a fuller set of defined structures refer to Supplementary Table 3. **b** The presence of HexNAc and phosphorylcholine residues in L3 larvae was probed using CGL3 (red) and TEPC 15 (green) by indirect fluorescence microscopy; also shown are the DAPI staining (for DNA) and DIC (differential interference contrast) images and the scale bar corresponds to 10 µm. **c** MALDI-TOF–MS of the AEAB-labelled neutral N-glycome before and after hydrofluoric acid treatment indicating loss of antennal fucose and phosphorylcholine residues. **d** The binding of *Coprinopsis* galectin (CGL3; 10 µg/ml), wheat germ agglutinin (WGA; 10 µg/ml) and human mannose binding lectin (MBL) to the *Dirofilaria* glycans increases after hydrofluoric acid treatment, while that to anti-phosphorylcholine (TEPC 15 IgA monoclonal) decreases. The charts indicate the uncorrected fluorescence values with the standard deviations (mean of 10 spots; analysed with an unpaired two-tailed parametric *t*-test with a 95% confidence level) as well as a negative control (spotting buffer only; example array scans are shown for the WGA and MBL data). Note that previous data indicate that CGL3 can recognise LacdiNAc, whereas WGA is known to bind HexNAc_*n*_ motifs and MBL to a wide range of glycans including those with terminal mannose or *N*-acetylglucosamine residues. Refer to Supplementary Figures 16–19 and 21 for (i) western blotting and further micrograph data (including controls) on epitopes recognised by CGL3 and TEPC 15, (ii) a summary of MS data on proteins affinity purified on CGL3- and TEPC 15-Sepharose, (iii) further MBL binding experiments (before and after endoglycosidase H treatment) to the immobilised glycome pools and fractionated immobilised glycans as well as data on other lectin interactions to the natural glycans and to defined di- and tri-saccharides

Based on the glycomic analyses, GalNAc/LacdiNAc, chitobiose, core/Lewis-type α1,3-fucose and phosphorycholine are potentially interesting epitopes represented in the neutral N-glycome (Fig. 9a and Supplementary Table 3). Therefore, selected blotting, histochemistry and glycan array experiments were performed; due to the reagents available and comparisons with other nematodes, an initial focus was on *N-*acetylhexosamine, fucose and phosphorylcholine epitopes. These should be, respectively, recognised by *Coprinopsis* CGL3 binding β-HexNAc^22^, *Coprinopsis* CCL2 recognising core α1,3-fucose^23^ and TEPC 15, a monoclonal IgA known to bind phosphorylcholine^24^ and indeed a wide range of proteins cross-reacted with these reagents in both male and female extracts (Supplementary Figure 16). Other indications for the presence of various nematode glyco-epitopes came from histochemical fluorescence microscopy of L3 larvae, which showed that (i) CGL3 bound exclusively to the surface of internal worm organs, (ii) CCL2 recognised structures in all tissues and (ii) TEPC 15 staining presented a dotted pattern in the gastrointestinal tract (Fig. 9b and Supplementary Figure 16). An indication that CGL3 and TEPC 15 actually recognised *Dirofilaria* proteins is the mass spectrometric identification of a predicted parasite glycoprotein following affinity enrichment with either of these two reagents (Supplementary Figure 17).

The presence of certain epitopes specifically on the N-glycans was tested in a glycan array format. The abundant neutral N-glycan pool was labelled with AEAB and immobilised either before or after hydrofluoric acid treatment (Fig. 9c and Supplementary Figure 18). We tested their binding to the aforementioned fungal lectins (CGL3 and CCL2), plant lectins (wheat germ, tomato and *Aleuria aurantia* lectins as commercially-available surrogates to test interactions with *N-*acetylhexosamine and fucose residues) and human mannose binding lectin MBL which has a range of glycan ligands^25–27^; concanavalin A and *Aleuria aurantia* lectin were also used to screen HPLC-fractionated AEAB-labelled N-glycans (Supplementary Figure 19). Generally binding to lectins was higher after chemical removal of most antennal α1,3-fucose and phosphorylcholine residues, while that to TEPC 15 decreased as expected and the calcium-dependent interaction with MBL was reduced upon endoglycosidase H treatment suggestive of binding to oligomannosidic and hybrid N-glycan structures (Fig. 9d and Supplementary Figure 18).

### Immune-relevant N-glycan epitopes

**Fig. 10:**
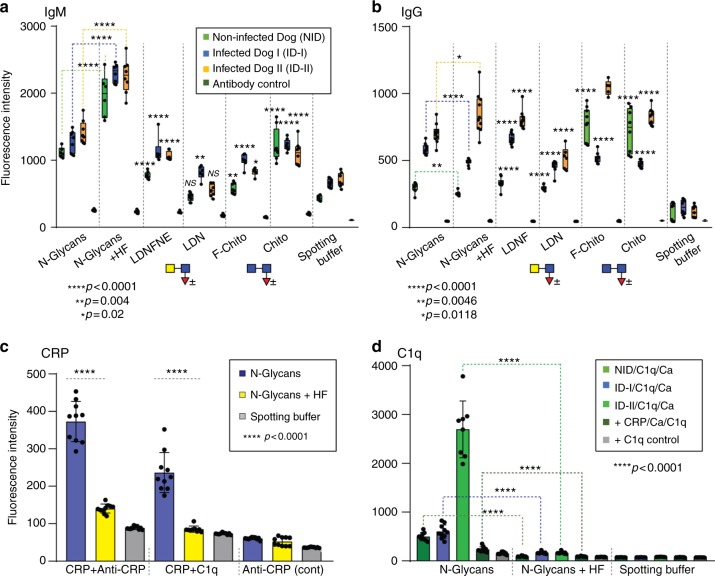
Binding of dog antibodies and C-reactive protein to *D. immitis* N-glycans. **a**, **b** The binding of IgM and IgG antibodies in three different dog sera (1:250 diluted; non-infected dog, infected dog I and infected dog II) were tested toward natural AEAB-labelled *Dirofilaria* N-glycans before and after hydrofluoric acid treatment, as well as reference 6-(5-aminopentanamido)-*N*-(2-[2-[oligosaccharyl-*N*-methoxyamino]ethoxy)ethyl]-2-naphthamides (see Supplementary Figure 23), which resemble selected terminal carbohydrate modifications of *D. immitis* glycans, i.e. fucosylated LacdiNAc (LDNF), LacdiNAc (LDN), fucosylated chitobiose (F-chito) and chitobiose (chito; see also symbolic depictions). IgM binding toward the HF-treated glycan pool was stronger than to the natural glycan pool and was also significant towards LDNF, chitobiose and F-chito; the pattern of IgG binding was more variable, but was highest towards N-glycans and LDNF for infected dogs. **c** The binding of human pentraxin C-reactive protein (5 µg/ml CRP; detected either with anti-CRP or with C1q/anti-C1q) was examined by probing the AEAB-labelled N-glycan pool (before or after hydrofluoric acid treatment) on NHS-modified glass slides as compared to relevant negative controls (spotting buffer only or anti-CRP alone); the binding of C-reactive protein decreased after chemical removal of phosphorylcholine. **d** The binding of exogenous complement C1q (10 µg/ml) in the presence of Ca(II) ions (5 mM) toward natural dog CRP interacting with *Dirofilaria* N-glycans was also tested using the three different sera (1:250 diluted). The strongest response was detected for the infected dog serum II (ID-II) compared to the other dog sera (control serum NID or infected dog ID-I) or the controls with no dog sera (+CRP/C1q or +C1q). The presented data are typical of the three experiments performed per binding combination and are based on uncorrected fluorescence values with standard deviations (mean of 10 spots); significance levels (analysed unpaired two-tailed parametric *t*-test at α 0.05) are shown by either dashed lines or asterisks. For further array-related data, including the HPLC chromatograms and example MS/MS of AEAB-labelled glycans, data on binding of CRP and TEPC 15 to sub-fractionated N-glycans and controls for CRP or C1q interactions in the presence of EDTA, refer to Supplementary Figures 18–21

In order to test whether *D. immitis* glycan epitopes are the targets of either the innate or adaptive immune system, arrayed glycans were also probed with either dog immunoglobulins or human C-reactive protein. Binding of natural dog IgM (regardless of infection status) to fucosylated LacdiNAc (LDNF) and chitobiose was significantly above background levels for all three sera, but was higher for the chemically stripped *Dirofilaria* glycans than for the native structures (Fig. 10a); on the other hand, pronounced IgG binding to natural N-glycans as well as to fucosylated LacdiNAc was detected only for the infected dogs, while IgG from the control and infected dogs also bound fucosylated and non-fucosylated forms of chitobiose (Fig. 10b).

The binding of C-reactive protein (CRP), an acute phase protein known to bind zwitterionic moieties and to elicit complement activation, to *D. immitis* glycans decreased after hydrofluoric acid treatment (Fig. 10c). CRP-dependent binding of the complement component C1q to the natural, untreated glycans was also observed (Fig. 10c and Supplementary Figure 20A), as was binding of endogenous CRP in dog sera to native glycans (Supplementary Figure 20B). On the other hand, in the case of one of the infected dog sera, indirect calcium-dependent binding of C1q^28^ to the natural glycans on the array was seen (Fig. 10d and Supplementary Figure 20C). Significantly, regardless of whether anti-CRP or C1q were used for detection, the binding of CRP to *Dirofilaria* glycans was highest to the larger molecular weight N-glycans as judged by arraying after NP-HPLC of the AEAB-labelled N-glycome; the same fractions, containing Hex_3_HexNAc_7-9_Fuc_0-1_PC_2_, also had the most significant binding to the TEPC 15 antibody, thus verifying that CRP binding correlated with the presence of glycans with two phosphorylcholine residues (Supplementary Figure 21).

## Discussion

It is a common assumption that lower eukaryotes have simple N-glycans; however, the data accumulated in recent years challenge this view^29^. Indeed, the present study exemplifies that a nematode can produce N-glycans with as many antennae as known in mammals and, using 2D-HPLC with MS/MS before and after chemical/enzymatic digestion, a large and diverse range of glycans can be proposed. The ~150 verified compositions (see Supplementary Tables 1-3 and the summary in Fig. 9a) is a number which not only hides the multiple isomers for many masses, but is definitely an underestimate considering the limitations of identifying and fragmenting the larger mass glycans of 3000 Da or more. Some variations between the two preparations of female N-glycomes were observed (e.g. a tendency for more phosphorylcholine in the second preparation) as well as between male and female worms as evidenced by the relative HPLC and MS peak intensities (e.g. Supplementary Figures 2 and 4); however, due to the complexity of the glycome, it is difficult to discern an obvious degree of gender difference as previously noted for the glycome of another parasitic nematode^20^. Thus, the overall similarity of chromatograms and spectra suggests that we have identified the typical N-glycosylation pattern of this species; revealing the most interesting features of both the neutral and anionic N-glycomes by off-line LC-MALDI-TOF–MS is another demonstration of the power of our approach to deeply mine the glycomes of invertebrates to reveal species-specific patterns of glycosylation.

Previously, in addition to neutral structures, only zwitterionic and not anionic N-glycans were found in nematodes^17^. Thus, the outstanding feature of the *D. immitis* N-glycome is not just the long fucosylated antennae on neutral glycans, but also the presence of branched and elongated glucuronylated oligosaccharides (5% of the total), which could be identified as containing GlcAβ1,4GalNAcβ1,4GlcNAc motifs; the minimal glucuronylated structure of Hex_3_HexNAc_4_GlcA_1_ suggests that LacdiNAc would prove a suitable substrate for a *D. immitis* β-glucuronyltransferase. While long chito-oligomers have, at least after hydrofluoric acid treatment, been previously found in other filarial species^30^, the glucuronylated series represent a new variant of nematode N-glycan modification, which is reminiscent (but not identical) to N-glycans from insects which are based on GlcAβ1,3Galβ1,3GalNAc/GlcNAc^18,21^. The exact linkage as found here is also unlike the GlcAβ1,3GalNAcβ of chondroitins, the GlcAβ1,4GlcNAcα of heparans, the GlcAβ1,3Galβ of the HNK-1 epitope or the β1,3-linkage previously found in *C. elegans* O-glycans^31–33^. It may be that glucuronylation is the closest the parasite can metabolically get to the sialylation of the host, as there is certainly a supply of UDP-GlcA in the nematode Golgi in order to enable synthesis of such anionic glycan structures.

Other than glucuronic acid, fucose and phosphorylcholine can also substitute *N-*acetylgalactosamine on *D. immitis* N-glycans; however, the only double substitution is a relatively rare GlcA_1_PC_1_GalNAc_1_ unit (see Supplementary Figure 13C). Otherwise, *N-*acetylgalactosamine is a relatively common cap and may constitute a stop signal during the extension of chito-oligomer-based antennae in this species. It is also interesting to note which features the *D. immitis* N-glycome does not display as compared to other nematodes: there is no sign of a fucose on the distal core GlcNAc^15,20,34^ nor of bisecting fucosylated galactose^35^, neither modification being biosynthetically compatible with the presence of four antennae. Also, galactosylation of core α1,6-fucose (GalFuc^36^) is represented by a single detected glycan, while neither further substitution of the GalFuc motif nor methylation of any fucose residue was detected in *D*. *immitis*.

The initial MALDI-TOF–MS screening of the neutral glycan pools suggested that structures of 3000 Da and more were present; examination of HIAX fractions (separating the neutral glycans on the basis of size) showed signals of around even 7000 Da. The size is thereby striking, although not as high as the 18,000 Da proposed for large poly-LacNAc-containing N-glycans from *Trypanosoma brucei*^37^. However, due to adducts and a mixture of fucose and phosphorylcholine modifications, a clear picture of the maximal glycan size in *D*. *immitis* was not possible, but the presence of long decorated HexNAc oligomers is reminiscent of the chito-oligomers of another filarial worm (e.g. *Onchocerca*). Also, the occurrence of four antennae has been proposed for glycans from, e.g. *Acanthocheilonema* and *Trichinella*^30,38^, whereas only maximally three antennae are observed in *Caenorhabditis* or its relatives^16^. This would correlate with the presence of *N-*acetylglucosaminyltransferase I, II, IV and V homologues in filarial worms, but the lack of a *N-*acetylglucosaminyltransferase IV in the Rhabditae (unpublished homology searches). The activities of both *N-*acetylglucosaminyltransferases IV and V results in a mixture of isomers of tri-antennary structures, which can either have GlcNAc β1,2 and β1,4-linked to the α1,3-mannose and β1,2-linked to the α1,6-mannose or have solely β1,2 on the α1,3-arm and both β1,2 and β1,6 antennae on the α1,6-arm. The predominance of β1,6- over β1,4-linked GlcNAc amongst the tri-antennary structures (estimated as a 3:1 ratio, also when considering the chemically defucosylated structures; see Supplementary Figure 4), as well as the presence of tetra-antennary forms, is in keeping with an older model for *D*. *immitis* glycans^13^. It is probable that the largest glycans are due to elongation of all four arms.

The *D*. *immitis* N-glycome is rich in non-mammalian glycan motifs, all of which could be immunogenic: core and antennal α1,3-fucose, phosphorylcholine and glucuronic acid capping. Whereas core α1,3-fucose is an IgE epitope in *Haemonchus*-infected sheep^39^, as well as being a cross-reactive determinant of bee venom and various plant allergens^40^, anti-glycan antibodies are known to bind antennal fucosylated epitopes in unrelated trematode *Schistosoma* species, whereby the presence of LacdiNAc with and without fucose is shared^41^, but *D. immitis* shows no sign of chains of fucose linked to fucose. Phosphorylcholine is recognised by human C-reactive protein^42^ and is a known immunomodulatory epitope in the case of the *Acanthocheilonema* ES-62 protein^43^. Glucuronylated N-glycan structures based on a different motif have been recently found to be a common element in glycomes of Diptera, Lepidoptera and Hymenoptera^21,44^, but the immunological repercussions of glucuronylation of proteins expressed in insect cell lines is unknown. Schistosomes also express an unusual glucuronic acid containing oligosaccharide, but this is an immunoreactive O-glycan^45^.

In terms of infection, it is the glycome of the insect-borne L3 larvae which is most relevant; certainly, microscopy experiments indicate that LacdiNAc, phosphorylcholine and core/antennal α1,3-fucose epitopes are present in the larvae, but unfortunately the amounts of L3 material were insufficient for a glycomic analysis; however, older work revealed the presence of LacdiNAc-containing and oligomannosidic structures in the microfilariae derived from canine blood, which is the stage taken up by the insect vector^13,14^. As there are no massive glycomic shifts between L3 and adult in another nematode species^20^ and at least some adult epitopes can be found in the larvae by indirect histochemical means, we assume the adult glycome contains glycan motifs already present during the stage infective to mammals. We can only speculate as to whether the glucuronylated N-glycans of *D. immitis* have roles in the interactions between the parasite and the insect vector, which expresses similar (but not identical) anionic motifs^18^.

Using the adult N-glycome, we developed the first natural N-glycan array for a parasitic nematode complemented by synthetic conjugates and tested for binding to different reagents including pentraxins, lectins and antibodies. C-reactive protein is a potential marker for pulmonary hypertension in canine dirofilariasis or for chronic lymphatic pathology in human wuchereriasis^46,47^ and so interactions affecting the ability of this pentraxin to elicit or modulate a response are biologically relevant. Thus it is interesting that C-reactive protein recognised the natural parasite N-glycans on the array, either when using anti-CRP or C1q/anti-C1q for detection, especially higher molecular weight fractions containing glycans predicted to carry two phosphorylcholine residues (Fig. 10 and Supplementary Figure 21); the estimated 45 Å distance between longer glycan antennae is similar to that between the phosphorylcholine-binding sites of the pentameric C-reactive protein (Supplementary Figure 22) and so could explain the highest binding of this pentraxin to the fractions containing glycans with two phosphorylcholine residues. It is also interesting to make a comparison with bacterial polysaccharides, which can also contain one or more phosphorylcholine residues^48^; indeed the expression and exact position of this modification correlates with both the persistence of *Haemophilus* in the upper respiratory tract and its CRP-dependent killing^49,50^. However, whereas bacteria can escape complement by phase variation in the expression of phosphorylcholine, the strategy of nematodes is different: it may be the flexible conformation of their multiantennary N-glycans carrying one or more phosphorylcholine residues, as found on the aforementioned ES-62 protein^30^, which mediate binding by CRP in a manner resulting in inefficient complement activation at the stage of C2 cleavage^51^.

Not just CRP-dependent binding of C1q, but also binding of C1q mediated by dog serum to the arrays were observed, which could be due to either endogenous CRP or IgG. However, as C1q has potential immunoregulatory and physiological effects other than complement activation^52^, we cannot yet conclude whether this binding would elicit or inhibit downstream responses in vivo. Complement can also be activated by MBL and indeed microfilariae of *Brugia malayi* survive longer in MBL-A-deficient mice^53^; as endoglycosidase H is known to cleave oligomannosidic and hybrid glycans^54^, its impact on MBL binding to natural glycans can be explained (Supplementary Figure 18 and 21A). We can, though, only speculate why this enzyme abolished the increased binding to *D. immitis* N-glycans printed after hydrofluoric acid treatment, unless HF-stripped versions of the observed PC-/Fuc-modified hybrid glycans are also MBL ligands; however, low affinity binding to fucosylated structures has been previously seen on a standard glycan array^27^.

The available dataset also suggests that, regardless of the infection status, the native antennal modifications may even inhibit binding of IgM antibodies as these bind preferentially to *Dirofilaria* N-glycans when the antennal fucose and phosphorylcholine residues are removed (Fig. 10a). Binding to a chitobiose conjugate was detected in all three sera, which is consistent with natural anti-chitobiose IgM and IgG being found in sera of healthy mice and humans^55^. Interestingly, β1,6-poly-GlcNAc polymers (i.e. isomeric forms of the filarial-type β1,4-chito-oligomers) expressed by some bacteria, protists and fungi are also recognised by natural antibodies^56^. In contrast, IgG binding to the natural glycans as well as to a fucosylated form of LacdiNAc (i.e. LDNF) was highest for the two infected sera (Fig. 10b), although the difference in binding as compared to non-fucosylated LacdiNAc is not as obvious as for *Trichinella-*infected humans^57^; other studies reveal that various mono- and di-fucosylated LacdiNAc-containing glycans are also recognised by antibodies of *Schistosoma*-infected animals^58,59^.

Overall it can be stated that at least a portion of the neutral and anionic N-glycans are of extreme size and charge and modelling suggests rather extended conformations with distances between the antennae being estimated as 45 Å (Supplementary Figure 22). Although shorter than those between antigen-binding sites in IgG, this is within the shortest distance between the binding sites of some multivalent lectins^60^ as well as between the protomers of C-reactive protein^61^; thereby, the large glycans of *D. immitis* may act as a type of natural glycodendrimer. Thus, we can hypothesise that the modifications of *D. immitis* glycans may cross-link some proteins of the innate immune system, as suggested above for C-reactive protein, but prevent binding to others, including natural IgM. It may be that some heartworm glycoproteins, whether membrane-bound, excretory–secretory products or on exosomes^62^, act as a barrier preventing access of antibodies, lectins or other proteins to the external and internal surfaces of the nematode during its life in the mammalian host; alternatively, they may inhibit host elicitor functions or indeed be required for completion of the infection cycle. Thereby, our glycomic and array data could be an indication that glycomimickry or glycogimmickry (i.e. presenting either host-like glycans engendering invisibility or rather unusual glycans which are immunomodulatory) are, as proposed for other helminths^63^, potential mechanisms during *Dirofilaria* infection.

## Methods

### Biological material

*Dirofilaria immitis* adults were isolated upon surgical heartworm removal from pulmonary arteries (via the left jugular vein using flexible alligator forceps) of dogs whose infection was originating either in Italy or in Thailand; the dogs were privately owned and underwent surgery for therapeutic reasons. The female worms could be identified due to their larger size and the shape of the tail whereas the males are smaller and have a coiled tail. Approximately 2 g (wet weight) of worm material were used for each preparation, which corresponds to 10 female or 20 male worms. Dog blood containing *D. immitis* microfilariae used as the source of infected sera for glycan array analyses as well as live L3 larvae were obtained from BEI Resources, Manassas, VA.

### Enzymatic release and purification of N-glycans

The worms were lyophilised before grinding in liquid nitrogen and nematode homogenates were proteolysed with thermolysin^64^, prior to cation exchange and gel filtration chromatography of the proteolysate. Thereafter, N-glycans were released from glycopeptides using peptide:N-glycosidase F (recombinant PNGase F; Roche) as previously described^64^; the pH was adjusted to pH 4 and peptide:N-glycosidase A (recombinant almond PNGase A, in-house His-tag purified recombinant form expressed in insect cells) added and digestion continued for another 24 h. After an initial purification by cation-exchange chromatography (Dowex AG50; flow-through), the glycans were subject to solid-phase extraction on non-porous graphitised carbon (SupelClean ENVICarb, Sigma-Aldrich) as described^64,65^; the neutral and anionic-enriched fractions were subsequently eluted with (i) 40% acetonitrile and (ii) 40% acetonitrile containing 0.1% trifluoroacetic acid, respectively. The pools of glycans were then subjected to a second solid-phase extraction on C18 and the glycans in the flowthrough and 15% methanol elution were pooled and labelled via reductive amination using 2-aminopyridine (PA)^64^. Refer to Supplementary Figure 1 for the workflow as well as for Supplementary Note 2 further explanations regarding the glycomic analyses and assignments. The female glycomes were prepared twice: the first female preparation and the male preparation were subject to RP-amide separation as 1D-HPLC as well as 2D-HPLC, whereas the second female preparation was subject to 2D-HPLC only; there were no major differences between the female and male glycomes and the second female preparation. There was no evidence for canine glycans in the prepared glycomes as judged by the lack of sialylated, α-galactosylated or bisected LacNAc-modified glycans previously detected in dogs^66,67^; also, the fucosylated chitobiose core regions of the glycans detected would not be compatible with contamination from a bacterial source.

### HPLC fractionation

For 1D-HPLC, complete pyridylaminated N-glycomes were fractionated by reversed-phase HPLC (Ascentis Express RP-amide from Sigma-Aldrich; 150 × 4.6 mm, 2.7 µm) and a gradient of 30% (v/v) methanol (buffer B) in 100 mM ammonium acetate, pH 4 (buffer A) was applied at a flow rate of 0.8 ml/min (Shimadzu LC-30 AD pumps) as follows: 0–4 min, 0% B; 4–14 min, 0–5% B; 14–24 min, 5–15% B; 24–34 min, 15–35% B; 34–35 min, return to starting conditions^16^. The RP-amide HPLC column was calibrated daily in terms of glucose units using a pyridylaminated dextran hydrolysate and the degree of polymerisation of single standards was verified by MALDI-TOF–MS^64^. Alternatively, hydrophilic interaction anion exchange (HIAX) HPLC for size/charge separation was performed with an IonPac AS11 column (Dionex; 4 × 250 mm) using a Shimadzu Nexera UPLC system as described previously^65^. A two solvent gradient was applied with buffer A (0.8 M ammonium acetate, pH 3.85) and buffer B (80% acetonitrile) at a flow rate of 1 ml/min: 0–5 min, 99% B; 5–50 min, 90% B; 50–65 min, 80% B; 65–85 min, 75% B. A pool of pyridylaminated oligomannosidic N-glycans from white beans (containing Man_3-9_GlcNAc_2_) was used to calibrate the column. RP-amide and HIAX glycan fractions were collected manually based on observation of the fluorescence intensity (excitation/emission at 320/400 nm; Shimadzu RF 20 AXS detector) and analysed by MALDI-TOF–MS and MS/MS. For 2D-HPLC, HIAX fractions, selected on the basis of the mass spectrometric data, were re-chromatographed on the RP-amide column. In total, over 120 HPLC runs on whole glycomes, sub-pools and digested fractions were performed. Thereby, isomeric separation of many glycans could be attained; as shown in previous studies using RP-HPLC as well as by use of standards such as asialoagalacto N-glycans derived from bovine fetuin, tri-antennary isomers can be separated with the 2/4/2-substituted forms eluting later than those with 6/2/2; also, RP-HPLC can resolve glycans carrying a single GlcNAc on the α1,3-mannose as opposed to those with one on the α1,3-arm^68^.

### Mass spectrometry

MALDI-TOF–MS was performed using an Autoflex Speed (Bruker Daltonics, Bremen) instrument in either positive or negative reflectron modes with 6-aza-2-thiothymine (ATT; Sigma-Aldrich) as matrix; samples (0.8 µl) were vacuum dried on ground or polished steel plates before addition of matrix (0.8 µl of 3 mg/ml ATT in 50% ethanol) and crystallisation again under vacuum^64^. The matrix region was suppressed and so MS spectra were normally recorded in the range *m*/*z* 700-3500, but as required up to 4500 in reflector mode or higher in linear mode; for some samples, the lens voltage was decreased either to facilitate detection of low abundance glycans or to reduce in source fragmentation of structures with multiple anionic residues. MS/MS was in general performed by laser-induced dissociation of the singly charged [M+H]^+^ or [M-H]^-^ ions (selection window typically 0.6%); typically 1000 shots were summed for MS and 5000 for MS/MS. Spectra were processed with the manufacturer’s software (Bruker Flexanalysis 3.3.80) using the SNAP algorithm with a signal/noise threshold of 6 for MS (unsmoothed) and 3 for MS/MS (four-times smoothed). The over 5500 MS and 4000 MS/MS spectra were manually interpreted on the basis of the masses of the predicted component monosaccharides, differences of mass in glycan series, fragmentation pattern, comparison with co-eluting structures from other nematodes and chemical or exoglycosidase treatments. Two selected 2D-HPLC-purified glucuronylated N-glycans were subject to LC–MS^*n*^ as previously described using a 5 µm porous graphitised carbon column and a LTQ ion trap mass spectrometer (Thermo Scientific) in negative-ion mode^34^ with the spectral interpretation being performed in comparison to the literature^69^. The symbolic annotations of spectra, chromatograms or molecular models are according to the standard nomenclature^70,71^; tables of theoretical *m*/*z* values for relevant compositions are presented in the Supplementary Tables 1 and 2.

### Enzymatic and chemical treatments

Glycans were treated, prior to re-analysis by MALDI-TOF–MS, with α-fucosidase (bovine kidney from Sigma-Aldrich), α-mannosidase (jack bean from Sigma), β-glucuronidases (*E. coli* from Megazyme or *Helix pomatia* from Sigma; desalted and concentrated ten-fold with a centrifugal device with a 10 kDa molecular weight cut-off before use) or β-*N*-acetylhexosaminidases (jack bean from Sigma-Aldrich, *Xanthomonas manihotis* from New England Biolabs, *Streptomyces plicatus* chitinase from New England Biolabs or in-house-produced recombinant forms of *Caenorhabditis elegans* HEX-4 specific for β1,4-GalNAc-linked residues or *Apis mellifera* FDL specific for the β1,2-linked product of GlcNAc-transferase I^19^) in 50 mM ammonium acetate, pH 5, at 37 °C overnight (except for pH 6.5 in the case of HEX-4, or pH 7 in the case of *E. coli* β-glucuronidase or an incubation time of only 3 h in the case of FDL or <2 h for *H. pomatia* β-glucuronidase). Hydrofluoric acid was used for removal of core or antennal α1,3-fucose or of phosphorylcholine^17^. As appropriate, treated glycans were re-chromatographed by RP-amide HPLC to ascertain retention time shifts prior to MALDI-TOF–MS. See also Supplementary Note 2 for discussion of glycosidase specificities and Supplementary Figure 23 for the HEX-4 and chitinase sensitivity of defined disaccharide conjugates.

### Lectin production and biotinylation

For use in western blotting and fluorescence microscopy, two nematotoxic fungal lectins (CGL3 and CCL2 from *Coprinopsis cinerea*) were expressed and purified as previously described^23,72^. In brief: the lectin cDNAs were amplified and cloned into relevant plasmids and then the proteins were expressed in *Escherichia coli* (strain BL21). The lectins were purified via their hexahistidine tags using an Ni-NTA column (Qiagen), subsequently concentrated and buffer-exchanged to phosphate-buffered saline (PBS) using a 10 kDa cut-off Amicon Ultra-4 centrifugal device (Millipore). Both for use in western blotting and in fluorescence microscopy, purified lectins were biotinylated^73^ with EZ-Link sulfo-NHS-biotin kit (Pierce) according to the manufacturer’s instructions, followed by a desalting step on a PD-10 column (Amersham Biosciences).

### Western blotting

For detection of glycan epitopes, protein extracts of adult worms, both female and male were analysed. The worms were cut into pieces of ~2 mm and homogenised using a Dounce homogeniser in lysis buffer (20 mM MES buffer pH 7, 1% Triton X-100, 0.01% protease inhibitor cocktail from Roche). The homogenate was then sonicated for 3 s, centrifuged at 10,000×*g* for 10 min and the supernatant denatured at 95 °C in 4× sample buffer (60 mM Tris–Cl pH 6.8, 2% SDS, 10% glycerol, 5% β-mercaptoethanol, 0.01% bromophenol blue) prior to SDS-PAGE separation (8% acrylamide, 120 V) for subsequent analysis by either Coomassie staining or blotting on nitrocellulose (GE Healthcare). After overnight blocking of the membrane at 4 °C with 0.5% bovine serum albumin in PBST, lectin or antibody detection was performed using either 10 μg/ml of biotinylated lectins (CGL3 or CCL2) or 1:5000 dilution of the murine IgA TEPC 15 (Sigma, M1421). Subsequently the membrane was incubated with, in the lectin case, 10 μg/ml of Streptavidin coupled with HRP (Vector Laboratories, SA-5004) and in the TEPC 15 case a 1:3000 dilution of HRP coupled anti-mouse IgA (Bethyl, A90-103P). After extensive washing (PBST), horseradish peroxidase activity was detected using ECL Direct™ detection reagent (VWR, RPN2105) and exposure to photographic film.

### Histochemistry

For detection of glycan epitopes in tissues, sections of L3 worms were stained with in-house-produced lectins and antibodies. The sample slides were prepared by washing the worms twice with PBS and then fixing them overnight in 4% formaldehyde. After fixation, the sample was washed twice with PBS and incubated overnight in 30% sucrose. Afterwards, the sucrose was completely removed and the worms were embedded in O.C.T. solution (VWR 361603E) in a mould (Fisher 15-182-501-D) and frozen over dry ice. The blocks were stored at −80 °C until sectioning (Cryostat 2800 Frigocut, Cambridge Instruments GmbH with Feather microtome blades N35). The 8 µm slides were air dried for 30 min before the post-sectioning fixation with 4% formaldehyde for 20 min at room temperature. Subsequently, the slides were washed with PBS and blocked in 0.1 M glycine in PBS for 5 min at room temperature and then incubated in 2% BSA in PBS overnight at 4 °C. The slides were then probed either with 10 μg/ml biotinylated lectins (CGL3 or CCL2), or a 1:200 dilution of the monoclonal antibody (TEPC 15) for 1 h at room temperature. After washing with PBS, the slides were incubated with either 15 μg/ml Atto 655 Streptavidin (Sigma-Aldrich, 02744; fluorescently labelled for detection of lectins) or with FITC-conjugated polyclonal goat IgG against mouse immunoglobulins IgG, IgA and IgM (Cappel, 55499; for detection of immunoglobulins) for 1 h at 4 °C in the dark. Finally, the slides were washed three times with PBS and embedded using Vectashield embedding solution containing DAPO (Vector Labs, Burlingame, CA) prior to microscopy with a Leica confocal laser scanning TCS SP8 microscope at the appropriate settings.

### Synthesis of di- and tri-saccharides on an aminoxy-based linker for glycan arraying

*N,N*′-diacetylchitobiose was purchased from Carbosynth and *N,N′-*diacetyllactosediamine (LacdiNAc) synthesised as detailed in the Supplementary Methods (see also Supplementary Figures 24 and 25). The corresponding 6-(5-aminopentanamido)-*N*-(2-[2-[disaccharyl-*N*-methoxyamino]ethoxy)ethyl]-2-naphthamides were prepared as previously described^74^ using the free unprotected disaccharides; these conjugates were also fucosylated with *C. elegans* FUT-6 α1,3-fucosyltransferase and almost quantitative conversion was demonstrated by MALDI-TOF–MS and HPLC (see Supplementary Figure 23).

### Preparation of AEAB-labelled natural glycan pools for glycan arraying

Free N-glycans of the neutral pool were modified reductively with 2-amino-*N*-(2-amino-ethyl)-benzamide (AEAB; excitation/emission of 330/420 nm) as described by Song^75^. An aliquot of the AEAB-labelled glycans was treated for 2 days at 0 °C with hydrofluoric acid to remove core/antennal α1,3-fucose as well as phosphorylcholine. Both the native and treated pools were injected onto a normal phase column (TSKgel Amide-80; reverse gradient of acetonitrile) prior to re-pooling. Fractions were analysed by MALDI-TOF–MS and MS/MS to determine glycan compositions in terms of Hex, HexNAc, Fuc and PC moieties. For both pools and fractions, the integrated HPLC fluorescent peak areas were used to normalise the amounts used for subsequent non-contact printing.

### Glycan array-based lectin and antibody screening

Derivatised glycans were mixed 1:1 with spotting buffer (300 mM sodium phosphate pH 7.5, 0.005% Tween-20) then spotted (*n* = 10; 1–2.5 fmol as described in the supplement) by non-contact printing (Scienion Flexarrayer S1) onto NHS-derivatised Nexterion H glass slides (Schott). After 16 h of hybridisation, slides were blocked (50 mM ethanolamine in 50 mM sodium borate, pH 9.0) for 1 h at RT, washed serially with TSM (20 mM Tris, pH 7.4, 150 mM NaCl, 2 mM CaCl_2_, 2 mM MgCl_2_) with 0.05% Tween-20, TSM alone and H_2_O prior to drying^44,74,75^. The slides were incubated with either dilutions of dog sera (one control and two infected), biotinylated fungal CGL3 and CCL2 (see above) or biotinylated forms of wheat germ agglutinin, *Aleuria aurantia* lectin, concanavalin A or tomato lectin (Vector Laboratories; B1025, B1395, B1005 and B1175), murine TEPC 15 IgA monoclonal (Sigma-Aldrich; M1421), recombinant human mannose binding lectin (Biotechne; 9085-MB-050) or natural human C-reactive protein (MPBio; 215231505) followed by the relevant secondary and/or tertiary antibodies. In experiments with human C1q (Sigma; C1740), slides were incubated first with sera or with C-reactive protein in the absence or presence of additional CaCl_2_ or EDTA, prior to serial application of C1q and fluorescent anti-C1q (Bioss Antibodies; bs-10750R-A647). Slides were scanned with an Agilent G2565CA Microarray Scanner (multiple photomultiplier tube (PMT) gain values from 10 to 100%) and raw image files were analysed by GenePix software. The fluorescence values (green for FITC and red for AlexaFluor-647) were used to calculate (in Excel) the mean and standard deviation from all ten spots. The negative controls (spotting buffer or no primary reagent) show fluorescence due to either the labels themselves or non-specific binding of the fluorescent secondary antibodies. For further details regarding conditions, buffers and dilutions, refer to Supplementary Note 3.

### Reporting summary

Further information on experimental design is available in the Nature Research Reporting Summary linked to this article.

## Supplementary information

Supplementary Information

Reporting Summary

## Data Availability

The data supporting the findings of this study are available within the paper and its Supplementary Information files or from the corresponding author upon request. In addition, MS/MS data underlying Figs. 2–8 and Supplementary Figures 5–15 are available as mzxml files (in the case of MALDI-TOF–MS/MS) or as an excel file (in the case of LC–MS/MS) on Figshare (doi: 10.6084/m9.figshare.7387496). A reporting summary for this Article is available as a Supplementary Information file.
