# Supplementary material for: Highly modified and immunoactive N-glycans of the canine heartworm
Source: Nat Commun. 2019 Jan 8;10:75. doi: 10.1038/s41467-018-07948-7 (PMC6325117; doi:10.1038/s41467-018-07948-7)
Supplement: Supplementary file 1 — Supplementary Information [file 41467_2018_7948_MOESM1_ESM.pdf]

Highly modified and immunoactive N-glycans of the canine heartworm

Francesca Martini, Barbara Eckmair, Saša Štefanić, Chunsheng Jin, Monika Garg, Shi Yan, Carmen Jiménez-Castells, Alba Hykollari, Christine Neupert, Luigi Venco, Daniel Varón Silva, Iain B. H. Wilson and Katharina Paschinger

Supplementary Notes, Figures, Tables, Methods and References

Contents

Supplementary Note 1: Further explanations for some glycomic and array data interpretations	*S2*
Supplementary Note 2: Further information regarding the glycomic analyses	*S4*
Supplementary Note 3: Further information regarding the glycan array analyses	*S7*
Glycomic Workflow (incl. Supp Fig 1)	*S10*
Supplementary Figures 2-22	*S11*
Supplementary Tables 1-3	*S33*
Supplementary Methods: Mass spectrometric analyses of proteins in the parasite extract:	*S36*
Supplementary Methods: Fucosylation of chitobiose or LacdiNAc (incl. Supp Fig 23)	*S37*
Supplementary Methods: Synthesis of LacdiNAc (incl. Supp Fig 24 and 25)	*S38*
Supplementary References	*S42*

Supplementary Note 1

Further explanations for some glycomic and array data interpretations

Regarding the pauci- and oligomannosidic glycans:

The permethylation data shows that the most abundant glycan in the *Dirofilaria* N-glycome is the paucimannosidic $Man_3GlcNAc_2Fuc_1$ structure (MMF^6 in the Schachter nomenclature) followed by $Man_{3/5}GlcNAc_2$. In the off-line LC-MS approach (see **Figure 1** in the main text and **Supplementary Figure 4** for HIAX-HPLC and RP-HPLC chromatograms), $Man_3GlcNAc_2Fuc_1$ and $Man_{3/5}GlcNAc_2$ (*m/z* 1135, eluting at 9 g.u. and *m/z* 989/1313, eluting at 7.2 g.u. by RP-HPLC) have the same elution properties as the 'normal' invertebrate isomers. The off-line approach could also resolve two isomeric hybrid/pseudohybrid species ($Man_3GlcNAc_3Fuc_1$; *m/z* 1338, MGn and GnM in the Schachter nomenclature, eluting at either 8.6 or 11.8 g.u., with respectively the non-reducing GlcNAc on the α1,3- or α1,6-mannose) as well as a range of oligomannosidic glycans ($Man_{6-9}GlcNAc_2$; Figure 1). There was also a trace of lower molecular weight difucosylated N-glycans ($Man_3GlcNAc_2Fuc_2$, *m/z* 1281, and two isomers of $Man_3GlcNAc_3Fuc_2$, *m/z* 1484), which display a hallmark *m/z* 592 ($Fuc_2GlcNAc_1$-PA) MS/MS Y fragment indicative of some degree of core α1,3/6-difucosylation as found also in various invertebrates (**Supplementary Figure 5 B and C**). There were apparently two forms of $Hex_4HexNAc_2Fuc_1$ (*m/z* 1297), one of which yielded an MS/MS fragment of *m/z* 608 and had an elution time (as compared to other nematodes such as *Haemonchus contortus*) suggestive of galactosylation of the core α1,6-fucose residue, but the amounts of this glycan were too low for further analysis (**Supplementary Figure 5E**).

Regarding MS and MS/MS analysis of selected bi-, tri- and tetra-antennary N-glycans:

Isomers of 1946 Da (i.e., *m/z* 1947; $Hex_3HexNAc_6Fuc_1$-PA) were present in the same female HIAX fraction but eluted on the RP-amide column at either 8.3 or 9.8 g.u. (Figure 1, chromatograms D); also in a male HIAX fraction of similar retention time, three isomers eluted from the RP-amide column at 8.3, 8.5 and 12.0 g.u. (Figure 1, chromatogram I). All these glycans possessed *m/z* 446 Y1 fragments ($GlcNAc_1$ Fuc_1-PA; see **Supplementary Figures 5 S/T and 6**) in positive mode MS/MS indicative of a core fucose residue. Corresponding non-fucosylated *m/z* 1801 glycans were also present ($Hex_3HexNAc_6$-PA; lacking the *m/z* 446 fragment upon MS/MS). The tendency (i) of the tetra-antennary *m/z* 1947 glycan to elute between contrasting tri-antennary *m/z* 1744 isomers (see Figure 1, chromatogram C) and (ii) of LacdiNAc-modified or core α1,6-fucosylated glycans to elute relatively late on RP-HPLC was also observed by Tomiya *et al.* in their various studies on PA-labelled glycans *[1]*. Further digestions with specific hexosaminidases (**Supplementary Figure 6**) were used to verify the annotations with either terminal GlcNAc or terminal GalNAc residues. In comparison a glycan of *m/z* 1801, i.e., the non-fucosylated form of *m/z* 1947, has an MS/MS spectrum with the major fragment ions shifted by 146 Da (i.e., Δ = Fuc; **Supplementary Figure 7**).

Regarding the elution and digestion properties of glucuronylated glycans:

Due to the ability to detect anionic N-glycans in the negative ion mode and digest them with *Helix pomatia* β-glucuronidase, the relevant 176 Da increment on these structures, as compared to some neutral structures, was concluded to be a hexuronic acid rather than a methylhexose. Glucuronylated glycans also elute earlier on RP-amide, but later on the HIAX column as compared to related neutral structures (see **Figure 8** in the main text). Furthermore, glycans carrying lower arm glucuronic acid residues on LacdiNAc ('short' arm) elute earlier than the corresponding upper arm isomer (see **Figure 5 A/B**, **Supplementary Figure 12** A/C and E/G, **Supplementary Figure 13A** and **Supplementary Figure 14 B/D**), whereas glycans carrying glucuronic acid on a $GalNAcGlcNAc_2$ motif ('long arm') elute even later (**Figure 5B**, **Supplementary Figures 13A and 14F**); on the other hand, the presence of single β1,2/β1,6-GlcNAc residues on the upper arm resulted in earlier

elution (**Supplementary Figure 14A**). As shown in **Supplementary Figure 13 A/B**, loss of antennal GalNAc upon HEX-4 treatment results in forward shifts in retention time, whereas the loss of fucose or phosphorylcholine upon hydrofluoric acid treatment generally, but not always, causes a backward shift. When digesting glycans with the *Helix pomatia* β-glucuronidase, which also contains a contaminating α-fucosidase activity, there is a major forward RP-amide HPLC shift due to loss of α1,6-fucose; in contrast, the loss of one glucuronic acid from a 'long' upper arm has a small effect (backward) on the retention time, while loss of the glucuronic acid from a 'short' lower arm or of both glucuronic acid residues had a greater impact on elution (see also **Figure 8**). Note that *E. coli* β-glucuronidase (able to remove β1,3-linked glucuronic acid from insect N-glycans) was inactive towards *D. immitis* glycans, indicative of a structural difference, which was corroborated by LC-MS^n data showing a β1,4-linkage. However, *H. pomatia* β-glucuronidase is rather impure in terms of contaminants which interfere with MALDI-TOF MS analysis as well as containing other glycosidases, thus care had to be taken with the incubation time and the digests were generally re-chromatographed.

Regarding lectin binding to the N-glycan pool and di/tri-saccharide arrays:
(i) The high binding of WGA to chitobiose and LacdiNAc (**Supplementary Figure 18D**) is in keeping with literature data, while the reduction in WGA binding to the fucosylated forms of chitobiose and LacdiNAc as compared to the non-fucosylated disaccharides correlates with the increase in binding to the N-glycans when antennal fucose residues are removed upon HF treatment (**Supplementary Figure 18C**).
(ii) AAL binds to native *Dirofilaria* N-glycans (**Supplementary Figure 18C**) with, as judged by analysis of binding to HPLC fractions (see **Supplementary Figure 19D**), a bias towards binding of smaller core α1,6-fucosylated N-glycans as opposed to the larger structures.
(iii) LEL may bind the longer chito-oligomer chains after HF treatment (**Supplementary Figure 18C**), which may correlate with haemagglutination inhibition data with chito-oligosaccharides and its known binding to poly-*N*-acetyllactosamine repeats.
(iv) Binding of glycans to MBL is, as expected, Ca^{2+}-dependent and is increased towards the HF-treated glycans (**Supplementary Figure 18C**); potentially some 'HF-stripped' longer chains are also sensitive to Endo H or the presentation of the glycome pool to the multimeric lectin is altered, thereby accounting for the decrease in binding upon use of the endoglycosidase, which is also seen when analyzing the interaction of MBL to size-fractionated pools of glycans (see **Supplementary Figure 21A**).
(v) CGL3 binds LacdiNAc and its interaction with N-glycans (about one-tenth of that to the other lectins; see **Figure 9D** in the main text) increases somewhat after HF treatment, whereas CCL2 binds synthetic fucosylated LacdiNAc (**Supplementary Figure 18D**), but interaction with core α1,3-fucosylated glycans from *Dirofilaria* is prevented by the 'open ring' conformation of the proximal GlcNAc (a result of the reductive procedure during AEAB labelling); the presentation, conformation and concentration of glycoproteins on a blot (see **Supplementary Figure 16B**) will certainly differ from those of the glycan pools on the array.

Supplementary Note 2
Further information regarding the glycomic analyses

Search parameters and acceptance criteria

a. **Peak lists:** As stated in the methods section: typically 1000 shots were summed for MS and 5000 for MS/MS. Spectra were processed with the manufacturer's software (Bruker Flexanalysis 3.3.80) using the SNAP algorithm with a signal/noise threshold of 6 for MS (unsmoothed) and 3 for MS/MS (four-times smoothed).
b. **Search engine, database and fixed modifications:** All glycan data were manually interpreted and no search engine or database was employed; the fixed modification is the pyridylamine label at the reducing end ($GlcNAc_1$-PA fragments of *m/z* 300).
c. **Exclusion of known contaminants and threshold:** All glycan data were manually interpreted; only peaks with an MS/MS consistent with a pyridylaminated chitobiose core were included – the 'threshold' for inclusion was an interpretable MS/MS spectrum (at least in terms of composition).
d. **Enzyme specificity:** A description of the release methods (PNGase F followed by PNGase A) is given in the methods section. Enzymes used during the analysis (glycosyl hydrolases) are defined in the methods by species name and supplier. Citations for in-house purified recombinant enzymes are also given. As previous experience with normalizing glycosidase amounts based on units of activity towards *p*-nitrophenyl sugars reduced digestion efficiency towards native oligosaccharides, aliquots of glycans (equivalent to 5 – 50 mV in terms of fluorescence) were incubated with 0.2 µl of the various enzyme preparations (whether commercial, desalted commercial or in-house produced) overnight (except for three hours in the case of insect FDL digests or shorter times in the case of *Helix pomatia* β-glucuronidase). These conditions result (other than in the case of the glucuronidase) in no obvious unspecific removal of residues as defined by shifts in mass, MS/MS or retention times, although steric hindrance in some glycans leads to a requirement for longer incubation times (48 hours). Ammonium acetate buffers were used (supplemented with $CaCl_2$ where required) as suppliers' buffers interfere with MALDI-TOF MS analysis; generally one-quarter of any glycosidase digest was applied directly to the target plate prior to drying and addition of matrix. Hydroflouric acid treatment (3 µl of 48% HF added to the dried glycan) was 24 or 48 hours on ice in the cold room prior to drying under vacuum; expected release of α1,3-fucose and phosphodiester residues, but not of other sugars, was observed under these conditions.
   Fucosidases: bovine α-fucosidase (Sigma, native) removes core α1,6-fucose, but not core α1,3-fucose; almond α1,3/4-fucosidase (Prozyme, native) removes antennal α1,3-fucose from the subterminal GlcNAc of LacdiNAc/LacNAc motifs, active towards Lewis X epitopes in a parallel study, but apparently neither defucosylates chitobiose antennae nor a terminal GalNAc of a LacdiNAc motif – thus, hydrofluoric acid was more effective for the fucose removal in this study. α1,2-Fucosidases were not employed in this study.
   Glucuronidases: *E. coli* β-glucuronidase (Megazyme) removes β1,3-linked glucuronic acid from galactose residues (e.g., as found in insects), but apparently not glucuronic acid β1,4-linked to GalNAc as found in *Dirofilaria*; *H. pomatia* β-glucuronidase (Sigma, native) removes glucuronic acid from galactose or GalNAc residues, but also contains hexosaminidase and core fucosidase activities – thus only short incubation times show specificity. Both glucuronidases were desalted before use.
   Hexosaminidases: Jack bean β-*N*-acetylhexosaminidase (Sigma, native) is a general-purpose enzyme unspecifically removing β-linked GlcNAc and GalNAc residues; *Xanthomonas* β1,2-*N*-acetylglucosaminidase (NEB, no longer sold, recombinantly-

expressed in *E. coli*) is a Ca^{2+}-dependent enzyme which apparently only removes β1,2-linked GlcNAc residues, but may be sterically hindered by the β1,4-GlcNAc antenna; *Streptomyces* β1,3/4-*N*-acetylhexosaminidase (NEB, 'chitinase' recombinantly-expressed in *E. coli*) removes β1,4-linked GalNAc from LacdiNAc and β1,4-linked GlcNAc from chitobiose motifs and recent lots do not remove the β1,4-linked GlcNAc attached directly to α1,3-mannose in tri- and tetraantennary glycans; *C. elegans* HEX-4 is an in-house prepared enzyme (His-tag purified recombinant form expressed in *Pichia*) which demonstrably removes β1,4-linked GalNAc residues, but in this and previous studies is not observed to remove any other HexNAc residue (see also **Supplementary Figure 23**); insect FDL is also prepared in-house (His-tag purified recombinant form expressed in *Pichia*) and under the stated conditions (3 hour incubation) only removes the β1,2-linked GlcNAc attached directly to α1,3-mannose in Man3-based hybrid and biantennary glycans, in keeping with its proven role in the *in vivo* generation of paucimannosidic glycans.

Mannosidases: Jack bean α-mannosidase (Sigma or Prozyme, native) removes all α-mannose residues, but steric hindrance slows its action, e.g., digestion of core α1,6-mannose is reduced if there is a 'lower' arm modification on the core α1,3-mannose; more specific α1,2-, α1,2/3- or α1,6-mannosidases were not used in this study.

e. **Isobaric/isomeric assignments:** For isomeric species, 2D-HPLC elution, differences in MS/MS and/or digestion data were used for the assignment (as described in the text).

Glycan or glycoconjugate identification

a. **Precursor charge and mass/charge (*m/z*):** All glycans detected were singly-charged. For the positive mode, the *m/z* values are for protonated forms, whereas in negative mode the ions are $[M-H]^-$. Depending on the glycan amount or presence of buffers in exoglycosidase preparations, the relative amounts of the H^+ and Na^+ adducts varied. Maximally two decimal places used for the *m/z* annotations consistent with the accuracy of MALDI-TOF MS; in the figures and due to space limitations, only one decimal place is presented. Previous data indicate an average +0.03 Da (+ 22 ppm) deviation between the measured and the calculated *m/z* values on the instrument used.
b. **MALDI-TOF MS settings (positive and negative modes):** Ion Source 1 and 2 were 19.00 and 16.75 kV; Lens, 9.00 kV (7.95 kV for over 3000 Da or for negative mode); Reflector 1 and 2, 21.05 and 9.65 kV; Pulsed Ion Extraction, 160 ns (120 ns in negative mode); Matrix Suppression typically up to 700 Da; Detector Gain, typically 2163 V.
c. **MALDI-TOF MS/MS settings (positive and negative modes):** Ion Source 1 and 2 were 6.00 and 5.35 kV; Lens, 2.90 kV; Reflector 1 and 2, 27.00 and 11.75 kV; Lift 1 and 2, 19.00 and 4.00 kV; Pulsed Ion Extraction, 140 ns; Detector Gain, typically 2260 V when fragmenting; Laser Power Boost typically 50%; not in CID mode; PCIS typically 0.65%.
d. **All assignments:** For the glycans present in each pool, see the HIAX and RP-amide-HPLC chromatograms annotated with structures shown according to the Standard Nomenclature for Glycans. Downwardly- and upwardly-drawn core fucose and mannose residues are respectively α1,3- and α1,6-linked (see **Figure 1**, inset, in the main text) .
e. **Modifications observed:** Listed are the *m/z* values for glycans carrying a reducing terminal pyridylamine group as judged by presence of an *m/z* 300 $GlcNAc_1$-PA fragment. As the glycans are otherwise chemically unmodified, *Δm/z* of 146, 162, 165 and 203 correspond to deoxyhexose (presumed to be fucose), hexose, phosphorylcholine or *N*-acetylhexosamine. As glycans with *Δm/z* of 176 in this study were strongly detected in the negative mode and sensitive to glucuronidase, this mass difference was considered to correspond to hexuronic acid (presumed to be glucuronic acid) and not to methylhexose.

f. **Number of assigned masses:** Glycan assignments were not just based on measured mass only, but on at least MS/MS, in most cases corroborated by digest and elution data. The linear mass spectra of higher molecular weight glycans are shown to indicate their presence but not their structure.
g. **Spectra:** Representative annotated spectra (MS and MS/MS) defining structural elements are given in various figures. In total, MS and/or MS/MS data for some 80 of the approximately 150 defined structures are shown; no structures for glycans of more than 4000 Da are proposed, but example spectra of these are also included in the Supplement. The overall data is based on some 10000 MS and MS/MS spectra.
h. **Structural assignments:** As noted in the results section, the typical oligomannosidic structures are assigned based on elution time and fragmentation pattern; it is otherwise assumed that the glycans contain a trimannosyl core consistent with typical eukaryotic N-glycan biosynthesis. The presence of GlcNAcTIV (MGAT4) and GlcNAcTV (MGAT5) homologues in *D. immitis* are compatible with the proposed tri- and tetra-antennary glycans, whose fragmentation patterns show preferential loss of the 'heaviest' antenna. However, due to the low abundance of some glycans or occasional steric hindrance, an absolute assignment of the elongations on the four antennae was not always possible, despite the use of *Xanthomonas* β-*N*-acetylglucosaminidase as an enzyme specific for the β1,2-linked GlcNAc.
The assignments of antennal and core fucose residues are based on RP-HPLC retention time, fragmentation pattern and/or susceptibility to digestions. Other antennal modifications (phosphorylcholine, glucuronic acid and *N*-acetylgalactosamine; including anomericity of the glycosidic linkage) are defined based on digestions and fragmentation patterns with rechromatography after digestion in some cases. Two example structures modified with glucuronic acid were also analysed by LC-ESI-MS^n.
There is no evidence of in-source fragmentation of either neutral terminal monosaccharides (including Lewis-type fucosylation) or phosphorylcholine (as evidenced by this and previous publications); the degree of in-source fragmentation of multiply-glucuronylated glycans depends on the laser power used, but is not significant in the most cases (see negative mode spectra in Figure 8 of the main text).

Summary of glycosidases used in this study

PNGases	*Flavobacterium* PNGase F (recombinant, *E. coli*)<br>Almond PNGase A (native)
Fucosidases	Bovine α-fucosidase (native)<br>Almond α1,3/4-fucosidase (native)
Glucuronidases	*E. coli* β-glucuronidase (recombinant, *E. coli*)<br>*H. pomatia* β-glucuronidase (native)
Hexosaminidases	Jack bean β-*N*-acetylhexosaminidase (native)<br>*Xanthomonas* β1,2-*N*-acetylglucosaminidase (recombinant, *E. coli*)<br>*Streptomyces* β1,3/4-*N*-acetylhexosaminidase (recombinant, *E. coli*)<br>*Caenorhabditis* HEX-4 β1,2-*N*-acetylgalactosaminidase (recombinant, *Pichia*)<br>Honeybee FDL β1,2-*N*-acetylglucosaminidase (recombinant, *Pichia*)
Mannosidases	Jack bean α-mannosidase (native)

Supplementary Note 3
Further information regarding the glycan array analyses

**1. Glycan Binding Samples**	
Description of Sample	Fungal/plant lectins: biotinylated forms of CGL3/CCL2 from *Coprinopsis* recombinantly expressed in *E. coli* (produced in house), wheat germ agglutinin, concanavalin A, *Aleuria* and tomato lectins (Vector Laboratories, B1025, B1005, B1395 and B1175); used at 20 µg/ml.<br>Murine monoclonal antibody: TEPC-15 (Sigma, M1421); diluted 1:200.<br>Human lectin or pentraxin: Mannose binding lectin (Biotechne; recombinant, 9085-MB-050) or C-reactive protein (MPBio; natural, 215231505), both used at 10 µg/ml.<br>Human complement component: C1q (Sigma, C1740; natural, for indirect assay of glycan-dependent binding) used at 10 µg/ml.<br>Control serum was from an uninfected dog; the two sera of dogs naturally-infected with microfilariae (BEI Resources, cat. no. NR-48908) were centrifuged to remove the parasites; sera were diluted 1:250.<br>All dilutions were in TSM (20 mM Tris-HCl, pH 7.4, 150 mM NaCl, 2 mM $CaCl_2$, 2 mM $MgCl_2$) supplemented with 0.05 % Tween-20, 1% bovine serum albumin (hereafter TSMTB).
Assay protocol	The slides were incubated (all dilutions in TSMTB) with either:<br>(i) Biotinylated forms of recombinant or commercial lectins (20 µg/ml) followed by anti-biotin FITC conjugate (Sigma, F4024; diluted 1:80).<br>(ii) TEPC-15 (Sigma, C1740; 1:200) followed by goat anti-mouse IgA AlexaFluor-647 conjugate (Invitrogen, M31101; 1:1000).<br>(iii) Human mannose binding protein (MBL; Biotechne, 9085-MB-050; 10 µg/ml) followed by mouse anti-MBL (abcam, ab23457; 1:1000).<br>(iv) Dog sera (1:250) followed by rabbit anti-dog IgM or anti-dog IgG (Sigma, SAB3700127 and D7407; 1:1000, i.e., 1 µg/ml) and finally anti-rabbit IgG AlexaFluor-647 conjugate (Invitrogen, A21239; 1:1000).<br>(v) Human C-reactive protein (CRP; MPBio, 215231505; 10 µg/ml) followed by anti-CRP IgG from rabbit (Dako, A0073; 1:1000) and finally anti-rabbit IgG AlexaFluor-647 conjugate (Invitrogen, A21239; 1:1000).<br>(vi) Alternatively, CRP followed by human C1q (Sigma, C1740; 10 µg/ml) and finally rabbit anti-C1q IgG AlexaFluor-647 conjugate (Bioss Antibodies, bs-10750R-A647; 1:1000, i.e., 1 µg/ml); 5 mM $CaCl_2$ or EDTA were added to the buffers.<br>(vii) Dog sera (1:250) followed by incubation with human C1q (10 µg/ml in TSMTB) and then rabbit anti-C1q IgG AlexaFluor-647 conjugate (Bioss Antibodies, bs-10750R-A647; 1:1000, i.e., 1 µg/ml); 5 mM $CaCl_2$ or EDTA were added to the buffers.<br>(viii) Dog sera (1:250) followed by incubation with rabbit anti-human CRP IgG (Dako, A0073; 1:1000) and finally anti-rabbit IgG AlexaFluor-647 conjugate (Invitrogen, A21239; 1:1000).

Incubation and washing	Each incubation step was one hour; washing was by dipping ten times in TSMT, then ten times in TSM and then water, followed by drying.
Sample modifications	Fluorescent antibodies (conjugated with either AlexaFluor-647 or FITC) were used which detect the relevant primary antibodies or which were specific for biotin in order to detect binding of biotinylated lectins.
**2. Glycan Library**	
Glycan description for defined glycans	Refer to Figure 10 and Supplementary Figure 23 for SNFG-style structures of the chemically and chemoenzymatically prepared 6-(5-aminopentan-amido)-*N*-(2-[2-[oligosaccharyl-*N*-methoxyamino]-ethoxy)ethyl]-2-naphthamides (non-fucosylated and fucosylated forms of *N,N′*-diacetylchitobiose and *N,N'*-diacetyllactosediamine); see the supplement for further details regarding synthesis.
Glycan description for undefined glycans	N-glycans were prepared by PNGaseF/A release of glycopeptides derived by thermolysin proteolysis of *Dirofilaria immitis* male and female adult homogenates; see the experimental procedures for further details regarding isolation, AEAB-labelling, hydrofluoric acid treatment and HPLC as well as Figure 9 and Supplementary Figure 19 for MALDI-TOF and HPLC profiles. A detailed analysis of pyridylaminated *Dirofilaria* N-glycans is also presented. Normalisation was on the basis of fluorescence intensity of HPLC peaks in comparison to dilutions of a 0.085 mg/ml (86 µM) AEAB-labelled maltopentaose standard (M_r 991).
Glycan modifications	(i) *N,N′*-diacetylchitobiose and *N,N'*-diacetyllactosediamine were derivatised to form 6-(5-aminopentan-amido)-N-(2-[2-[disaccharyl-*N*-methoxyamino]ethoxy)ethyl]-2-naphthamides via methyoxyamine conjugation (closed-ring); aliquots were then enzymatically fucosylated prior to HPLC purification.<br>(ii) Glycan pools were derivatised with 2-amino-*N*-(2-amino-ethyl)-benzamide (AEAB) by reductive amination (open ring) and the pools were HPLC purified to remove residual linker prior to re-pooling; also individual HPLC fractions were employed. Aliquots of the glycan pools were subject to HF treatment to remove phosphorylcholine and α1,3-fucose residues prior to HPLC. 'On-slide' endoglycosidase H treatment was also performed in some experiments.
**4. Arrayer (Printer)**	
Description of Arrayer	Scienion Flexarrayer S1
Dispensing mechanism	Non-contact printing with the supplier's nozzles (Type 3) and a pulse of 45 µs, ca. 103 V.
Glycan deposition	Ten replicates of 0.8 nl each, except for fractions (either 3 or 8 replicates).<br>For AEAB-labelled natural glycan pools, an estimated 2.5 ± 0.2 fmol per spot was printed as estimated by fluorescence area of HPLC peaks in comparison to a standard (100000 RFU per pmol); for the fractions, an estimated 1 fmol was printed. For 6-(5-aminopentanamido)-*N*-(2-[2-[saccharyl-*N*-methoxyamino]ethoxy)ethyl]-2-naphthamides, a spotting concentration of ca. 1 µM resulted in ca. 1 fmol per spot.

Printing conditions	Derivatised glycans and oligosaccharides were mixed 1:1 with spotting buffer (300 mM sodium phosphate pH 7.5, 0.005% Tween-20); printing was at room temperature; arrays were left to hybridise overnight prior to blocking (50 mM ethanolamine in 50 mM sodium borate, pH 9.0) for 1 h at RT, washing (in TSM + Tween, followed by TSM alone, and finally H_2O) and drying.
**5. Glycan Microarray**	
Array layout	Fourteen subarrays per slide with ten replicates per glycan sample.
Glycan identification and quality control	Glycans on the array were either pools of natural glycans or chemically/chemoenzymatically synthesized oligosaccharides. All glycans were HPLC purified and verified by MALDI-TOF MS.<br>Positive controls for lectin binding (GlcNAcβ1,4GlcNAc and GalNAcβ1,4GalNAc as well as fucosylated forms) were included. Spotting buffer alone was used as one of the negative controls. For selected HPLC fractions, concanavalin A binding was tested.<br>Specificity of TEPC-15 and C-reactive protein binding was tested by removal of phosphorylcholine from N-glycans by hydrofluoric acid (HF), whereafter reduced binding was observed; on the other hand, binding to other lectins which may prefer unmodified N-glycan antennae increased after HF treatment. Binding to MBL was reduced after endoglycosidase H treatment.
**6. Detector and Data Processing**	
Scanning hardware	Agilent G2565CA Microarray Scanner
Scanner settings	Multiple photomultiplier tube (PMT) gain values from 10-100%
Image analysis software	GenePix Pro 7
Data processing	Raw .tif image files were imported to GenePix 7.2.22; feature diameter was set to 100 µm and fluorescent intensities of spots in each sub-array were analysed. Resulting data (F525 or F635 mean values) were exported into Excel without subtraction; significance calculations and t-tests (parametric, unpaired, two-tailed, confidence level 95%) were performed in Excel.
**7. Glycan Microarray Data Presentation**	
Data presentation	Bar charts with individual data points and/or box & whisker plots as shown in Figures 9/10 and Supplementary Figures 18-21. Example raw array images are shown in Figure 9 and Supplementary Figure 19.
**8. Interpretation and Conclusion from Microarray Data**	
Data interpretation	No software used.
Conclusions	As stated in text.

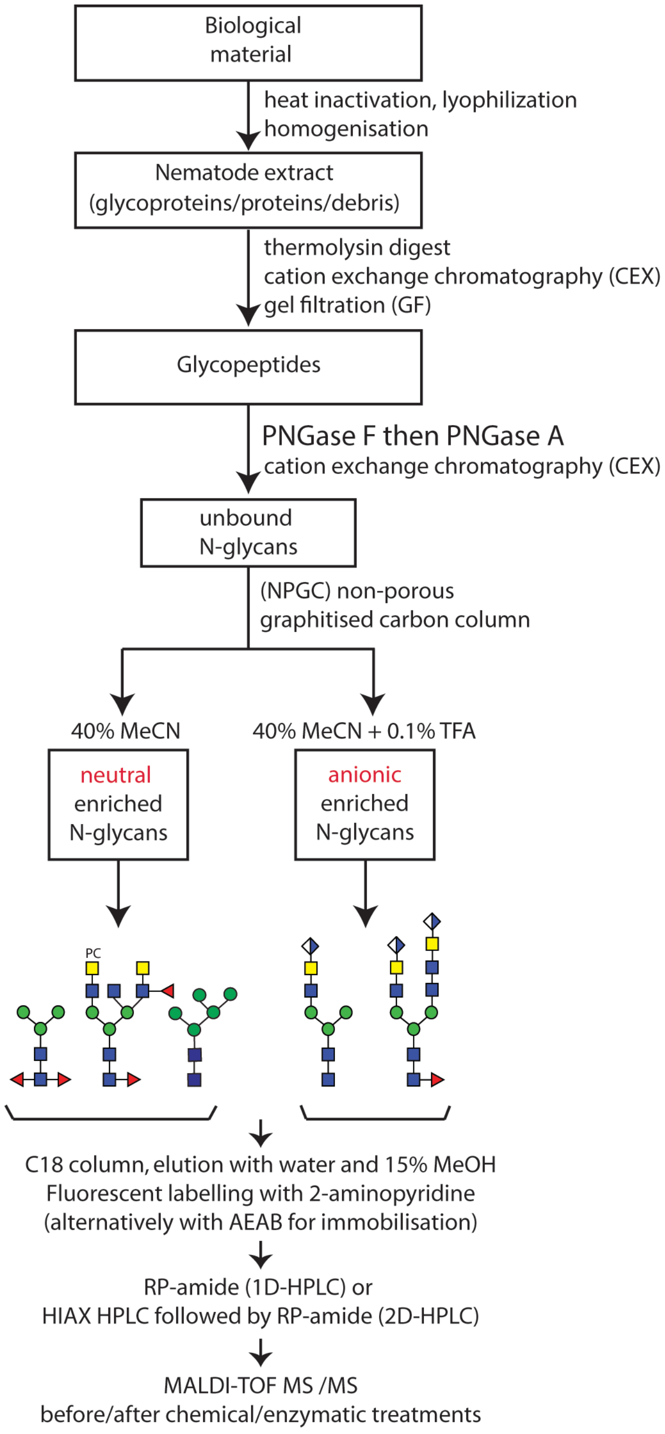

Supplementary Figure 1:

Glycomic workflow employed in this study: Summary of the Experimental Procedures indicating serial digestion with PNGase F then PNGase A followed by solid-phase extraction and labelling steps. Example glycans in two different pools are shown. The pyridylamination procedure, based on that of Hase *[2]*, as well as other details are described as methods protocols by Hykollari *et al*. *[3]*.

Definition of the level of the glycan structural analysis: The goal was the in-depth analysis of the N-glycomes of different samples of *Dirofilaria immitis* adults. Thus individual glycan-containing HPLC fractions were subject to MALDI-TOF MS and MS/MS, a range of chemical and enzymatic treatments and (if appropriate) re-chromatography. Three samples were analysed: a first 'large-worm' (i.e., females) preparation, a 'small-worm' (i.e., males) preparation and a second 'large-worm' (i.e., females) preparation. Neutral and anionic pools were separately pyridylaminated for each sample (see ***Supplementary Figure 2***). Due to preliminary data indicating only low levels of core α1,3-fucosylation, the PNGase F and A digests were performed serially without separation into different pools. Hydrazinolysis of the residual glycopeptides after PNGase F/A release indicated the presence of a low level of remaining N-glycans, but no 'extra' N-glycans with compositions/elution times different from those for the enzymatically-released structures were detected.

Supplementary Figure 2:

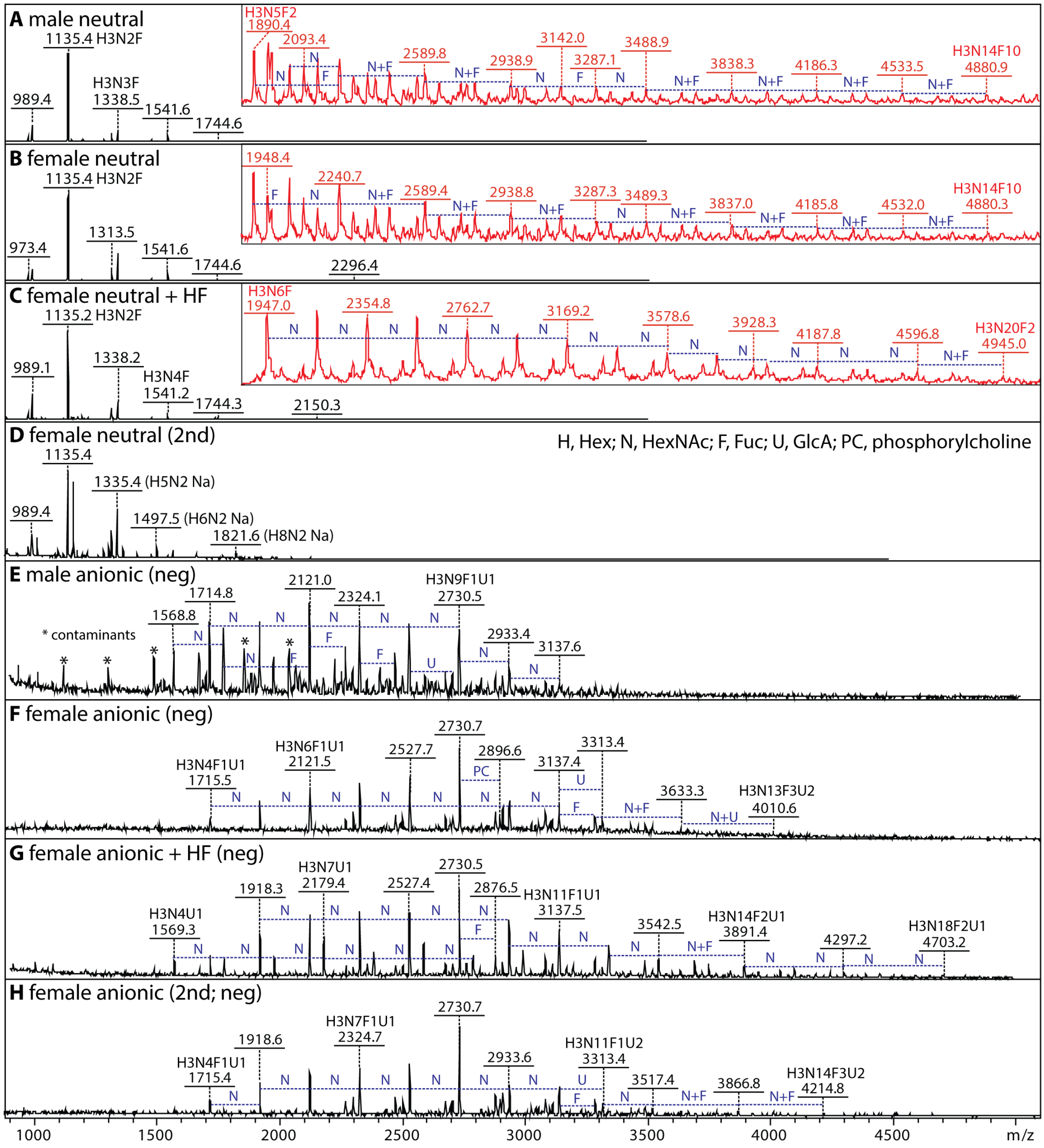

MALDI-TOF MS spectra of complete neutral and anionic pools of *Dirofilaria immitis* pyridylaminated (PA) N-glycans. Positive mode **(A-D)** and negative mode **(E-H)** reflectron spectra (monoisotopic *m/z* values; $[M+H]^+$ or $[M-H]^-$ unless stated) of complete neutral and anionic N-glycomes (male, female before and after HF-treatment as well as of a second female preparation). The insets in red are of linear spectra (with average, not monoisotopic, *m/z* values) of the neutral pools showing the presence of longer HexNAc/Fuc-containing chains, which are primarily converted to long HexNAc-containing chains after removal of α1,3-fucose with hydrofluoric acid; the anionic glycan spectra suggest the presence of a hexuronic acid 'cap' on structures with at least two antennal HexNAc residues.

Supplementary Figure 3:

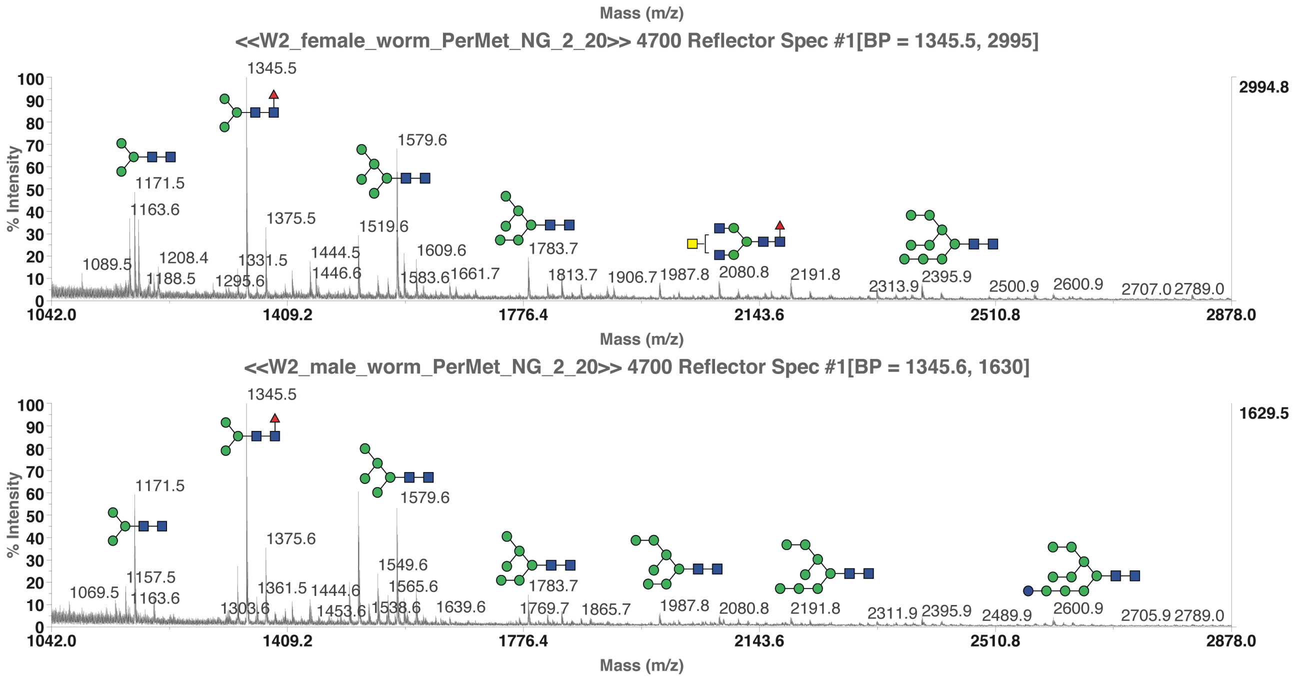

Preliminary analysis of female and male *D. immitis* N-glycans by permethylation. N-glycans were released by PNGase A and permethylated by standard protocols as described by Dell *et al. [4]*, prior to analysis with an Applied Biosystems MALDI-TOF MS; selected $[M+Na]^+$ ions are annotated. Thereby permethylation of male and female glycans neither revealed the full variety of glycans nor the presence of anionic species.

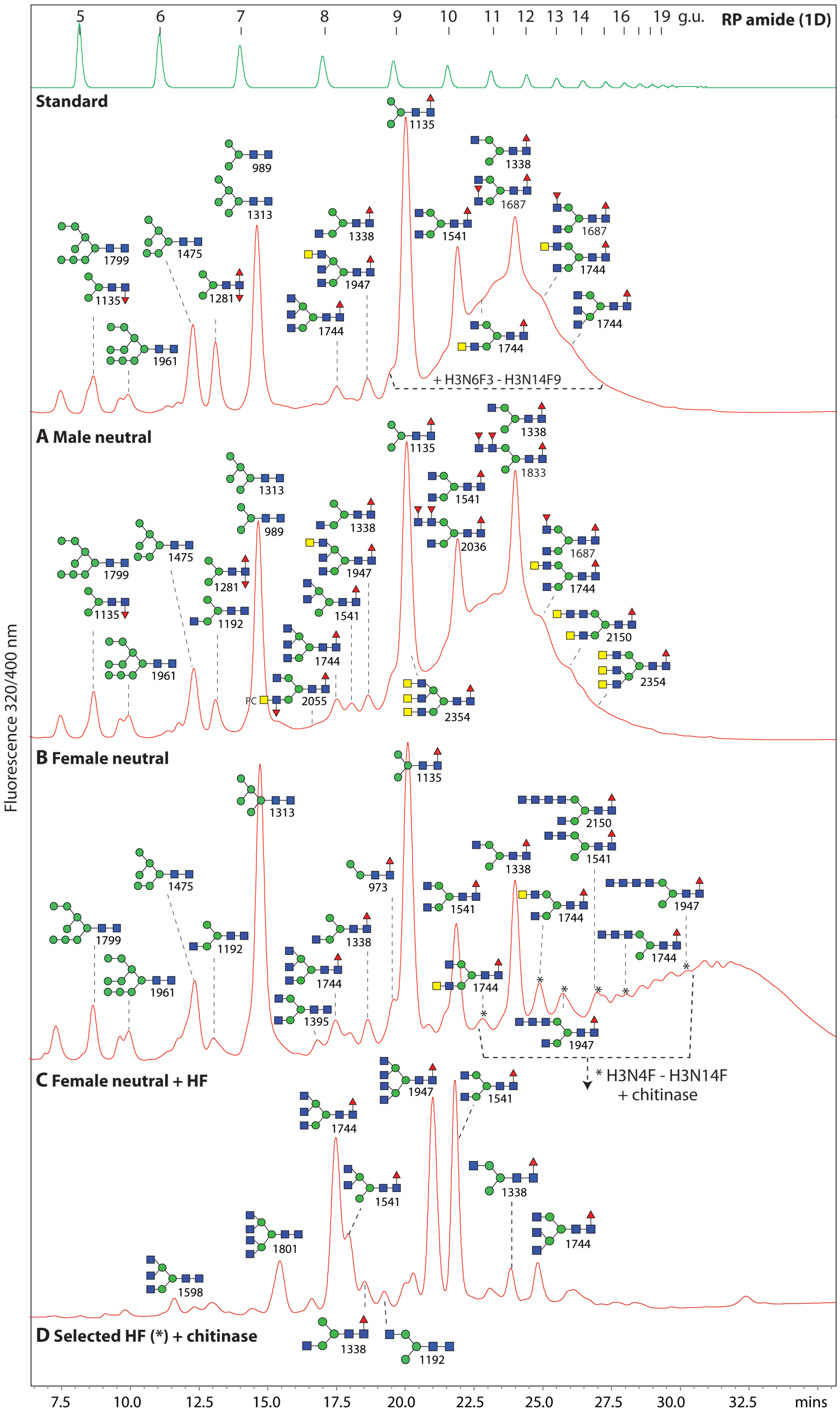

Supplementary Figure 4: RP-amide HPLC chromatograms of complete pyridylamino-labelled neutral pools of *Dirofilaria immitis* N-glycans. (A and B) RP-amide HPLC ('one dimensional') of complete N-neutral glycomes (male and female) with detection by fluorescence (320/400 nm); the pyridylaminated dextran partial hydrolysate standard is also shown, whereby the degree of polymerisation was proven by MALDI-TOF MS of selected calibrant peaks. **(C)** RP-amide HPLC of the female neutral N-glycome after hydrofluoric acid treatment (HF) removing antennal Fuc and PC showing that more resolved peaks occur in the 9-19 g.u. region of the chromatogram and a shift of the unresolved region to higher retention time. **(D)** RP-amide HPLC of selected HF-treated fractions (marked by an asterisk on chromatogram C) incubated with chitinase prior to reinjection. All fractions were subject to MALDI-TOF MS (*m/z* values of major species are given together with the proposed glycan structures). In the case of large glycans with long antennae, the regions in which they elute, as judged by linear mode MALDI-TOF MS, are indicated by compositions of the form H3NxFy; these glycans are digested down to the defined structures (as annotated) by the combined HF/chitinase treatment (panel D) which results in a loss of the unresolved elution 'hills' in the other chromatograms (9-15 g.u. before [A/B] and >11 g.u. after [C] HF treatment). The removal of the antennal α1,3-fucose residues by HF results in a shift to later retention times, whereas the subsequent chitinase digestion yields glycans which co-elute with basic hybrid, mono-, bi, tri- and tetraantennary structures, most of which are core α1,6-fucosylated. Note that, for trianntennary structures, β1,6GlcNAc-antennae on the α1,6Man (upper arm) predominate over β1,4GlcNAc modifications on the α1,3Man (lower arm).

Supplementary Figure 5:

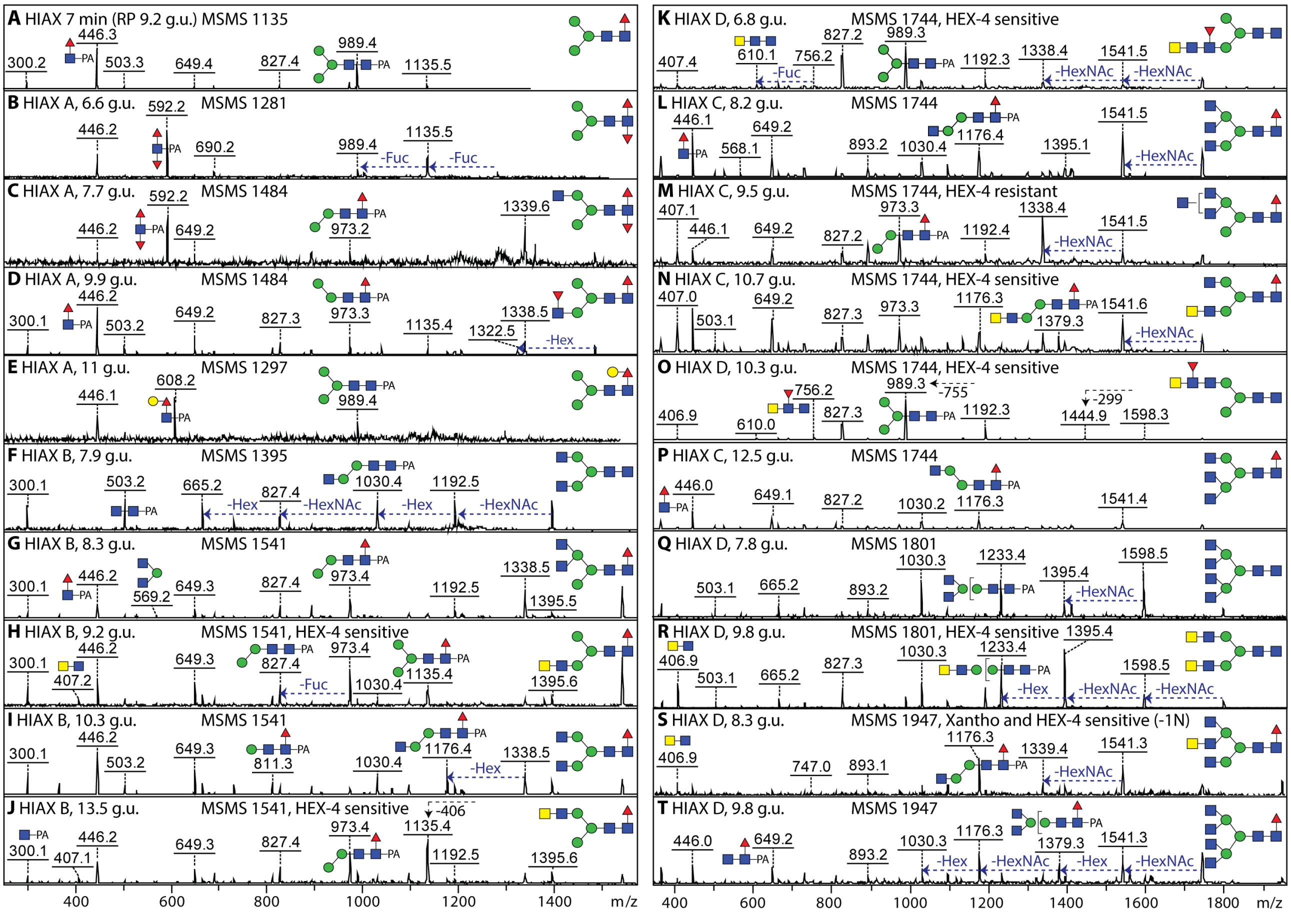

Selected MALDI-TOF MS/MS of neutral *Dirofilaria* 2D-separated N-glycans. N-glycans purified serially by HIAX (for definition of the pools A-D, see Figure 1 of the main text) and RP-amide HPLC (glucose units indicated) were subject to positive mode MALDI-TOF MS/MS of the protonated pseudomolecular ion. MS/MS of **(A-E)** paucimannosidic and hybrid N-glycans ($Hex_{3-4}HexNAc_{2-3}Fuc_{1-2}$), **(F)** a simple biantennary structure, **(G-J)** isomers of $Hex_3HexNAc_4Fuc_1$, **(K-P)** isomers of $Hex_3HexNAc_5Fuc_1$, **(Q and R)** two isomers of $Hex_3HexNAc_6$ and **(S and T)** two isomers of $Hex_3HexNAc_6Fuc_1$ are presented. The isomers existing in the same or neighbouring HIAX pools were separated by RP-HPLC prior to MALDI-TOF MS. Diagnostic patterns of HEX-4 sensitivity or resistance, correlating with the presence or absence of LacdiNAc, are indicated; see also ***Supplementary Figures 6 and 7*** for data on alterations of the fragmentation patterns upon hexosaminidase digestion of selected *m/z* 1801 and 1947 isomers. Key B-fragments (such as $HexNAc_{2-3}$, *m/z* 407 and 610) and Y-fragments (the latter containing the pyridylamino-label [PA] at the reducing end, such as *m/z* 300, 446, 592 or 608 for $GlcNAc_1Fuc_{0-2}$-PA or $GlcNAc_1Fuc_1Gal_1$-PA) are annotated, whereas selected examples of losses of fucose (Fuc), *N*-acetylhexosamine (HexNAc), hexose (Hex) or of pyridylaminated reducing-terminal GlcNAc (-299) are indicated with arrows. The overall structures are shown on the right hand side of each panel. The presented *m/z* 1541 and 1744 isomers are representative of the range of the detected antennal and branching variations in the *D. immitis* N-glycome.

Supplementary Figure 6:

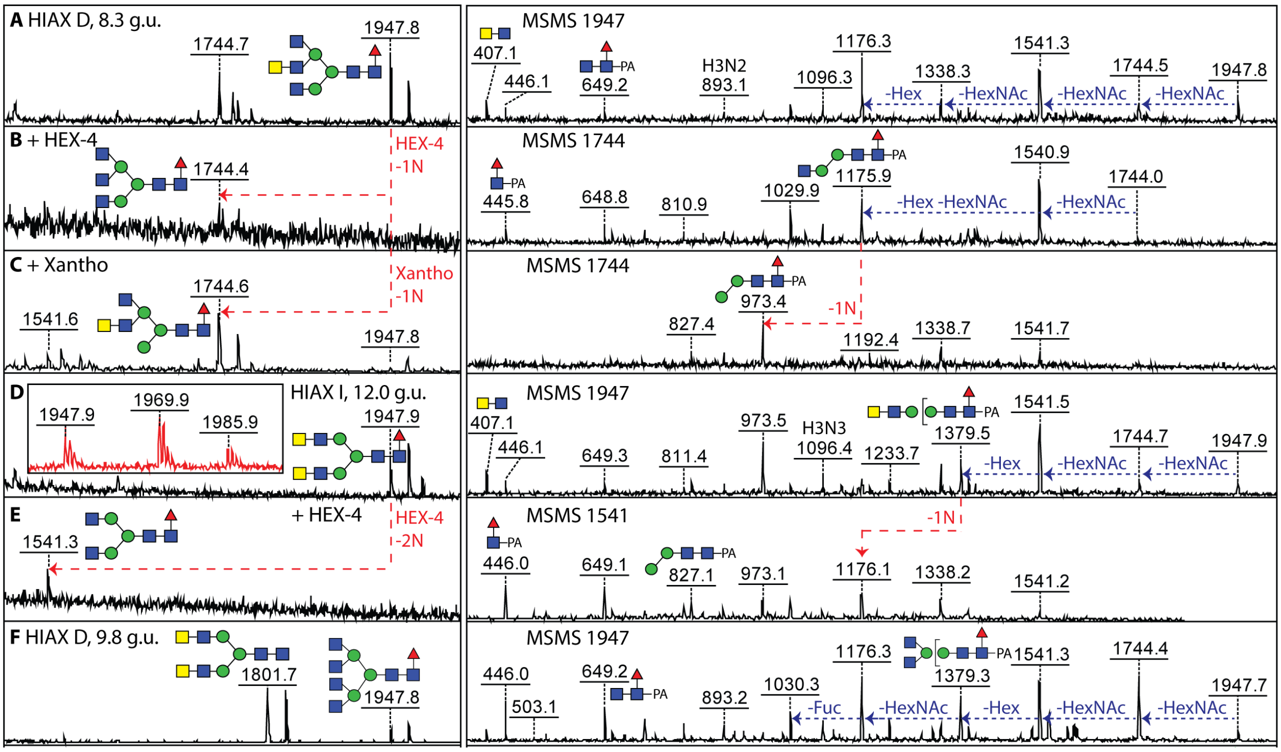

Further MS and MS/MS analysis of selected bi-, tri- and tetra-antennary N-glycans. Isomers of 1946 Da (i.e., *m/z* 1947; $Hex_3HexNAc_6Fuc_1$-PA) present in female and male HIAX fractions eluted on the RP-amide column between 8.3 and 12.0 g.u. (Figure 1, chromatograms D and I; see also ***Supplementary Figure 5***, panels S and T). **A-C:** The 8.3 g.u. isomer possessed an *m/z* 407 B2 fragment compatible with an antennal $HexNAc_2$ modification; due to this fragment, this glycan was targeted for treatment with *C. elegans* HEX-4 β1,4-*N*-acetylgalactosaminidase and the *m/z* 1744 digestion product lacked the m/z 407 fragment, thus defining the $HexNAc_2$ motif as LacdiNAc (GalNAcβ1,4GlcNAc). In MS/MS, the successive loss of 406, 203 and then 162 Da culminating in a major *m/z* 1176 Y3 fragment ($Hex_2HexNAc_3Fuc_1$-PA) is compatible with the presence of two antennae on the α1,6-mannose (one with LacdiNAc and the other just with GlcNAc); one HexNAc was sensitive to *Xanthomonas* β1,2-*N*-acetylglucosaminidase indicative that there was at least one terminal β1,2-GlcNAc not substituted by GalNAc, whereby the MS/MS shows a predominant *m/z* 973, rather than a *m/z* 1176, fragment. An isomeric structure (eluting at 8.5 g.u.; ***Figure 1***, chromatogram I) is also sensitive to HEX-4; after removal of the GalNAc from both variants, the products co-elute with a typical nematode triantennary glycan with one β1,6- and two β1,2-GlcNAc residues (8.2 g.u.). The 8.5 g.u. structure is also sensitive to *Xanthomonas* *N*-acetylglucosaminidase, but loses two GlcNAc residues (not shown), compatible with the GalNAc being on the β1,6-GlcNAc rather than on the 'upper' β1,2-GlcNAc as for the 8.3 g.u. isomer.

D and E: The 12.0 g.u. isomer also possessed an *m/z* 407 B2 fragment, but lost two HexNAc residues upon HEX-4 digestion; based on a co-elution experiment with the product, it was concluded that this isomer is biantennary and carries two LacdiNAc motifs. The inset is a 'zoom' around the region of the detected ions indicating the monoisotopic resolution of the $[M+H]^+$, $[M+Na]^+$ and $[M+K]^+$ species. **F:** The MS/MS of the 9.8 g.u. isomer lacks an m/z 407 LacdiNAc fragment, but possesses one at *m/z* 1379 compatible with a tetra-antennary structure with no LacdiNAc; the m/z 1801 glycan in the same RP-amide fraction (for MS/MS see ***Supplementary Figure 5R***) is the non-fucosylated version of the 12.0 g.u. *m/z* 1947 isomer.

Supplementary Figure 7:

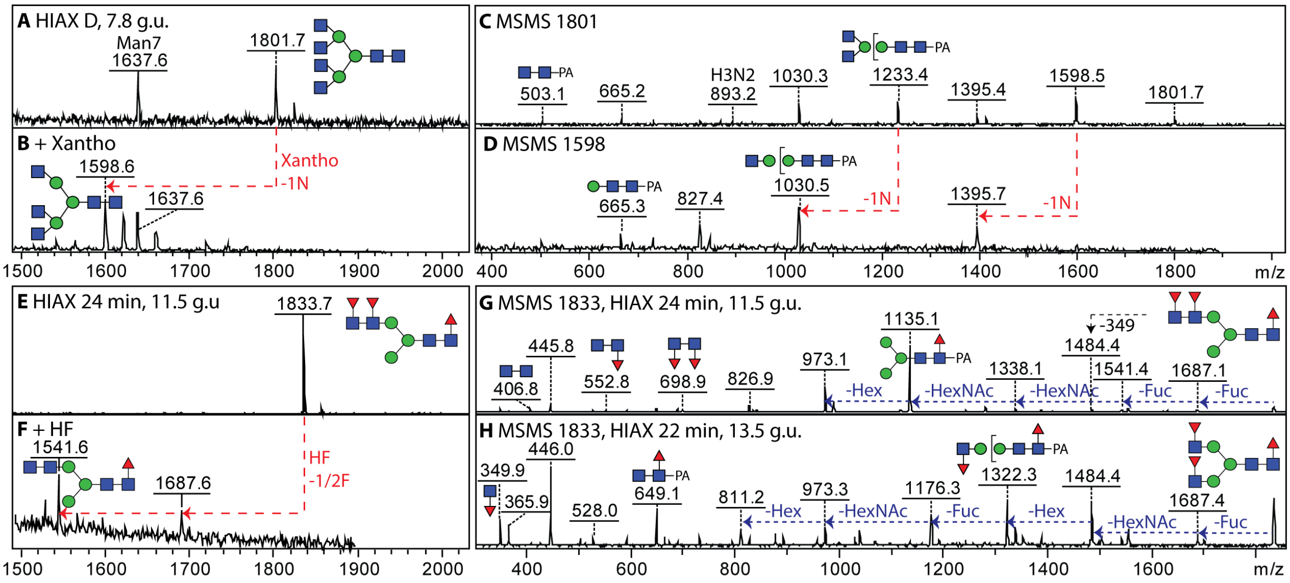

Analysis of a non-fucosylated tetra-antennary structure and of two isomeric trifucosylated glycans. A-D: The tetra-antennary non-fucosylated *m/z* 1801 glycan with just GlcNAc on the antennae (7.8 g.u.; see also ***Supplementary Figure 5Q***) lost no HexNAc residues when treated with *C. elegans* HEX-4 (not shown) or one HexNAc when treated with *Xanthomonas* GlcNAcase. The tendency for this enzyme to only cleave a single GlcNAc from tetra-antennary glycans (as opposed to two from normal biantennary glycans) is presumably due to steric hindrance by the β1,4-GlcNAc residue (also as observed for the triantennary asialoagalacto fetuin glycan; not shown). Note that the major fragments in panel C are 146 mass units lower than in ***Supplementary Figure 6F***, which indicates the relatedness of the fucosylated and non-fucosylated tetra-antennary structures; the MS/MS in panel F is complicated by the losses of fucose from the major fragments. **E-H:** A comparison of one pseudohybrid and one biantennary trifucosylated glycan (*m/z* 1833; $Hex_3HexNAc_4Fuc_3$-PA, isolated from two female 2D-HPLC fractions), which display different fragmentation patterns. The MS spectra (left; E and F) show a 2D-purified glycan with *m/z* 1833 (eluting at 24 minutes on HIAX and at 11.5 g.u. on RP-amide) before and after HF digestion showing serial loss of the two antennal fucose residues, while the MS/MS spectra (right; G and H) show the comparison of its fragmentation with that of the isomer eluting at 22 minutes on the HIAX column and 13.5 g.u. on RP-amide. The *m/z* 699 fragment in the first MS/MS is indicative of a difucosylated chitobiose antenna, but this ion is absent from the spectrum of the later eluting isomer, which otherwise displays a different set of B and Y fragments. These glycans from the female glycome correspond to structures also found in the male glycome (***Figure 1***, chromatograms C and I). Note also that only the 11.5 g.u. isomer was sensitive to jack bean α-mannosidase (data not shown) and co-eluted with the FDL digestion product of *m/z* 2036, a glycan otherwise shown to also have a chitobiose-based antenna as judged by chitinase digestion (see ***Supplementary Figure 8 H-K***).

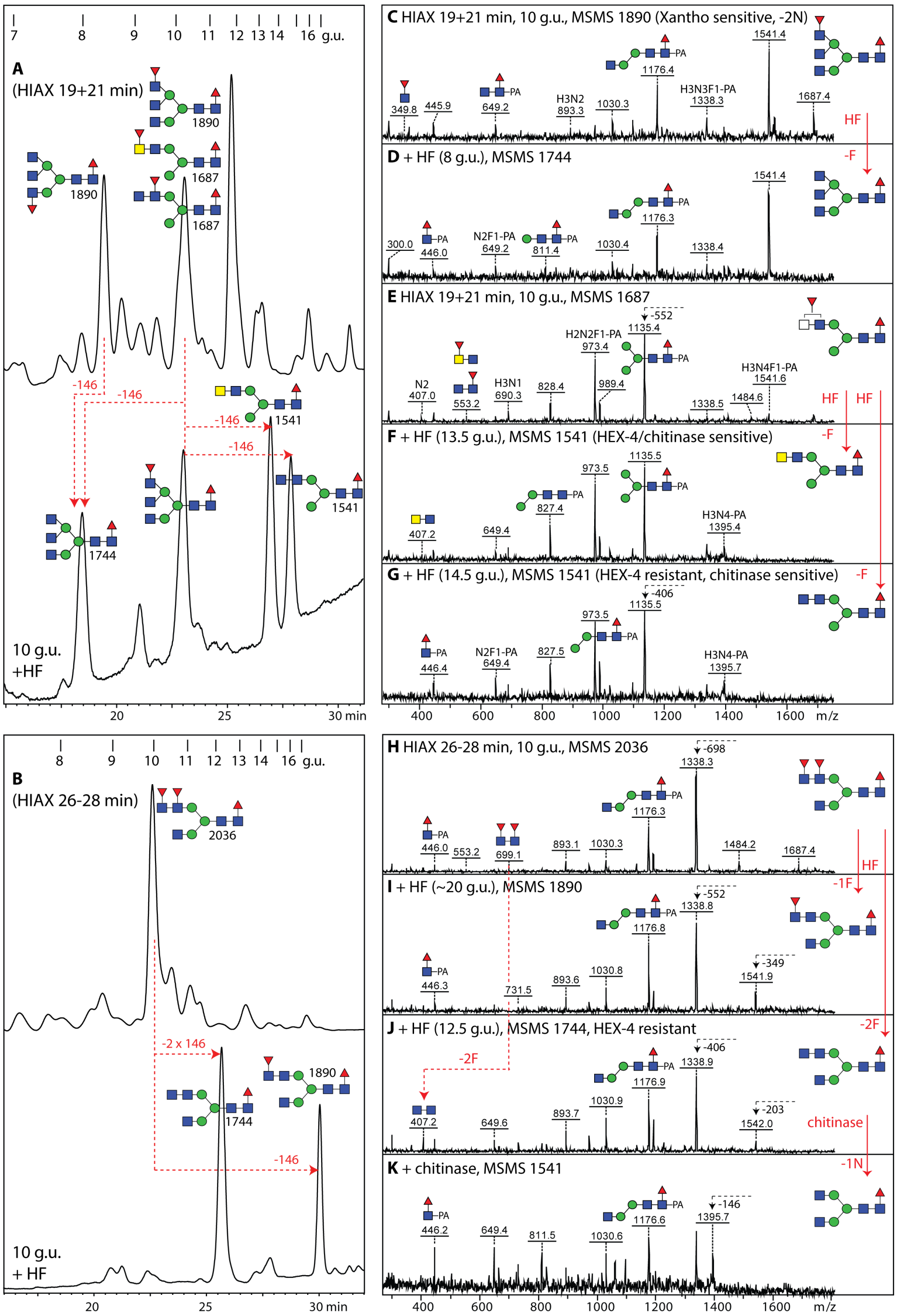

Supplementary Figure 8: Analysis of antennal fucosylation on single GlcNAc or di-HexNAc motifs. (A and B) The alteration in RP-amide elution positions of example fucosylated glycans upon hydrofluoric acid (HF) treatment is shown for two different HIAX pools; shifts in elution of individual 2D-fractions are shown by the dashed lines, whereby the HF-treated 8.5 g.u. glycan co-elutes with one defucosylated product from the mixed 10 g.u. fraction, thereby indicating the identity of the underlying tri-antennary structure. **(C-K)** Corresponding MS/MS spectra for untreated and treated glycans show losses of key B-fragments such as *m/z* 349, 553 or 699 ($HexNAc_{1\text{-}2}Fuc_{1\text{-}2}$) upon hydrofluoric acid treatment, indicative for antennal α1,3-fucosylation; note that the core α1,6-fucose is resistant to this treatment as shown by the retention of the *m/z* 446 $Fuc_1GlcNAc_1$-PA Y-fragments. The differential sensitivity to HEX-4 and chitinase of the two antennally defucosylated *m/z* 1541 products with different elution times (panels F and G), despite co-elution before defucosylation, and the chitinase sensitivity of the *m/z* 1744 defucosylation product (panel K) verify the underlying antennal motifs. From the pattern of changes in elution after defucosylation, it can be concluded that antennal fucosylation is normally associated with earlier retention, but the terminal/subterminal position or the exact nature of the underlying $HexNAc_2$ had a differential impact on the RP-amide retention time. See ***Figures 3 and 4*** as well as ***Supplementary Figures 7 and 9*** for further examples of fucosylated N-glycans.

Supplementary Figure 9:

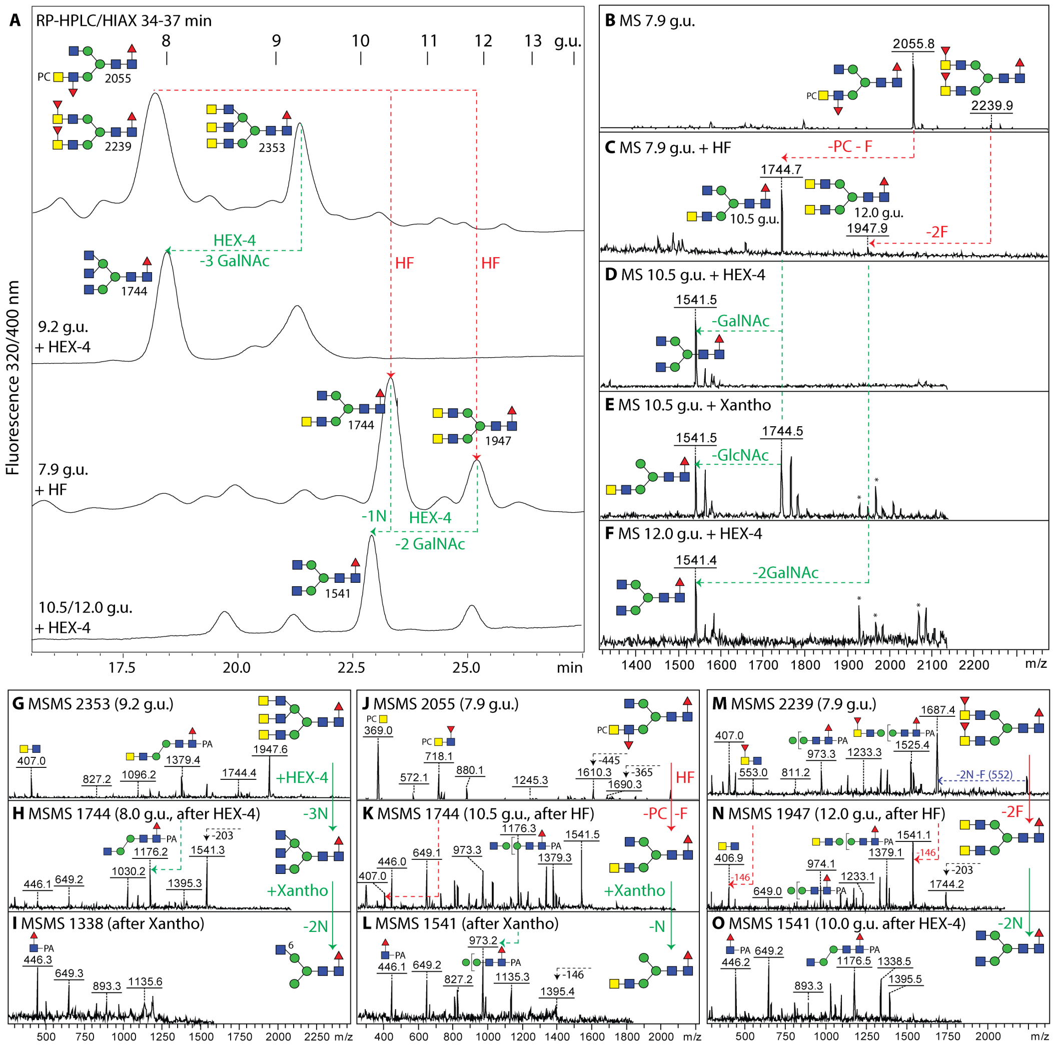

Example structural assignments aided by hydrofluoric acid and hexosaminidase treatments. (A) A section of the RP-amide chromatogram of an example neutral HIAX pool (eluting at 34-37 min; similar to female pool G as shown in ***Figure 1***, but from the second female preparation) with two peaks annotated (7.9 and 9.2 g.u.). The effect on elution time due to either *C. elegans* HEX-4 treatment of the 9.2 g.u. fraction (green lines) or serial HF/HEX-4 treatment of the 7.9 g.u. fraction (red then green lines) is shown (the HF products at 10.5 and 12.0 g.u. were pooled prior to HEX-4 treatment). **(B-F)** The corresponding positive mode MALDI-TOF MS spectra for the treatments of the 7.9 g.u. fraction as well as after *Xanthomonas* β1,2-specific *N*-acetylglucosaminidase (Xantho) treatment of the HF-treated 10.5 g.u. product; removal of fucose, phosphorylcholine or GlcNAc/ GalNAc is accompanied by losses of 146, 165 or 203 Da. **(G-O)** Positive mode MALDI-TOF MS/MS of the original glycans as well as their digestion products; the shifts in the fragmentation patterns correlate with the removal of fucose, phosphorylcholine or GlcNAc/GalNAc (-F, -PC, -N) due to the HF, HEX-4 or *Xanthomonas* hexosaminidase treatments. The loss of three and then two HexNAc residues upon HEX-4 and *Xanthomonas* hexosaminidase treatment of the *m/z* 2353 glycan correlates with its triantennary structure (G-I), whereas the loss of fucose and phosphorylcholine from the same antennae of the *m/z* 2055 glycan is shown by the disappearance of the *m/z* 718 B-fragment (J-L); the presence of terminal fucosylated GalNAc residues on the *m/z* 2239 glycan is demonstrated by the smallest Y-fragment with two fucoses being at *m/z* 1525 and by the loss of the *m/z* 553 fragment after HF treatment, which reveals the underlying HEX-4-sensitive GalNAc residues (M-O).

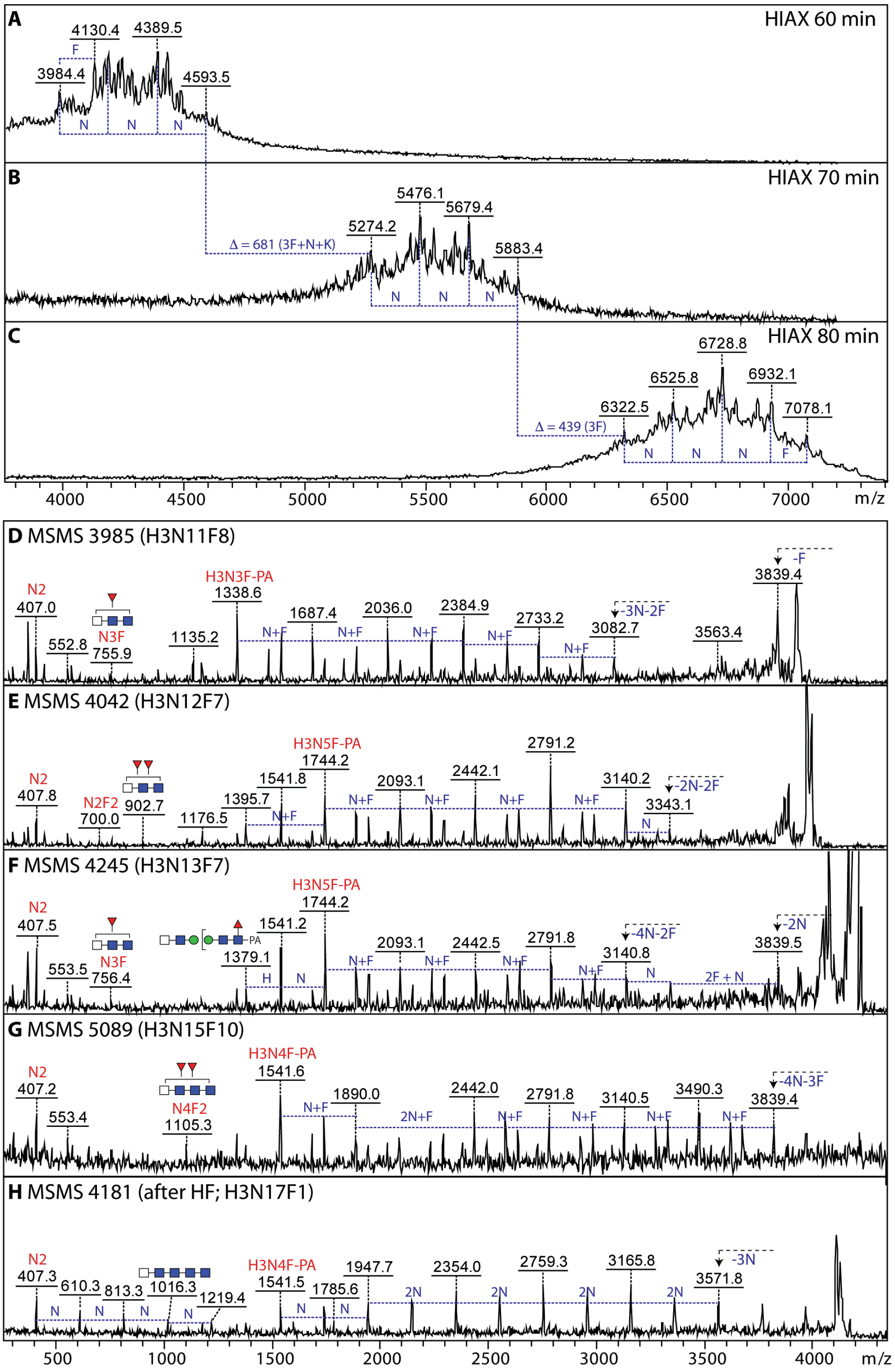

Supplementary Figure 10: Example MALDI-TOF MS data for late-eluting neutral HIAX fractions.

(A-C) Linear positive mode MALDI-TOF MS of three example HIAX fractions (60, 70 and 80 minutes); differences due to fucose and HexNAc are shown; assuming the presence of potassium adducts and taking account of the relative inaccuracy of linear mode, the *m/z* 7078 ion may correspond to $Hex_3HexNAc_{21}Fuc_{15}$.

(D-H) Example MALDI-TOF MS/MS of glycans (internally re-calibrated) eluting between 60 and 70 minutes as well as one example of a glycan detected after rechromatography following hydrofluoric acid treatment of a pool of glycans eluting between 30 and 80 minutes.

Key fragments indicative of multiply fucosylated HexNAc-series are depicted in addition to relevant *m/z* differences; considering the occurrence of the $HexNAc_{2-6}$ *m/z* 407-1219 B-fragments after HF treatment, the presence of fucosylated $HexNAc_{2-6}$ antennae can be assumed. The single fucose remaining after HF treatment is assumed to be α1,6-linked to the core.

Supplementary Figure 11:

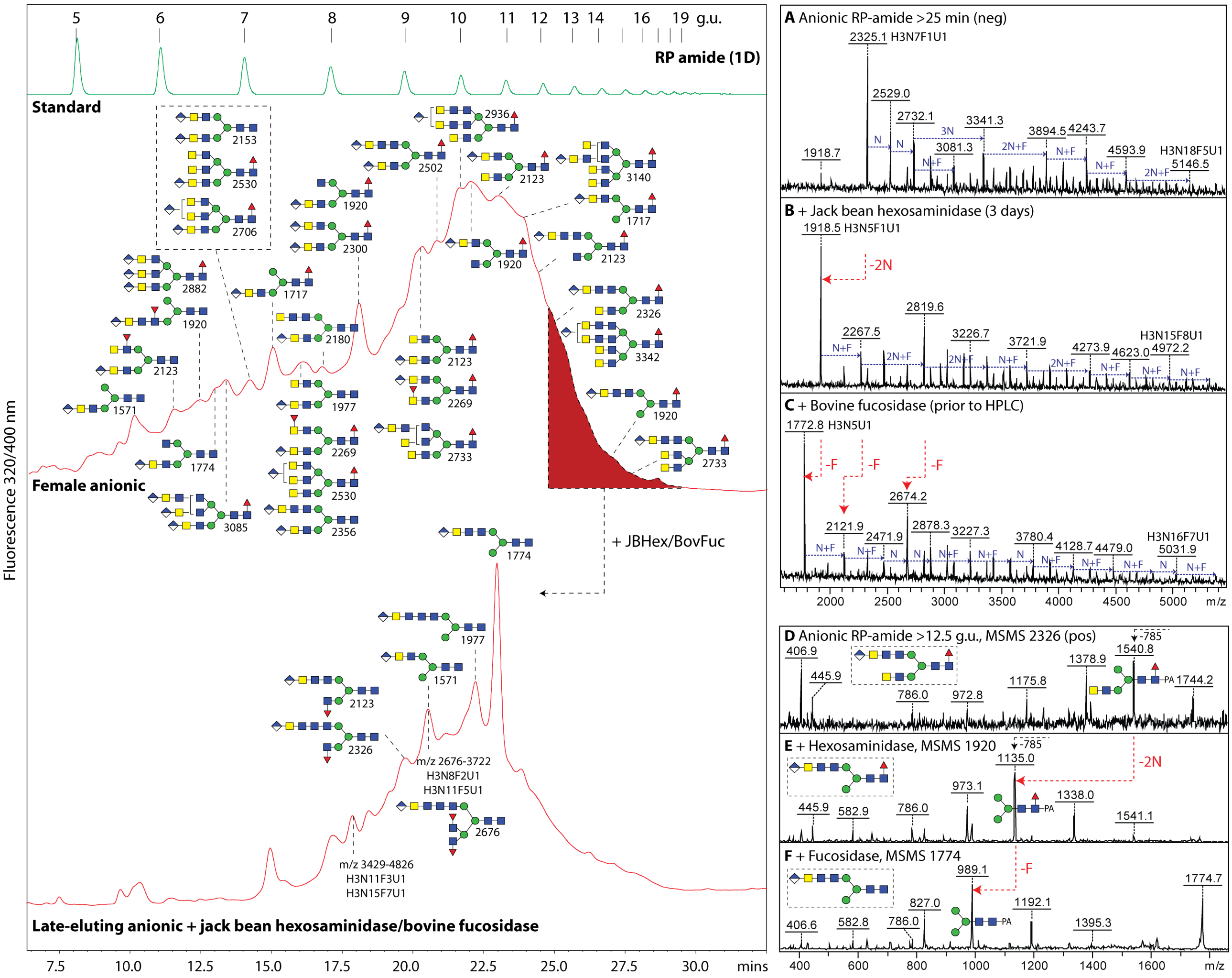

Effect of hexosaminidase and fucosidase digestion on *Dirofilaria* anionic N-glycans. The RP-amide HPLC chromatogram of the complete female anionic pool as compared to the oligoglucose standard is annotated with those structures detected also in the 2D-analysis (***Figure 5*** in the main text) as judged by MALDI-TOF MS of the individual fractions (positive mode *m/z* values being indicated). Particularly the later part of the chromatogram shows a rather unresolved set of fractions. The fractions eluting later than 25 minutes (12 g.u.) were pooled and subject to serial jack bean β-*N*-acetylhexosaminidase and bovine α-fucosidase treatment - subsequent rechromatography showed a shift in the elution properties compatible with the loss of a core α1,6-fucose, but retention of the α1,6-antenna; those glycans lacking antennal fucose were digested down to structures of *m/z* 1571, 1774 and 1977 (9-11 g.u.) with one glucuronylated antenna, the first two co-eluting with proven structures in the 2D-analysis (see ***Figure 5 A and B*** in the main text). The other annotations of the digested fractions are based on MS/MS, whereas the *m/z* and composition ranges for larger glycans are indicated without a structural proposal. **(A-C)** Negative mode MALDI-TOF MS of the late-eluting pool before and after serial jack bean β-*N*-acetylhexosaminidase and bovine α-fucosidase treatments, showing a shift in the major N-glycan from *m/z* 2325 to *m/z* 1772 suggesting the loss of two HexNAc residues and one fucose; the continued occurrence of glycans containing HexNAc/Fuc repeats is due to the relative resistance of antennal α1,3-fucose to bovine fucosidase. **(D-F)** Corresponding positive mode MALDI-TOF MS/MS of the major N-glycans in the pool before and after glycosidase treatment, showing the loss of HexNAc residues from the lower (non-glucuronylated) arm and of the core fucose (loss of the *m/z* 446 Y-fragment).

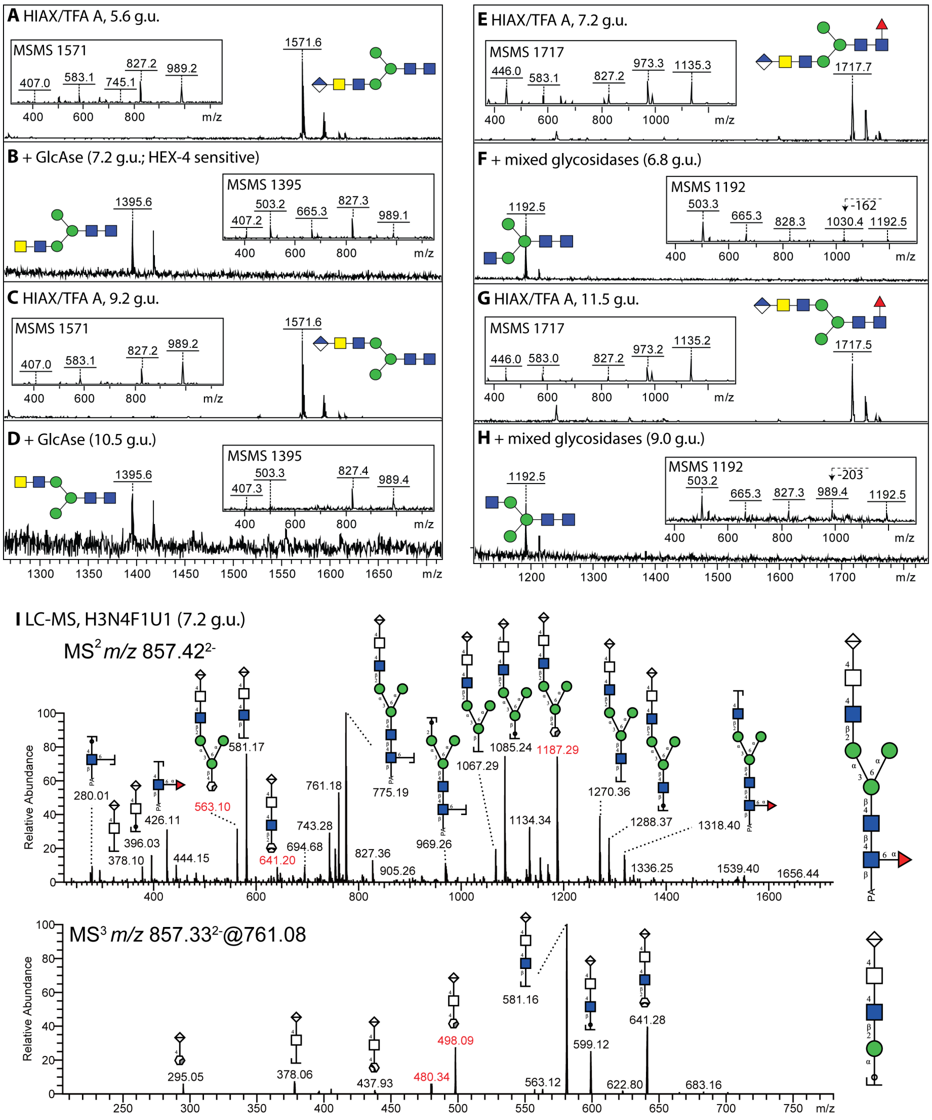

Supplementary Figure 12: Further analyses of *Dirofilaria* monoglucuronylated N-glycans. (A-D) Positive mode MALDI-TOF MS of two isomeric anionic 2D-HPLC-purified N-glycans before and after *H. pomatia* β-glucuronidase treatment and rechromatography showing the shift to later elution time (see ***Figure 5*** of the main text for the relevant HIAX and RP-amide chromatograms; the insets show the key region of the corresponding MS/MS spectra). **(E-H)** Positive mode MALDI-TOF MS of two fucosylated isomeric anionic 2D-HPLC-purified N-glycans before and after a ‘mixed glycosidase’ treatment (i.e., combined incubation for 4 hours with β-glucuronidase, β-*N*-acetylgalactosaminidase and α-fucosidase): the elution positions of the $Hex_3HexNAc_3$ glycan products are indicative of the presence of either an α1,3- or an α1,6-antenna with the shift to earlier elution due to the loss of core α1,6-fucose. The insets show the key regions of the corresponding MS/MS spectra. **(I)** To determine the linkage between HexNAc and β1,2-linked GlcNAc, a glycan with composition of $Hex_3HexNAc_4HexA_1Fuc_1$-PA ($[M-2H]^{2-}$ ions at *m/z* 857; the same 2D-purified glycan as shown in panel E) was analysed by negative mode LC-ESI-MS^n. Shown are the major fragments for MS^2 of ions at m/z 857.42 ($Hex_3HexNAc_4HexA_1Fuc_1$-PA, $[M-2H]^{2-}$ ions) and MS^3 of ions at m/z 761.08 ($Hex_1HexNAc_2HexA_1$, $[M-H]^-$ ions). Due to space limitations, only annotations of major fragmentation ions are shown with key diagnostic ions in red. Fragmentation ions at *m/z* 563 and 1187 are annotated as $^{2,4}A$ and $^{0,2}A$ cleavage of the penultimate GlcNAc, both being diagnostic for N-glycans as described by Everest-Dass *et al.* *[5]*. The presence of fragmentation ions at *m/z* 641 ($HexNAc_2HexA_1$ + 59Da) and absence of “D ions” at *m/z* 887 suggests that the $HexNAc_2HexA_1$ motif is linked to Man on the 3-antenna rather than the 6-antenna. No fragment ions indicated the presence of terminal HexNAc. Instead, fragment ions at *m/z* 1318 and 378/396 suggest a linear HexA-HexNAc linked to β1,2-linked GlcNAc on the 3-antenna. To determine the linkage between HexA-HexNAc and GlcNAc, MS^3 of fragmentation ions at *m/z* 761, which contain $HexNAc_2HexA_1$ plus the 3-antennal Man, was performed. Fragment ions at *m/z* 498/480 ($^{0,2}A_{GlcNAc}$/ $^{0,2}A_{GlcNAc}$-H_2O) suggest the presence of HexA-HexNAc linked to C_4 of GlcNAc, which is compatible with the enzymatic digestion data with the specific HEX-4 hexosaminidase.

Supplementary Figure 13:

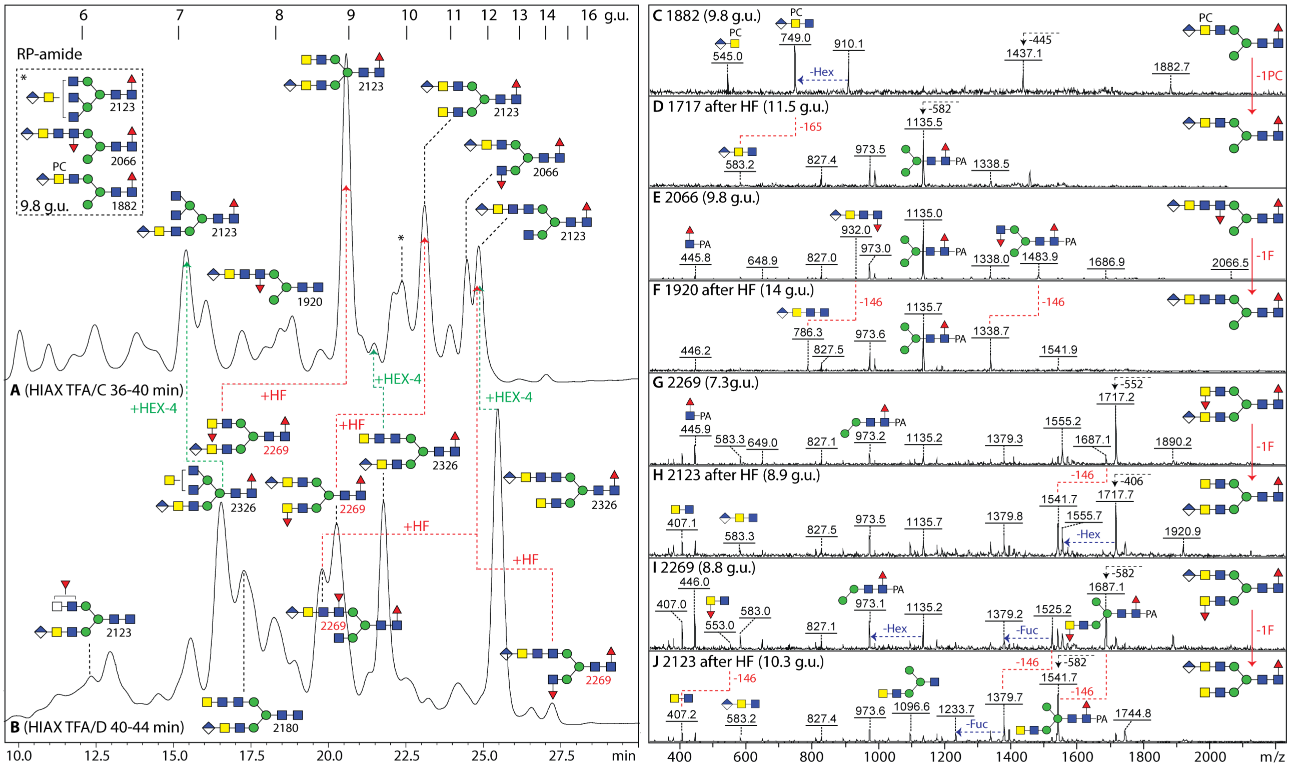

Effect of chemical and enzymatic treatments on elution and fragmentation of monoglucuronylated N-glycans. (**A and B**) RP-amide HPLC comparison of two anionic HIAX pools (see also **Figure 5 C and D**) showing the effect of hydrofluoric acid (HF) or *C. elegans* HEX-4 β1,4-N-acetylgalactosaminidase treatments of selected pool D RP-HPLC fractions and shifts to the same retention time as some pool C glycans; for reasons of space, three glycan structures are boxed and elute at the position indicated with an asterisk. **(C-J)** Corresponding positive mode MS/MS data for selected pool C and D glycans before (C, E, G and I) and after (D, F, H and J) hydrofluoric acid treatments showing the loss of phosphorylcholine (*Δm/z* 165) or of antennal fucose (*Δm/z* 146) on the same or a different arm as compared to the glucuronic acid residue; removal of phosphorylcholine or fucose is accompanied by losses of some B-fragments (e.g., loss of *m/z* 749, 932 or 553 and appearance of *m/z* 583, 786 or 407) or shifts in the Y-fragmentation pattern (e.g., from *m/z* 1484 or 1687 to 1338 or 1541) as well as alterations in retention time (see arrows between the two chromatograms in panels A and B; refer also to further MS/MS of *m/z* 2123, 2269 and 2326 glycans in ***Supplementary Figure 14***).

Supplementary Figure 14:

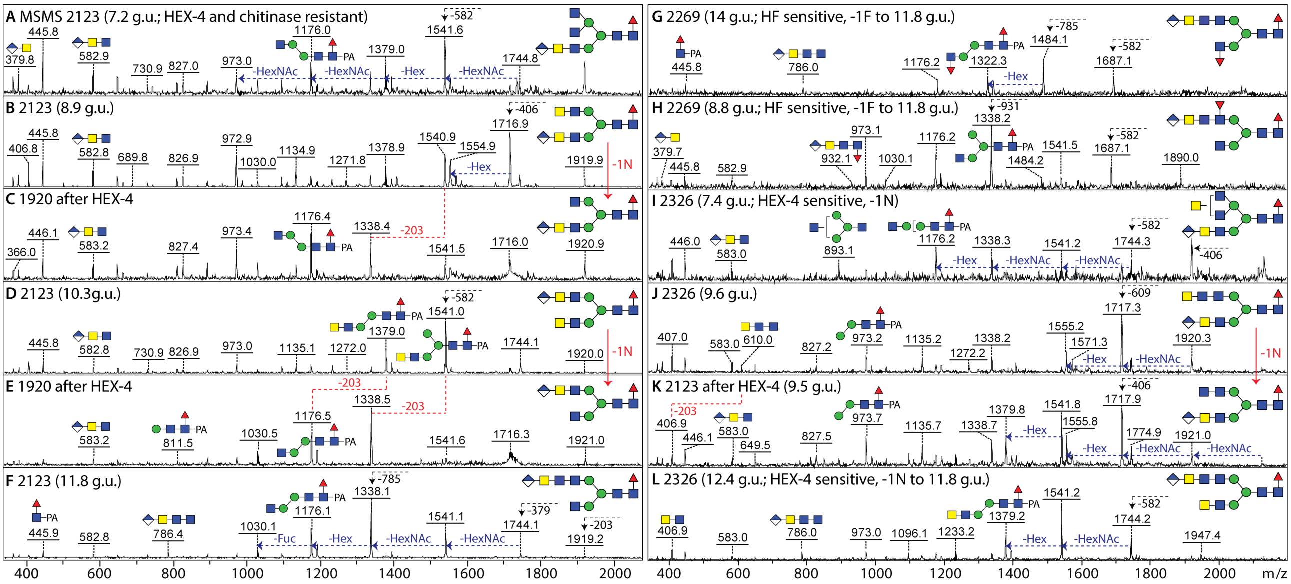

Further examples of MS/MS of monoglucuronylated N-glycans. (A-F) Positive mode MS/MS fragmentation of different *m/z* 2123 isomers occurring in different RP-amide fractions (see ***Supplementary Figure 13A***) or two corresponding HEX-4 β-*N*-acetylgalactosaminidase products (C and E) which differ in antennal substitutions (e.g., B; *m/z* 1555 fragment showing loss of the 6-antenna as compared to panel D), sensitivity towards enzyme treatments or in antennal length. **(G and H)** Positive mode MS/MS spectra of two further *m/z* 2269 glycans indicating that the glucuronic acid is on a longer antenna (*m/z* 786 or 932 fragments); the noted hydrofluoric acid sensitivities resulting in shifts to 11.8 g.u. correlate with identical backbone structures carrying antennal α1,3-fucose in different positions. **(I-L)** Positive mode MS/MS fragmentation of different *m/z* 2326 isomers occurring in different RP-amide fractions or one corresponding HEX-4 β-*N*-acetylgalactosaminidase product (panel K) which was re-applied to RP-amide with the shift in g.u. as indicated on the chromatogram (***Supplementary Figure 13B***); losses from the parent ion of $HexA_1HexNAc_2$ ($\Delta m/z$ 582), $HexNAc_3$ (609) or $HexA_1HexNAc_3$ (785) are shown in addition to selected B- and Y-fragments and shifts thereof upon HEX-4 digestion. Proofs for the structure of the underlying $HexNAc_3$ motif as being GalNAcβ1,4GlcNAcβ1,4GlcNAc are shown in **Figures 7 and 8**. Other isomers of *m/z* 2123 and 2326 glycans with different RP-amide retention times and sensitive to *Xanthomonas* β1,2-*N*-acetylhexosaminidase exist, but their exact antennal status is ambiguous and so not presented.

Supplementary Figure 15:

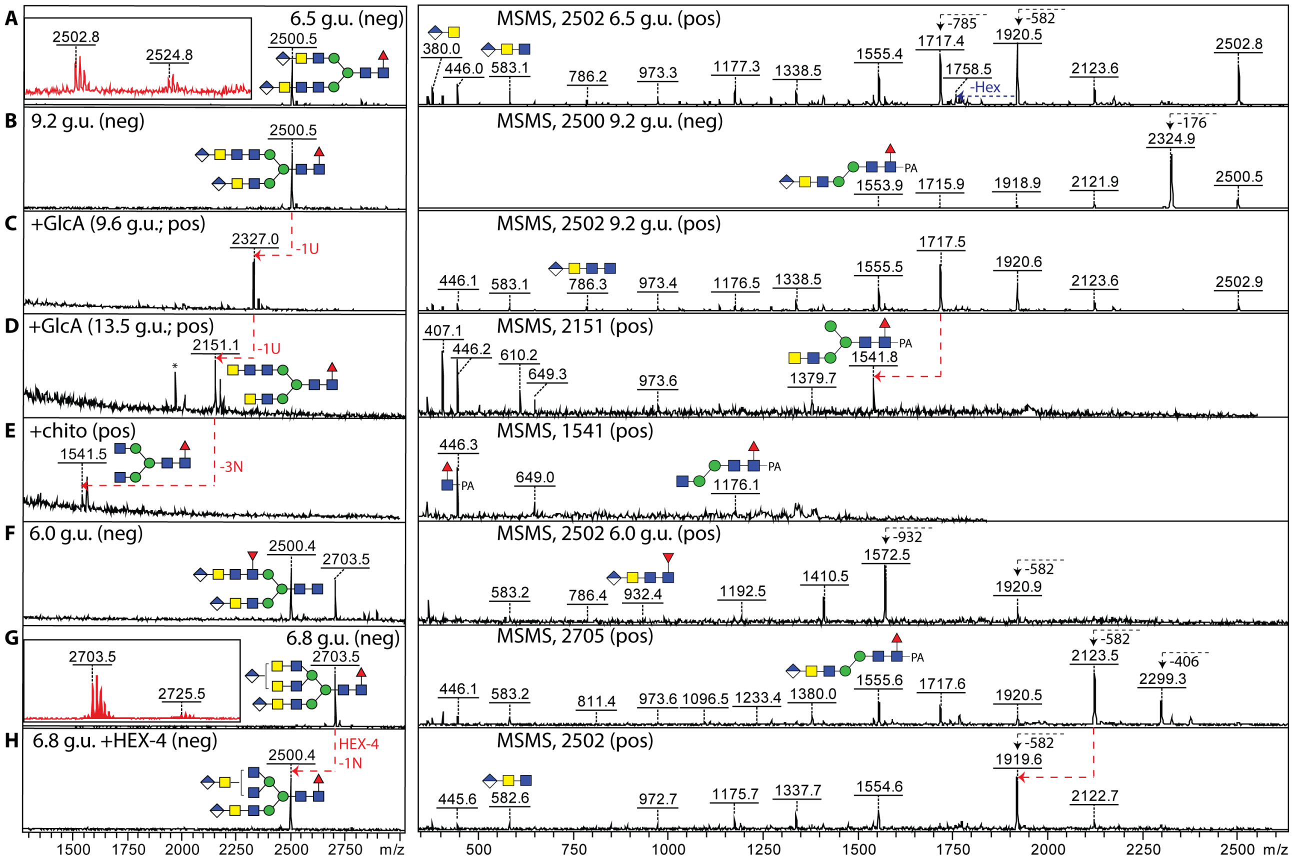

Comparative MS/MS of bi- and tri-antennary bi-glucuronylated N-glycans. Upon the second dimension separation of HIAX fractions H and I (see ***Figure 5***), three isomers of *m/z* 2500/2502 of different RP-amide retention times (in g.u.) were found.

(A) Negative mode MS and positive mode MS/MS of a 6.5 g.u. RP-amide fraction; the inset shows the monoisotopic resolution of the $[M+H]^+$ and $[M+Na]^+$ in positive mode. The isomeric status was inferred from the generally lower retention of glycans with a longer 'lower' arm.

(B-E) MS (left) and MS/MS (right) of a 9.2 g.u. RP-amide fraction (see also ***Figure 8*** in the main text); the negative MS/MS spectrum only shows a series of Y-ions, as compared to the mix of B- and Y-ions in the positive MS/MS spectrum shown beneath. Positive mode MS of the first and second glucuronidase products (C and D) after a second round of RP-HPLC show the serial loss of the glucuronic acid residues and a shift in the MS/MS spectrum, whereby the *m/z* 583 and 786 B-ions are replaced by those at *m/z* 407 and 610. Subsequent chitinase treatment of the 13.5 g.u. glucuronidase product results in a loss of three HexNAc residues (E), whereas the capping with terminal GalNAc was defined on the basis of HEX-4 digestion resulting in loss of two GalNAc residues (data not shown); the low intensity of the signals is due to a mere 1 mV (0.4 pmol) fraction being used for the digest.

(F) Negative mode MS and positive mode MS/MS of a 6.0 g.u. RP-amide fraction; as there is no *m/z* 446 fragment, but rather one at *m/z* 932, it is concluded that the single fucose residue is associated with the antennal $HexA_1HexNAc_3$ motif.

(G and H) Negative mode MS and positive mode MS/MS of a 6.8 g.u. RP-amide fraction before and after HEX-4 digestion (the inset shows the monoisotopic resolution of the $[M-H]^-$ and $[M-H+Na]^-$ in negative mode); the loss of a GalNAc is accompanied by a shift in the major Y-ion from *m/z* 2123 to 1920.

Supplementary Figure 16:

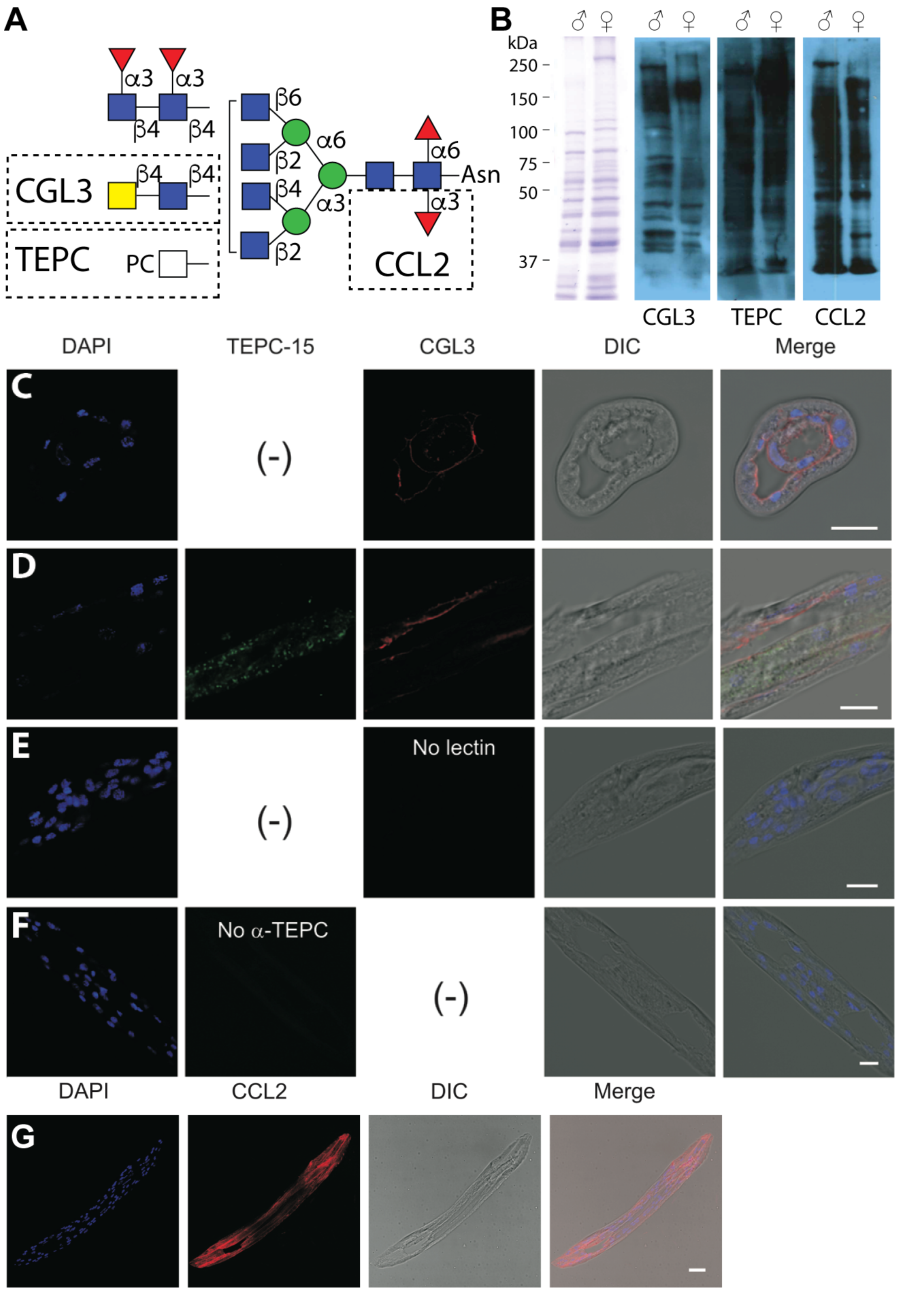

Epitope detection in *D. immitis* by blotting and microscopy. **(A)** A schematic depiction of a neutral *D. immitis* N-glycan showing the potential epitopes for two *Coprinopsis* lectins (CCL2 recognising core α1,3-fucose and CGL3 recognising β1,4-GalNAc/GlcNAc) and the murine IgA monoclonal TEPC-15 (recognising phosphorylcholine). **(B)** The presence of HexNAc, Fuc and phosphorylcholine residues was probed using CGL3, CCL2 lectins and TEPC-15 by Western blotting of *D. immitis* male and female adult protein extracts. **(C-G)** Indirect immunofluorescence microscopy using biotinylated forms of CGL3 and CCL2 or TEPC-15 on fixed tissue sections of L3 larvae: CGL3 binds to the internal surfaces of the worm, including the gastro-intestinal tract (cross and longitudinal sections; C and D); CCL2 detects epitopes present in every tissue of the parasite (G); TEPC-15 staining presented a dotted pattern in the gastrointestinal tract of the L3 parasite larvae, partially distinct from the co-staining with CGL3 (D). No signal was observed for larvae probed with Atto 655 conjugated forms of streptavidin or anti-IgA in the absence of primary lectin/ antibody (negative controls in panels E and F). DAPI (blue) stains nuclear DNA; DIC, Differential Interference Contrast; white scale bars are 10 μm.

Supplementary Figure 17:

Top 5 Mascot hits in each sample

Control	Score
1. nDi.2.2.2.t03886, apolipophorin	11828
2. nDi.2.2.2.t05723, enolase	4538
3. nDi.2.2.2.t00559, heat shock protein 70 (hsp-1)	3822
4. nDi.2.2.2.t02994, heat shock protein 90 (hsp-90)	3100
5. nDi.2.2.2.t06238, probable phosphoglycerate kinase (pgk-1)	2848

CGL3-bound	Score
1. nDi.2.2.2.t03886, apolipophorin	3090
2. nDi.2.2.2.t04547, actin	2054
3. Swissprot O18840, ACTB_CANLF, dog actin	2033
4. nDi.2.2.2.t00559, heat shock protein 70 (hsp-1)	1949
5. nDi.2.2.2.t05723, enolase	999

TEPC15-bound	Score
1. nDi.2.2.2.t03886, apolipophorin	5208
2. trNCBI Z4YHI2, Z4YHI2_CANLF, dog actin	2117
3. nDi.2.2.2.t04547, actin	1786
4. Di.2.2.2.t00559, heat shock protein 70 (hsp-1)	1407
5. nDi.2.2.2.t05723, enolase	1182

```
>nDi.2.2.2.t03886-RA,apolipophorin
Protein sequence coverage: 32%
MHLRWLLPLQAVITWTVAYDNNHHTTTTNDTCATICTGVSSDVKYDIGHSYVFDVTSRTILKIGQEQDTDVIQNAQAHISVHSSCEFSLKLTRTSI
RGMDVGNDWSSILERSSLRFAFDDGEVKAVCPDDNDPTWAVNIKRAILSAFQTKYEGAREEDIHGSCPVNIEKRKSNEMLNLKTSKQLNACYREHD
IAGVRTIPYRLESNMQIAPMMETKQICERQIVHYNLQQVTCTEDYRVVSPFHESNLGTLHVEQTLRAVGIAAAPQQESFKERLSIIFDHSNDNFYT
KSSPQFAKLIIEELCRSDDRVAPDAASHFSDLVYNLRGLSNGEISSIANLRCDAFIDALAACASHACLLQLGNFINSGAASESLYSSLSLLPNPKK
GIMDSVASFIERVPIHGLLAVSSLVQSYCIAHPICGMEPAVQRIVHSISSKLPPGCQVIHQFEEIKEAIIILKSIGNIGYEEHSLSSILKCVANDR
IPKEVKIAAIDALRRKPCNDQRNSKITQLFRDQKENVEVRIISFRQLMECVDDEIMQIVVDQLYNETINQVGSYIWSYLNTKQRSTNPGSRNLQHL
LKRFHVPQRYNLDSNRFSHYYELGYFDRENNYGGHMDTSILLAPNGYVPREVAFNFTVHLFGKSVNILEIGVRAEGMEEAEEELFGPDGYISNPNG
HVFRKKRFQSRYPKLNHLKELYRKKNSGIDNRMRASFYVRMFGDDLHYYGFQKDQYQFLEEVKESIELDKVLSKLMQEKKKKLSRYMLPFELSQTL
PTLSGLSLQFLVNISVATKIDSKLQLNLVNLLQRKSSANGHLDLRPSISVARGSVILLRAGHIASGAMIKSSLHAATSVVQSMEMNEGRKLSLKID
VPHKDLLHAKIRNDIIKIESNRHQPSVIGRHIHDATEKHYCSGDTMSKILGIQACINLHADYSKIEGDIKVEKVDRLLNSYQFVVERVSTASEQKL
LISMDTPGSEVNRKIETDFEISIPRKKLKLGITAPFKTIILDGNLQQMGQLEDYATNLKLIVDDKVYNLDGMLKATKAGERNSYKLNAKSIVESIT
TAEVVAELEYSILKPYAMIDFHLDKIFSKPVIFKTLINPEAPKYEGKLEYSGPDFNGKLDTSIIRQGMIDLKGTVNGEYQIDNHPKQVLEIGLEQA
FQRRGTNYHFKHAMHAVSTAFNKFDFQIFTDRTGNNMKNSLEITYFGEQLIANLDVIRGPNNIYTAIGKMKFDRLGIDNKVDITYQNRFPLQFMMK
IDAETPKIRNIHATAEYVVKIDPKWNFMGNILLRYPDREIAFDKKIDEVTNGQYKMETHLRWDPNSKIDIMSDVIFRPQENEYSIESTANIAGVRE
PINIKKHIKYQPDNYNIQWHAKQGSRTIYELIANVDGQFGDQQRLKIDINSEKFEPRINYHVTAEVQPSLDSITALATIHKNGRHFGTGNIMMPKK
FSLPNQQYRVEFGWIYQDRSRKVAVEYNLLKHTHRSSHAMKVQSDDNLDLNMQFDRHHDKMTLKCDLDKDRIRTISMIFTATPFHWDFFEIDGHLN
TDRPLQRRSLKTKAAFLYRTDQANIQGLFEINDERYGIEGHWRKVLHDNGRQYTYAGKIEIPQNRINLEQQLEVIEAITIRKAKTIFEFINVGRIY
NLTNEIKSNDAFFSTSTDLIGNGIVLKHTIEYNYKHGQRTLKKHLKYNEKEVNIDTVTVYRHGEVKVNIGASSTFDMVRYGKILFDCRKGYIFWNC
DVESNINNQYVVKGHETVSPQNTDIGYLVKLGNVLDTEGKIKFNIDNQASKYNANAMLRRAKVVYNLEMDVNDNKGIVKLQTPTHTIDNPQIRIIR
KGSTEFEIHTEDGSGGRIYAHVKTGNYDKLLKIQITEILEPFELHLENNVIGGKQKSIAELTLDPLGQKRTYGVENEIESEGETFRALRSSLKQPK
RTIKIELLRPAQNKYVFSVQPHVGGRRQPTVAEMTYQKTTDGYHWEGSISDQALKIPLKAKVVYKKDARDQYNYRLDWLTEFVYSDQPDKLFSNSL
HLHRNIIPSKHKKRIAVLHDGRNGNVRFVVELKSIHLASNLNTRLWTKIDRSIIGKVAIPVHATFGLETRNLQRQSTEYSLEIRTDAIKFTEVQLK
SPNSLLKTRINKIKDNHYTVGFYQNNERPSVVGELNLQNNGPVFEYRDERSQEVKLHASAQKLNDYEGKIDVWHSEASKKIRDVLLSVEVKEGNVW
SSKIYLRPTMESEIMKKAKETVSITGDFHQSSFMKNAFLPIFSSHKQIANDVLTTVDQAIKEWAAEHKNFAAELGTEYTDLVDEIEGNYEIIKEAI
LVTYDHAYQEMEQFVKIISNSDFVSQLQQLRGGSIDIQREIMIALKPVLDLIDQFYKEIDDLQRNAAIVIDRLEDTLKLNDLRRALEEYKQNFEQT
DLIQTVLYEMRKLSHRYQLLNLEQTIVDIEINRKRYEGEAFEIYNKIKNFFLDKIIVSALSKVTSAIFDSLDDIQIRQKWKEIINYLRGKITANEM
AKRFWRNYIPTYKRLSPGRYELEVAVPYGVSSLNDVLSNLHPQRFLAFKSRVLESFGLLLKDQEFAQSLSDTIYTYKSAKFNPWKMISLFQSTAYI
INGDRFVSFDGKVFAFHARCEYLLASDLRTQRFALLAVFSSQGHLEAIKTELRDEEVILYKTGNVQVSGAQVSIPWQKIDSIDGAILISVCRKDSW
TILKTYDGLCVRCNSQYDICEIILPGRMHGRSNGLLGLNDNEPSNDRDLVDGTPNDQLNVLAEHWAVNGECRVNQARDLTHRDDDHCQEYFQSFDS
PLRFCFTRIRPKPFEEICSNGGKHQHCTAISAYLEICSNAGIITSLPHECVKCNGDLHLDDERKVEKPIVEHDIVFVVEERKCLDHHKDKIARIAQ
QISQEQRLARFGWVGFGGEGIHHEPLVHYEEDNALLDVSNFMKQTQQSFEPISGIEVPHVCPKKAVEFTIKHFPFRFASAKSIVLVMCRKCPYREH
TEMLDILLEQDITLHLLTLGRFKTSDGSKIIGFDAKQLLNEHGAEIGERTSLLHPHDACTIIAQQTFGTVFSLKHASMVAASRITPKQKSYCFNCQ
CTAHGLFARNICRPCEAVEPAELASYKIIDSESDNELQLLDVFI
```

Mass spectrometric analyses of proteins in the parasite extract: *D. immitis* glycoproteins were enriched by affinity chromatography with either CGL3 (lectin) or TEPC-15 (antibody) and the tryptic peptides were analysed by mass spectrometry as described in the Supplementary Methods. The top five hits for each sample are listed, whereby the top hit for the samples enriched by CGL-3 and TEPC-15 is a predicted apolipophorin with five predicted N-glycosylation sites and was a major hit in a previously published homology-based proteomic analysis of *D. immitis* by Morchón *et al. [6]*; high scores were obtained for common cytosolic proteins contaminating the lectin/antibody pull-down. The hit to dog actin can be explained by the 98% identity of the parasite and canine proteins, which then share a majority of predicted tryptic peptides; thereby, the lack of other canine proteins and absence of canine-type glycans (no sialic acid, no LacNAc) in the analysed glycome is an indication that only the parasite glycome was analysed in this study. The sequence of *D. immitis* apolipophorin is shown with matched peptides in red and the five potential N-glycosylation sites (outside the matched peptides) underlined.

Supplementary Figure 18 (legend on next page):

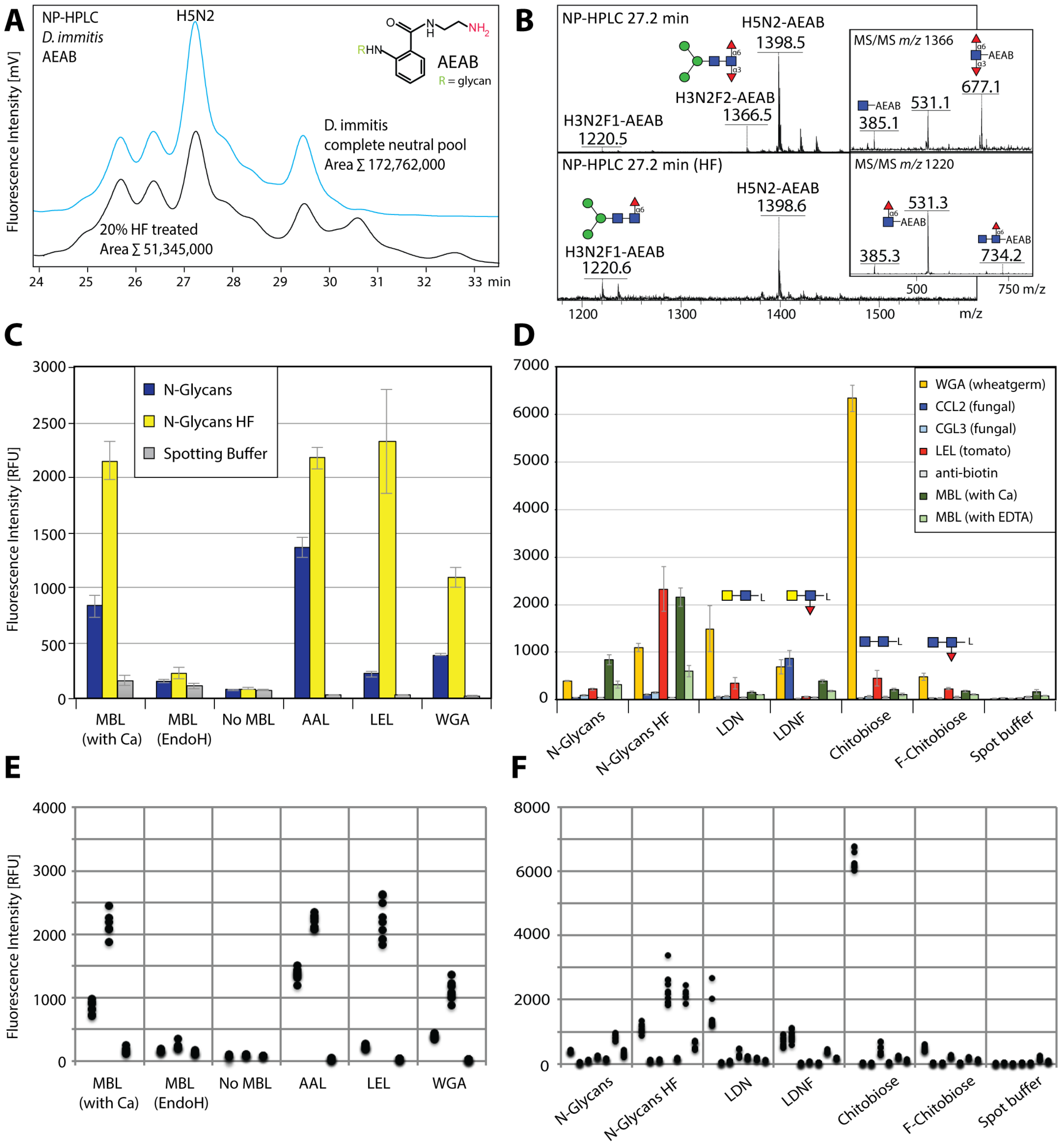

Supplementary Figure 18: Array data with AEAB-labelled N-glycans and synthetic compounds.

(A) NP-HPLC profile of neutral *Dirofilaria* AEAB-labelled N-glycans before and after hydrofluoric acid (HF) treatment to 'strip' α1,3-fucose and phosphorylcholine (PC) residues (see ***Figure 9C*** in the main text for overall MALDI-TOF MS); the total integrated areas (fluorescence) were used to normalise the concentrations before printing. **(B)** MALDI-TOF MS and MS/MS analysis of the major fraction showing loss of the difucosylated structure upon hydrofluoric acid treatment; all other HPLC fractions were also analysed by MALDI-TOF MS before re-pooling.

(C) Array data for binding of human mannose binding lectin (MBL, with or without prior 'on-slide' endoglycosidase H treatment), *Aleuria aurantia* lectin (AAL), *Lycopersicon esculentum* lectin (LEL) and wheatgerm agglutinin (WGA) to AEAB-labelled *Dirofilaria* N-glycans (2.5 fmol glycan per spot) before/after hydrofluoric acid treatment. In the case of MBL, the increase upon HF treatment before printing could suggest that removal of fucose and PC residues reveals longer chains that are then recognized by MBL, but the decrease upon 'on-slide' Endo H treatment would be compatible with binding to oligomannosidic structures (known substrates for this endoglycosidase). AAL binding increases slightly and LEL/WGA binding increase dramatically if the N-glycans are treated with hydrofluoric acid, thereby revealing the preferred binding epitopes of the latter. For a plot with the individual data points, refer to panel **E**.

(D) Binding of wheat germ agglutinin (WGA), *Coprinopsis* CCL2 and CGL3, *Lycopersicon esculentum* lectin (tomato; LEL) and human mannose binding protein (MBL, in the presence of $CaCl_2$ or EDTA) to 'untreated' and 'stripped' neutral AEAB-labelled *Dirofilaria* N-glycans (2.5 fmol per spot) as well as to four di- and tri-saccharides conjugated as 6-(5-aminopentanamido)-*N*-(2-[2-[oligosaccharyl-*N*-methoxyamino]-ethoxy)ethyl]-2-naphthamides (1 fmol per spot; see **Supplementary Figure 23** for remodeling scheme). The N-glycan binding data in *panel D* for WGA, LEL and MBL is an alternative presentation of those shown in *panel C* of this figure and in ***Figure 9D***. For a plot with the individual data points, refer to panel **F**. The average fluorescence values with standard deviations; the corresponding single data points are shown in panels E and F. Conclusions regarding the specificities are given in Supplementary Note 1.

Supplementary Figure 19:

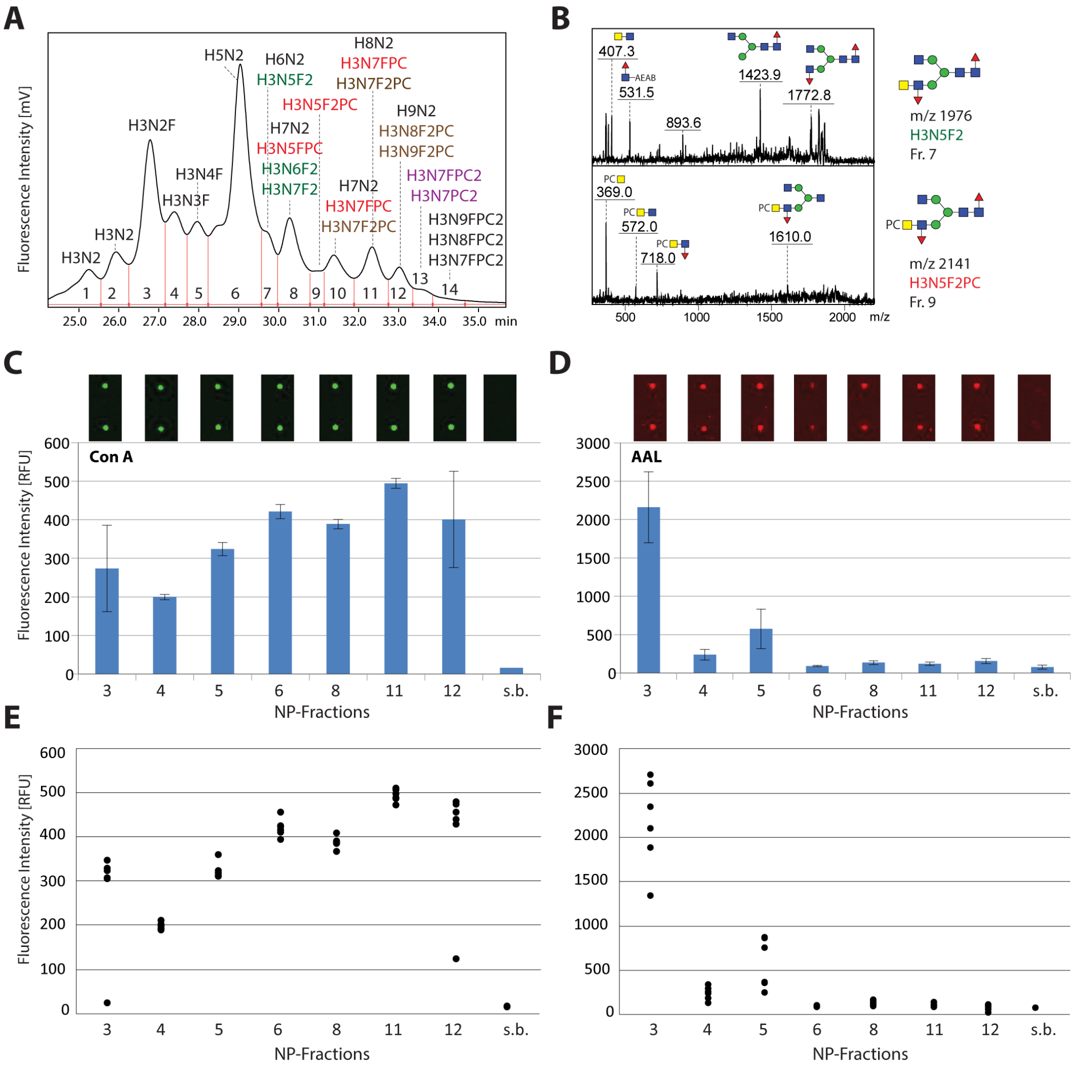

AEAB-labelled N-glycan fractionation and interaction with two standard lectins. (A) NP-HPLC chromatogram of a second preparation of neutral *Dirofilaria* AEAB-labelled N-glycans annotated with fraction numbers as well as compositional abbreviations of selected N-glycans (H, hexose; N, *N*-acetylhexosamine; F, fucose; PC, phosphorylcholine) based on MALDI-TOF MS data. **(B)** Positive mode MALDI-TOF MS/MS of two selected fucosylated or fucosylated PC-modified AEAB-labelled N-glycans ($Hex_3HexNAc_5Fuc_2PC_{0\text{-}1}$; *m/z* 1976 and 2141 as $[M+H]^+$) showing the relevant LacdiNAc and PC-containing B-fragments; compare the MS/MS data for *m/z* 2141 with that for the PA-labelled version (*m/z* 2055) shown in ***Supplementary Figure 9J***. MS/MS of N-glycans in Fractions 13 and 14 predicted to carry two phosphorylcholine residues also yielded the key B-ions of *m/z* 369 and 572. **(C and D)** As a validation of the fractionated glycan array, the seven most abundant HPLC peaks were tested (normalised to ca. 1 fmol per spot based on AEAB fluorescence intensity) with biotinylated forms of concanavalin A (ConA) and *Aleuria aurantia* lectin (AAL); FITC- or AlexaFluor-647-labelled anti-biotin were respectively used for detection. The data indicate that ConA bound to all fractions, which is an indication for the normalization of the printed amounts of glycans; AAL bound strongest to the fractions containing smaller fucosylated N-glycans ($Hex_3HexNAc_{2\text{-}4}Fuc_1$). Images of two example spots of eight in total printed for each glycan are shown; s.b., spotting buffer alone. The average fluorescence values with standard deviations as well as the single data points are shown (E and F). In order to conserve the samples, the less abundant HPLC peaks were only printed on slides tested with MBL, TEPC-15 or CRP (***Supplementary Figure 21***).

Supplementary Figure 20:

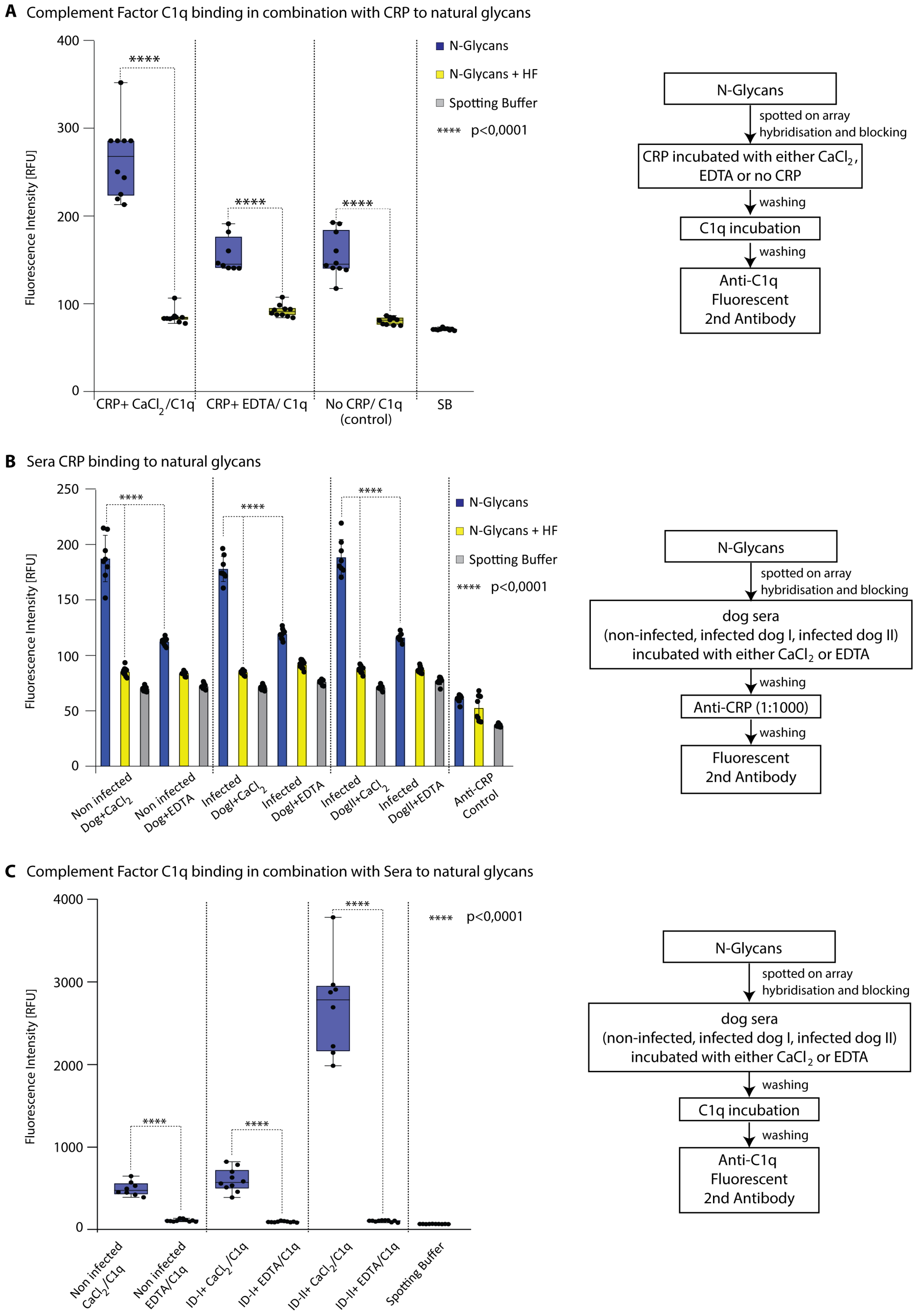

Further data on the interaction of CRP with *Dirofilaria* N-glycans. (A) Box and whisker depiction of binding of human CRP to 'untreated' (no HF) and 'stripped' (+HF) neutral *Dirofilaria* N-glycans (2 fmol per spot; as detected by human C1q and fluorescent anti-C1q in the presence of either 5 mM $CaCl_2$ or EDTA. **(B)** Array data on binding of endogenous C-reactive protein (CRP) in three dog sera to the 'untreated' (no HF) and 'stripped' (+HF) neutral *Dirofilaria* N-glycans; the binding of CRP was assessed using rabbit anti-CRP and a fluorescent goat anti-rabbit IgG. **(C)** Box and whisker depiction of sera- and calcium-dependent binding of human C1q to the 'untreated' neutral *Dirofilaria* N-glycans; this is an extended set of the data shown in ***Figure 10D*** of the main text and includes the EDTA controls and shows higher binding in the case of one infected dog serum. Significance of array data was assessed by a t-test (* indicates p less than 0.05). The flowcharts indicate the steps for each experiment. The reduction in binding to CRP after HF treatment is due to removal of phosphorylcholine residues from the N-glycans.

Supplementary Figure 21:

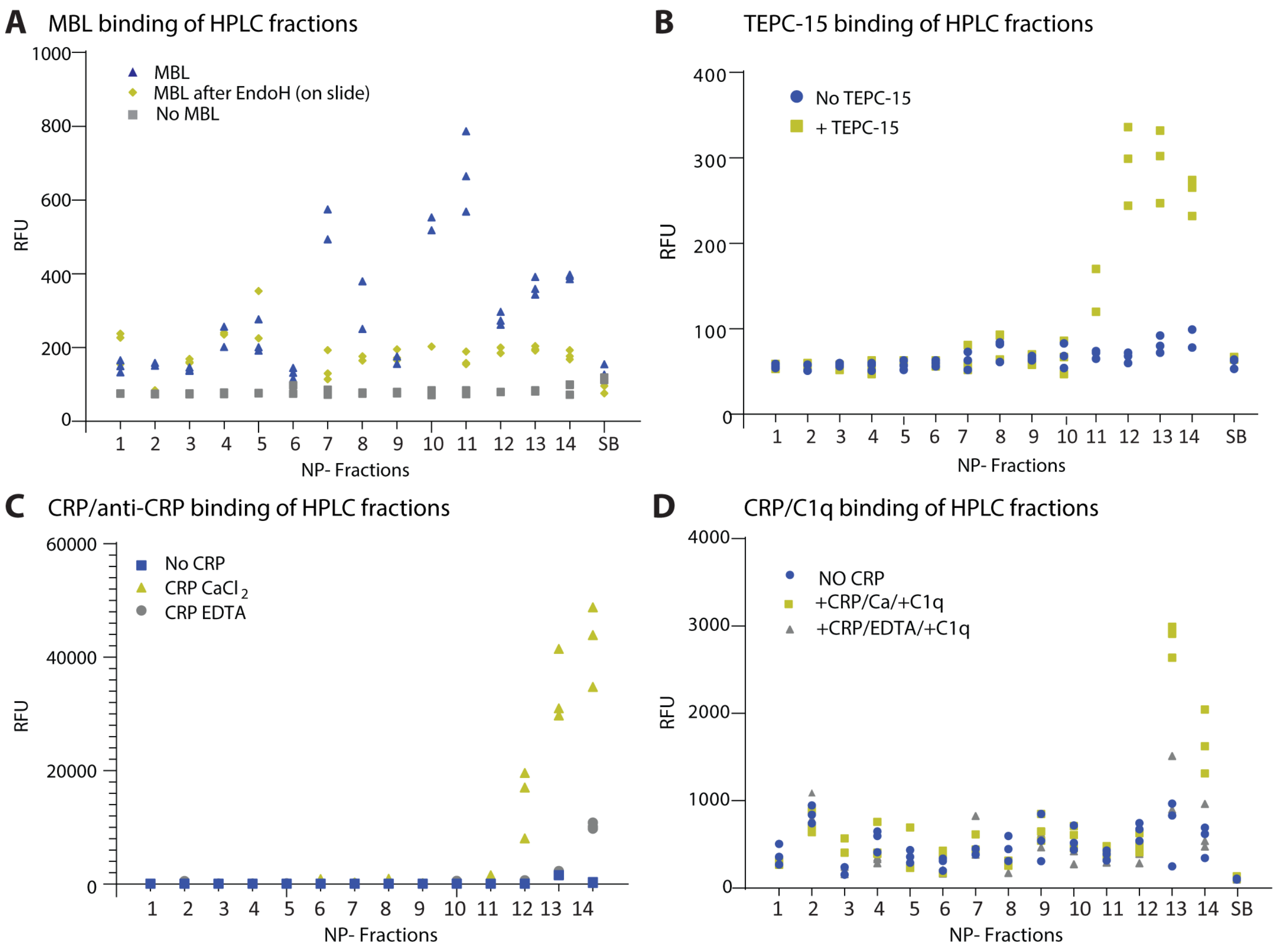

Interaction of fractionated AEAB-labelled N-glycans with a lectin, an antibody and a pentraxin. The fourteen N-glycan fractions shown in ***Supplementary Figure 19A*** were tested for binding towards mannose binding lectin, anti-phosphorylcholine and C-reactive protein. **(A)** Binding of immobilized NP-HPLC fractions (without/with on-slide Endo H treatment; ca. 1 fmol glycan per spot) to human mannose binding lectin (MBL) suggesting Endo H-sensitive binding to fractions containing $Man_{6\text{-}9}GlcNAc_2$ as well as larger N-glycans. **(B-D)** Binding of immobilized NP-HPLC fractions (ca. 1 fmol glycan per spot in triplicate) to TEPC-15 (detected with a fluorescent anti IgA, with no TEPC-15 as a negative control), C-reactive protein in the presence of $CaCl_2$ (detected with anti-CRP, with no CRP or CRP/EDTA as controls) and C-reactive protein in the presence of $CaCl_2$ (detected with C1q/anti-C1q, with no CRP or CRP/EDTA as controls); note that fractions 13 and 14 have the highest binding to both TEPC-15 and CRP, which would correlate with the presence of doubly PC-modified N-glycans. The single data points are shown in all cases.

Supplementary Figure 22:

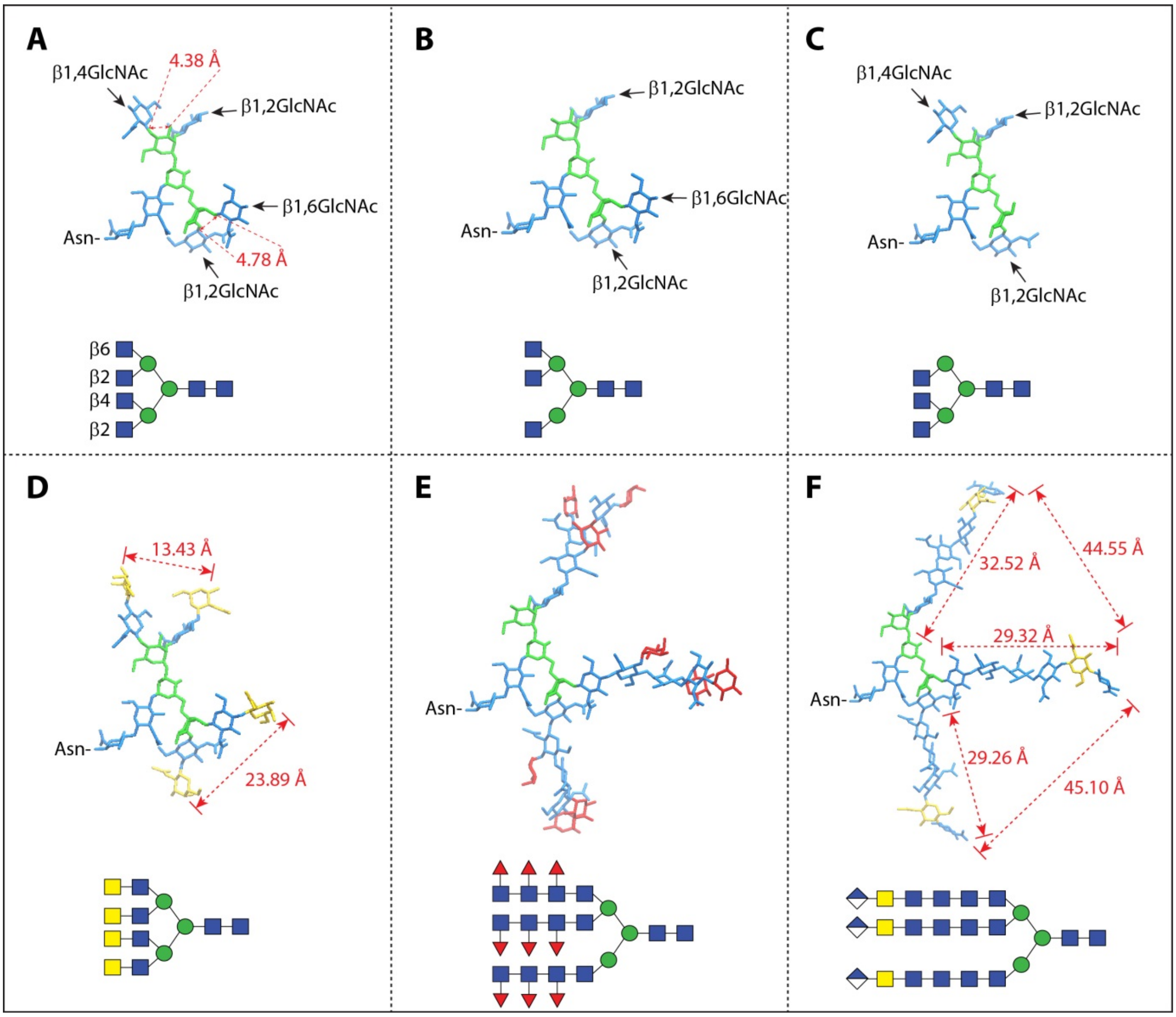

Modelling of selected *D. immitis* glycan structures. 3D models were generated using the Carbohydrate Builder server (http://glycam.org) and visualised using the visual molecular dynamics program (VMD, version 1.9.2) with an optional 3D-SNFG script as described by Thieker *et al. [7]*. Structures at the lowest energy states were chosen for comparison purposes. Branches of glycans are indicated with solid arrows, whereas the distances between residues are indicated in red dashed lines (A, D and F). The distances, either between the oxygen atoms of neighbouring GlcNAc residues (A) or the carbon atoms of furthest non-reducing residues (D and F), were measured using Jmol viewer (version 14.2.12)). Green, Man; blue, GlcNAc or GlcA; red, Fuc; yellow, GalNAc; Asn, asparagine. The theoretical models of the long fucosylated and glucuronylated structures (panels E and F) suggests a type of 'umbrella' conformation akin to synthetic glycodendrimers. Recent data on tetraantennary N-glycans would suggest that a 'gauche-gauche' extended conformation of the Manα1,6Man linkage dominates, as opposed to greater flexibility in biantennary N-glycans, for which Canales *et al* indicated that both 'gauche-gauche' and 'gauche-trans' conformers are populated *[8]*. Note that a distance of 45 Å between the longer antennae (panel F) is similar to that between the phosphorylcholine-binding sites of the pentameric C-reactive protein *[9]* and so may explain the highest binding of this pentraxin to the fractions containing glycans with two phosphorylcholine residues (see ***Supplementary Figure 21***).

Supplementary Table 1: List of theoretical $[M+H]^+$ *m/z* values for all neutral pyridylaminated N-glycans with a verified MS/MS profile; glycan compositions (corresponding often to multiple isomers) are given in the form $H_xN_yF_zPC_{0\text{-}1}$, whereby H refers to hexose (here always mannose, except for *m/z* 1297), N to *N*-acetylhexosamine (GalNAc or GlcNAc), F to fucose and PC to phosphorylcholine. Larger glycans up to 7000 Da are not listed, but refer to ***Supplementary Figures 2 and 10*** for average mass data indicating their occurrence in *D. immitis.*

Composition	[M+H]+
HN2F2	957.4
H2N2F1	973.4
H3N2	989.4
H2N2F2	1119.5
H3N2F1	1135.5
H4N2	1151.4
H3N3	1192.5
H3N2F2	1281.5
H4N2F1	1297.5
H5N2	1313.5
H3N3F1	1338.5
H3N4	1395.6
H4N2F2	1443.6
H5N2F1	1459.6
H6N2	1475.6
H3N3F2	1484.6
H4N3F1	1500.6
H3N4F1	1541.6
H3N4PC	1560.6
H3N5	1598.6
H7N2	1637.6
H3N4F2	1687.6
H4N4F1	1703.7
H3N4F1PC	1706.7
H3N5F1	1744.7
H3N5PC	1763.7
H8N2	1799.7
H3N6	1801.7
H3N4F3	1833.7
H3N4F2PC	1852.7
H3N4F1PC2	1871.7
H3N5F2	1890.7
H3N5F1PC	1909.7
H3N6F1	1947.8
H9N2	1961.7
H3N4F3PC	1998.8
H3N7	2004.8
H3N5F3	2036.8
H3N5F2PC	2055.8
H3N6F2	2093.8
H3N6F1PC	2112.8
H10N2	2123.8
H3N6PC2	2131.8
H3N7F1	2150.8
H3N5F4	2182.9
H3N8	2207.9
H3N6F3	2239.9
H3N6F2PC	2258.9
H3N7F2	2296.9
H3N7F1PC	2315.9
H3N7PC2	2334.9
H3N8F1	2353.9
H3N6F4	2385.9
H3N6F3PC	2404.9
H3N6F2PC2	2423.9
H3N7F3	2442.9
H12N2	2447.9
H3N7F2PC	2462.0
H3N7F1PC2	2481.0
H3N8F2	2499.0
H3N8F1PC	2519.0
H3N6F5	2532.0
H3N9F1	2557.0
H3N7F4	2589.0
H3N7F3PC	2608.0
H3N10	2614.0
H3N8F3	2646.0
H3N8F2PC	2665.0
H3N9F2	2703.1
H3N9F1PC	2722.1
H3N7F5	2735.1
H3N10F1	2760.1
H3N8F4	2792.1
H3N9F3	2849.1
H3N8F5	2938.2
H3N11F1	2963.2
H3N8F6	3084.2
H3N9F5	3141.2
H3N10F4	3198.3
H3N9F6	3287.3
H3N10F5	3344.3
H3N9F7	3433.4
H3N10F6	3490.4
H3N11F5	3547.4
H3N10F7	3636.4
H3N11F6	3693.5
H3N11F7	3839.5
H3N11F8	3985.6
H3N12F7	4042.6

Supplementary Table 2: List of theoretical *m/z* values (as $[M+H]^+$ and $[M-H]^-$) for all glucuronylated pyridylaminated glycans with a verified MS/MS profile; glycan compositions (corresponding often to multiple isomers) are given in the form $H_3N_xF_yU_zPC_{0\text{-}1}$, whereby H refers to hexose (here always mannose), N to *N*-acetylhexosamine (GalNAc or GlcNAc), F to fucose, U to glucuronic acid and PC to phosphorylcholine. Larger glucuronylated glycans up to 5000 Da are not listed, but refer to ***Supplementary Figures 2 and 11*** for mass spectral data indicating their occurrence in *D. immitis.*

Composition	[M+H]+	[M-H]-
H3N4U1	1571.6	1569.6
H3N4F1U1	1717.6	1715.6
H4N4U1	1733.6	1731.6
H3N5U1	1774.7	1772.6
H3N4F2U1	1863.7	1861.7
H3N4F1U1PC	1882.7	1880.7
H5N4U1	1895.7	1893.7
H3N5F1U1	1920.7	1918.7
H3N6U1	1977.7	1975.7
H3N5F2U1	2066.8	2064.8
H3N6F1U1	2123.8	2121.8
H3N6U2	2153.8	2151.8
H3N7U1	2180.8	2178.8
H3N6F2U1	2269.9	2267.8
H4N6F1U1	2285.9	2283.8
H3N6F1U1PC	2288.9	2286.8
H3N6F1U2	2299.8	2297.8
H3N7F1U1	2326.9	2324.9
H3N7U2	2356.9	2354.8
H3N8U1	2383.9	2381.9
H3N6F2U1PC	2434.9	2432.9
H3N7F2U1	2472.9	2470.9
H4N7F1U1	2488.9	2486.9
H3N7F1U1PC	2491.4	2489.9
H3N7F1U2	2501.9	2500.9
H3N8F1U1	2530.0	2527.9
H3N8U2	2559.9	2557.9
H3N7F3U1	2619.0	2617.0
H3N7F2U1PC	2638.0	2636.0
H3N7F2U2	2649.1	2647.1
H3N8F2U1	2676.0	2674.0
H3N8F1U1PC	2695.0	2693.0
H3N8F1U2	2706.0	2704.0
H3N9F1U1	2733.0	2731.0
H3N8U3	2736.0	2734.1
H3N9U1PC	2752.0	2750.0
H3N10U1	2790.1	2788.1
H3N8F2U1PC	2841.1	2839.1
H3N8F1U2PC	2871.0	2869.0
H3N9F2U1	2879.1	2877.1
H3N8F1U3	2882.0	2880.0
H3N9F1U1PC	2898.1	2896.1
H3N9F1U2	2909.1	2907.1
H3N10F1U1	2936.1	2934.1
H3N9U3	2939.0	2937.0
H3N9F1U2PC	3074.1	3072.1
H3N10F2U1	3082.2	3080.2
H3N9F1U3	3085.1	3083.1
H3N10F1U1PC	3101.2	3099.2
H3N10F1U2	3112.2	3110.1
H3N11F1U1	3139.2	3137.2
H3N10F1U3	3288.2	2386.2
H3N11F1U2	3315.2	3313.2
H3N12F1U1	3342.3	3340.3
H3N12F1U2	3518.3	3516.3
H3N11U4	3521.2	3519.2
H3N11F1U4	3667.3	3665.3
H3N12U4	3724.3	3722.3
H3N12UF1U4	3870.4	3868.4

Supplementary Table 3: Listing of example *D. immitis* N-glycans with $[M+H]^+$ *m/z* values rounded to the nearest integer; the structures are arranged by category and have been verified by two or more means: i.e., retention time comparisons to glycans from other organisms, MALDI-TOF MS/MS and chemical/enzymatic treatments. Not listed are glycans for which only compositions can be determined, but for which long fucosylated/PC-modified chitobiose chains are assumed.

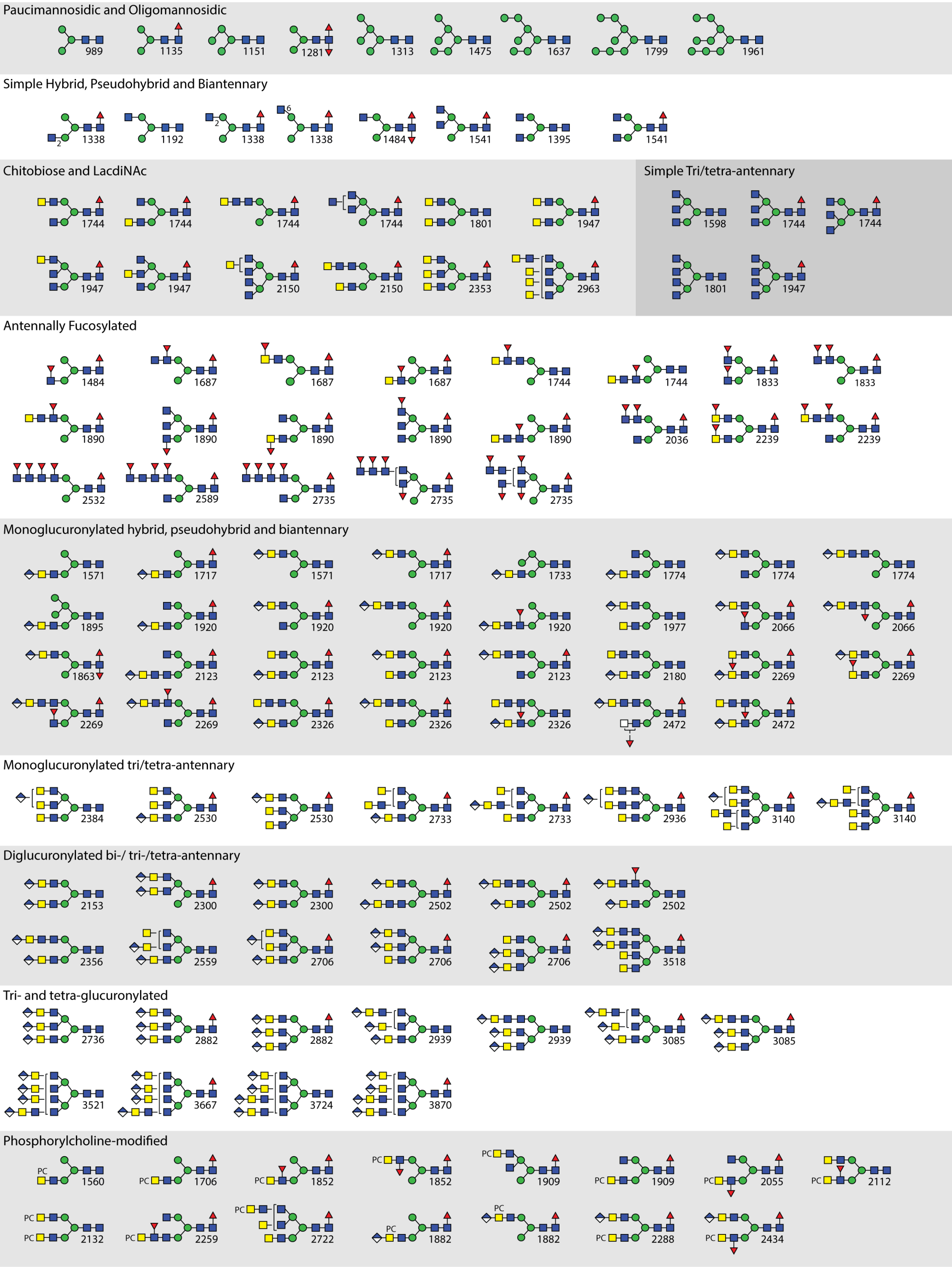

Supplementary Methods:

Mass spectrometric analyses of proteins in the parasite extract:

D. immitis glycoproteins were enriched by affinity chromatography with either CGL3 (lectin) or TEPC-15 (antibody) coupled to CNBr-activated Sepharose 4B (GE Healthcare 17043001), prepared following the manufacturer's protocol. In brief, *D. immitis* extract (homogenised in sample buffer, i.e., 20 mM MES, pH 6.7) was loaded onto 150 µl pre-inactivated CNBr-activated Sepharose (pre-equilibrated with 3 x 500 µl of sample buffer) and rotated for 2h at 4°C. The flow-through was collected and then applied to 150 µl pre-equilibrated CGL3- or TEPC-15-Sepharose and rotated overnight at 4°C. The flow through and wash (6 x 500 µL) were collected and the bound glycoproteins eluted with sample buffer supplemented with 6 M urea (incubation for 15 min at 37°C). For Filter Aided Sample Preparation (FASP), samples (either bound glycoproteins or an unfractionated nematode extract) were subject to buffer exchange with freshly-prepared 50 mM NH_4CO_3, pH 8 (ABC buffer) using a Microcon YM-30 (Millipore, Cat MRCF0R030) 30000 MWCO centrifugal filter unit. Samples were reduced on the filter device using 50 mM dithiothreitol in ABC buffer for 1 h at 37°C, washed 3 times with ABC, alkylated with 65 mM iodoacetamide in ABC for 1h at 37°C, washed 3 times with ABC and incubated overnight at 37°C with trypsin in ABC buffer. The (glyco)peptides were then collected by centrifugation and washing the device with water.

Tryptic peptides were analyzed on a calibrated LTQ-Orbitrap Velos mass spectrometer (Thermo Fisher Scientific, Bremen, Germany) coupled to an Eksigent-Nano-HPLC system (Eksigent Technologies, Dublin (CA), USA). Peptides were resuspended in 2.5% acetonitrile and 0.1% formic acid and loaded on a self-made tip column (75 µm × 80 mm) packed with reverse phase C18 material (AQ, 3 µm 200 Å, Bischoff GmbH, Leonberg, Germany) and eluted with a flow rate of 200 nl per min and a gradient from 3 to 30% of B in 22 min, 50% B in 25 min, 97% B in 27 min. One scan cycle comprised of a full scan MS survey spectrum, followed by up to 20 sequential CID MS/MS on the most intense signals above a threshold of 1500. Full-scan MS spectra (300–2000 *m/z*) were acquired in the FT-Orbitrap at a resolution of 60,000 at 400 *m/z*, while HCD MS/MS spectra were recorded in the Orbitrap. HCD was performed with a target value of $5e^4$ in the HCD cell and collision energy at 35 V. AGC target values were $5e^5$ for full FTMS scans. For all experiments, dynamic exclusion was used with 1 repeat count, 15 s repeat duration, and 60 s exclusion duration. MS/MS validation was performed by Scaffold 3.6.2 (Proteome Software Inc, USA).

MS and MS/MS data were processed into Mascot generic format (mgfs) and the monoisotopic masses of doubly or more charged peptides were searched with Mascot (version 2.4; parameters being carbamidomethylation at cysteine, oxidation at methionine, maximally one missed trypsin cleavage, a peptide tolerance of 10 ppm and a MS/MS tolerance of 0.1 Da) against both the *D. immitis* nDi.2.2 (https://parasite.wormbase.org/Dirofilaria_immitis_prjeb1797/Info/Index/) and *Canis lupus familiaris* (http://www.uniprot.org/proteomes/UP000002254) proteome databases. Refer to ***Supplementary Figure 17*** for the resulting data.

Fucosylation of chitobiose or LacdiNAc.

The 6-(5-aminopentan-amido)-*N*-(2-[2-[disaccharyl-*N*-methoxyamino]ethoxy)ethyl]-2-naphthamide forms of chitobiose and LacdiNAc were prepared as follows: linker and disaccharide were dissolved in dioxane/ammonium acetate buffer 1:1 (v:v) pH 4.6 and the reaction mixture was stirred at 40 °C for 72 h; the crude reaction was then concentrated under reduced pressure and purified by silica gel column chromatography *[10]*. Either conjugate (final concentration of 10 µM) was mixed with 20 mM $MnCl_2$, 80 mM MES buffer pH 6.5, 2 mM GDP-Fuc, concentrated *C. elegans* FUT-6 *[11]* and water (final volume 2.5 µl) and left overnight at room temperature. The reaction was followed by MALDI-TOF as well as NP-HPLC. Aliquots of the non-fucosylated or fucosylated forms were also incubated with *N*-acetylhexosaminidases to assess their sensitivity to either a chitinase, *C. elegans* GalNAc-specific HEX-4 or non-specific jack bean hexosaminidase. Thereby, only LacdiNAc was sensitive to HEX-4, but both chitobiose and LacdiNAc were chitinase sensitive (see MALDI-TOF MS data); also both fucosylated forms lost a single HexNAc upon jack bean hexosaminidase treatment (JB-HN), indicative that the fucose is on the non-reducing GlcNAc of both conjugates (not shown).

Supplementary Figure 23: Hexosaminidase digestion or fucosylation of chitobiose and LacdiNAc conjugates; a '+' refers to sensitivity and a '-' to resistance.

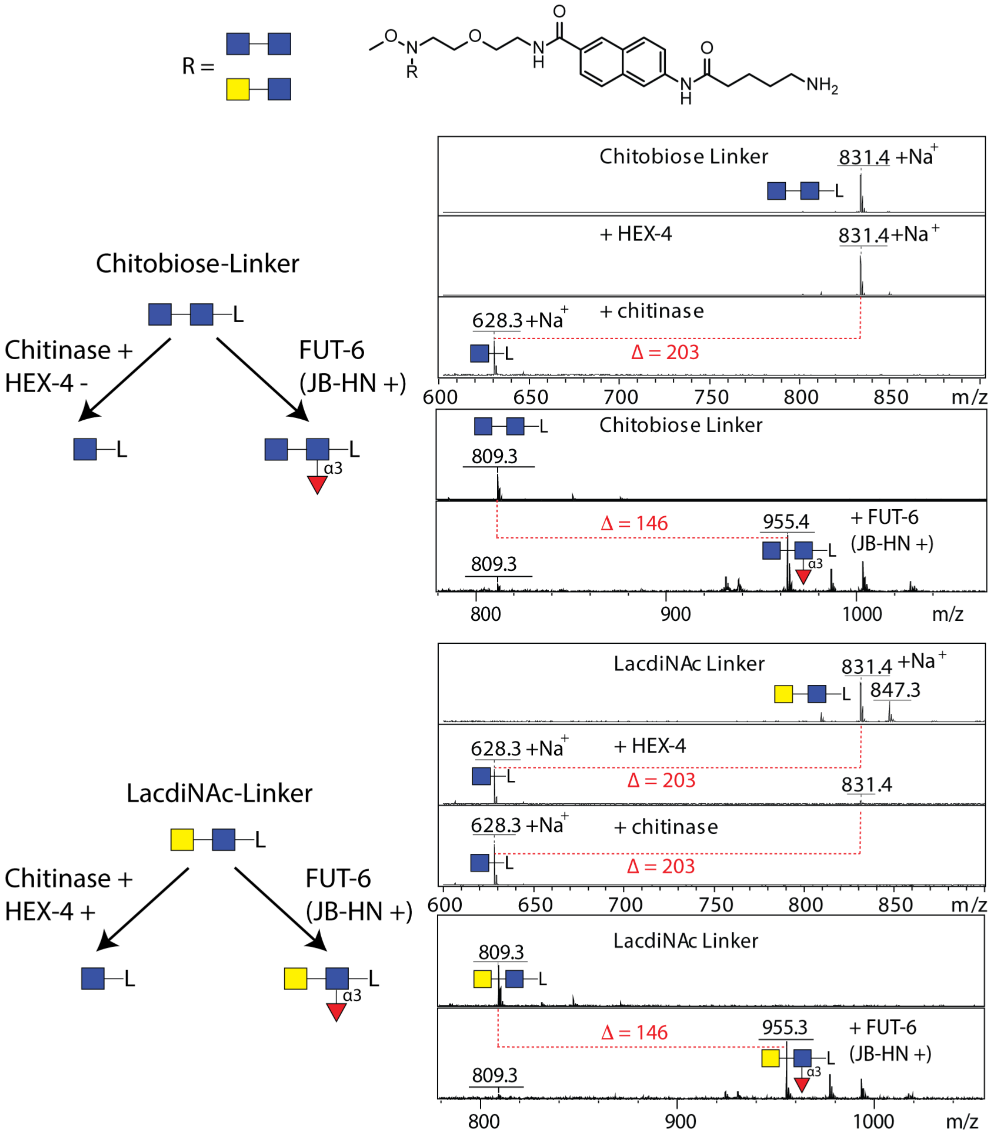

Synthesis of LacdiNAc (as control for enzyme and array assays)

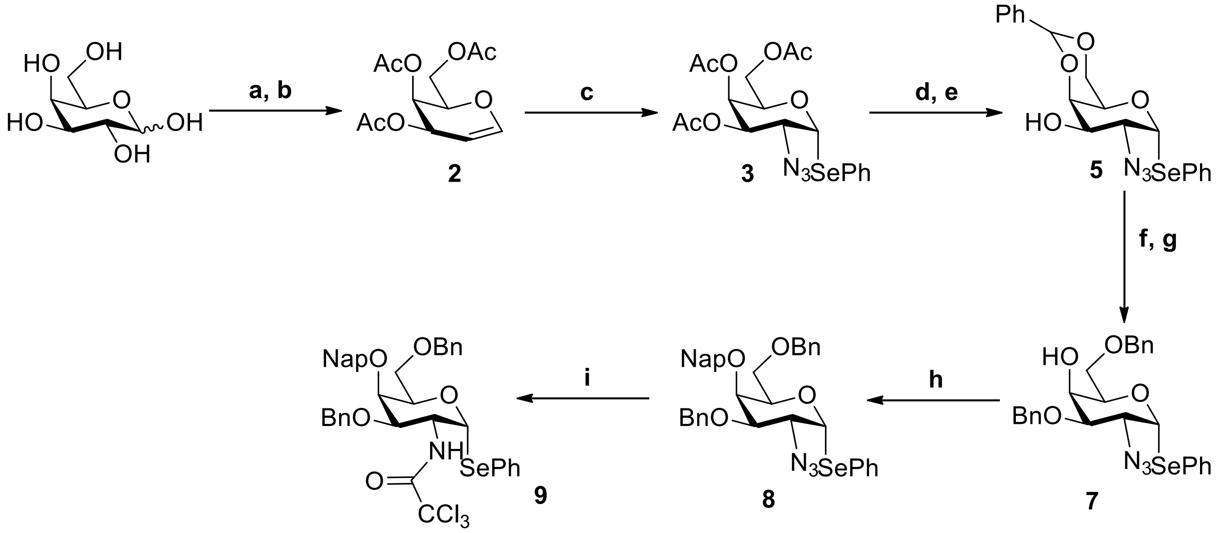

Supplementary Figure 24. Conditions: a) Acetic anhydride, Pyridine, 0-25 °C, 14 h; b) HBr (33 %), DCE, 0-25 °C, 2 h, then $CuSO_4$, sodium acetate, zinc, acetic acid, H_2O, rt, 65 % (over 2 steps); c) Diphenyldiselenide, NaN_3, iodobenzenediacetate, DCM, rt, 48 h, 60 %; d) Sodium methoxide, MeOH, rt, 30 min, 95 %; e) Benzaldehyde dimethyl acetal, camphorsulphonic acid, ACN, rt, 2 h, 91 %; f) NaH, BnBr, DMF, 0-25 °C, 2 h, 73 %; g) Trifluoroacetic anhydride (TFAA), triethylsilane (TES), Trifluoroacetic acid (TFA), 0-25 °C, 5 h, 88 %; h) NaH, 2-(bromomethyl)naphthalene, DMF, 0-25 °C, 2 h, 60 %; i) Zinc, Acetic acid, 2 h, then pyridine, 2,2,2-trichloroacetyl chloride, 0-25 °C, 14 h, 87 %.

3,4,6-*O*-acetyl-2-deoxy-α-D-galactal (2). To a stirred solution of galactose (40 g, 222 mmol) in pyridine (250 mL) acetic anhydride (150 mL) was added at 0 °C yielding peracetylated galactose (83 g, 95 %). Without any purification, to a solution of peracetylated galactose (11.3 g, 28.9 mmol) in DCE (110 ml) hydrobromic acid (47 mL of 33 %, 289 mmol) was added at 0 °C. After 2 h, the volatiles were evaporated from the reaction mixture. The residue was dissolved in acetic acid (50 ml) and water (50 mL) followed by the addition of $CuSO_4$ (1.85 g, 11.58 mmol), sodium acetate (28.5 g, 347 mmol). Next, zinc (22.71 g, 347 mmol) was added portion wise at 0 °C and the reaction mixture was stirred at room temperature overnight. The reaction mixture was filtered through celite and extracted using DCM. Organic layer was washed with $NaHCO_3$ and brine solution, dried over anhy. Na_2SO_4, filtered and concentrated. The crude product was purified using silica gel column chromatography to give **2** (5 g, 65 % yield) over 2-steps in quantitative yield. Spectral data were in agreement with the data previously reported by Czernecki *[12].*

Phenyl 2-azido-3,4,6-*O*-acetyl-2-deoxy-1-seleno-α-D-galactopyranoside (3). To a stirred solution of galactal **2** (2.9 g, 10.65 mmol) in DCM diphenyldiselenide (5.82 g, 18.64 mmol), sodium azide (NaN_3) (3.46 g, 53.30 mmol) and iodobenzenediacetate (7.55 g, 23.43 mmol) were added under an argon atmosphere. The reaction mixture was stirred at room temperature until the TLC showed the completion of the reaction. Then, the reaction mixture was diluted with DCM and extracted using a saturated solution of $NaHCO_3$ and brine. The organic layer was dried over Na_2SO_4, filtered and concentrated. The crude product was purified using silica gel column chromatography to give galactoside **3** (3 g, 60 % yield) as a white solid. Spectral data was in agreement with the previous reported data *[12].*

Phenyl 2-azido-2-deoxy-1-seleno-α-D-galactopyranoside (4). To a stirred solution of galactoside **3** (1.5 g 2.5 mmol), in methanol was added freshly prepared sodium methoxide. After 30 min, the reaction mixture was neutralized using amberlite resin (H^+-form). The reaction mixture was filtered through sintered funnel to give compound **4** (1.1 g, 95 % yield).

Phenyl 2-azido-4,6-*O*-benzylidene-2-deoxy-1-seleno-α-D-galactopyranoside (5). To a stirred solution of **4** (1.1 g, 2.37 mmol) in acetonitrile (15 mL) was added benzaldehyde dimethyl acetal (0.712 mL, 4.75 mmol) followed by the addition of camphorsulphonic acid (0.045 g, 0.237 mmol) at room temperature under argon atmosphere. After 2 h, the reaction was quenched using triethylamine and volatiles were removed using rotavapour. The residue was dissolved in the DCM and washed with saturated $NaHCO_3$ and brine. The organic layer was dried using Na_2SO_4, filtered and concentrated. The crude product was purified using silica gel column chromatography to give **5** (1.2 g, 91 % yield). Spectral data was in agreement with the previous reported data *[12].*

Phenyl 2-azido-3-*O*-benzyl-4,6-*O*-benzylidene-2-deoxy-1-seleno-α-D-galactopyranoside (6). To a stirred solution of benzylidene **5** (1.2 g, 2.14 mmol) in anhy. dimethylformamide (10 mL), sodium hydride (0.08 g, 3.21 mmol) was added under argon atmosphere at 0 °C. After 30 min, benzyl bromide (0.3 mL, 2.57 mmol) was added dropwise and the reaction was stirred at room temperature. After 2 h, the reaction mixture was quenched using methanol and volatiles were removed using rotavapour. The residue was dissolved in DCM and the reaction mixture was neutralized with 1N HCl (aqueous) and extracted three times with DCM. The combined organic layers were dried over Na_2SO_4, filtered and concentrated. The crude product was purified using silica gel column chromatography to give **6** (1 g, 73 % yield) as a white solid. Spectral data was in agreement with the previous reported data *[12].*

Phenyl 2-azido-3,6-di-*O*-benzyl-2-deoxy-1-seleno-α-D-galactopyranoside (7). To a stirred solution of galactoside **6** (0.956 g, 1.83 mmol) in DCM (10 mL) at 0 °C, trifluoroacetic anhydride (1 mL, 7.32 mmol), triethylsilane (2 mL, 12.81 mmol) trifluoroacetic acid (1 mL, 12.81 mmol) were added dropwise. The reaction was stirred for 5 h at 0 °C. Then, Et_3N was added and the reaction mixture was filtered. The filtrate was concentrated to give a yellow oil that was purified by silica gel column chromatography to give galactoside **7** (0.845 g, 88 % yield) as a colorless oil[2]. Rf 0.33 (Hexanes/EtOAc = 3 : 1); 1H NMR (400 MHz, CDCl3) δ 7.53-7.51 (m, 2H), 7.34-7.10 (m, 13H), 5.85 (d, *J* = 5.4 Hz, 1H, H1), 4.66 (d, *J* = 11.6 Hz, 1H, -CH2-), 4.63 (d, *J* = 11.6 Hz, 1H, -CH2-), 4.45 (d, *J* = 11.8 Hz, 1H, -CH2-), 4.41 (d, *J* = 11.8 Hz, 1H, -CH2-), 4.32 (br-t, *J* = 5.5 Hz, 1H, H5), 4.16 (dd, *J* = 5.4, 10.2 Hz, 1H, H2), 4.08 (br, H4), 3.66 (dd, *J* = 5.4, 10.2 Hz, 1H, H6), 3.61-3.56 (m, 2H, H3 & H6); 13C NMR (75 MHz, CDCl3) δ 137.76, 136.99, 134.77, 129.06, 128.69, 128.43, 128.30, 128.12, 128.04, 127.92, 127.78, 127.69, 85.22 (C1), 78.85 (C3), 73.57 (-CH2-), 71.99 (-CH2-), 71.23 (C5), 69.30 (C6), 66.39 (C4), 60.25 (C2).

Phenyl 2-azido-3,6-di-*O*-benzyl-2-deoxy-4-*O*-(2-naphthylmethyl)-1-seleno-α-D-galactopyranoside (8). To a stirred solution of galactoside **7** (0.845 g, 1.61 mmol) in DMF (10 mL), 2-(bromomethyl)naphthalene (0.534 g, 2.42 mmol) was added. After 30 min, the reaction was cooled to 0°C and sodium hydride (0.077 g, 3.22 mmol) was added. After 2 h, the reaction was quenched using methanol, neutralized with 1N HCl $_{(aq)}$ and extracted three times with dichloromethane. The combined organic layers were dried over Na_2SO_4, filtered and concentrated. The crude product was purified by silica gel column chromatography to give galatoside **8** (0.635 g, 60 % yield) as a white solid *[13]*: Rf 0.25 (Hexanes/EtOAc = 9 : 1); 1H NMR (400 MHz, $CDCl_3$) δ 7.70-7.64 (m, 3H), 7.56 (br, 1H), 7.50-7.49 (m, 2H), 7.37-7.07 (m, 16H), 5.85 (d, *J* = 5.3 Hz, 1H, H1), 4.94 (d, *J* = 11.6 Hz, 1H, -CH2-), 4.64 (s, 2H, -CH2-*2), 4.61 (d, *J* = 11.6 Hz, 1H, -CH2-), 4.33-4.30 (m, 2H, H2 & H5), 4.29 (d, *J* = 11.7 Hz, 1H, -CH2-), 4.24 (d, *J* = 11.7 Hz, 1H, -CH2-), 3.99 (br, H4), 3.64 (d, *J* = 2.6, 10.6 Hz, 1H, H3), 3.53 (dd, *J* = 7.2, 9.3 Hz, 1H, H6), 3.37 (dd, *J* = 5.9, 9.3 Hz, 1H, H6); 13C NMR (75 MHz, CDCl3) δ 137.80, 137.43, 135.65, 134.77, 133.20, 133.03, 129.06, 128.63, 128.45, 128.38, 128.14, 128.07, 127.94, 127.88, 127.85, 127.82, 127.73, 126.80, 126.18, 126.13, 125.98, 85.55 (C1), 80.33 (C3), 74.95 (-CH2-), 73.44 (-CH2-), 72.97 (C4), 72.49 (-CH2-), 71.97 (C5), 68.35 (C6), 61.11 (C2).

Phenyl-3,6-di-*O*-benzyl-2-deoxy-4-*O*-(2-naphthylmethyl)-1-seleno-2-trichloracetamido-α-D-galactopyranoside (9). To a stirred solution of galatoside **8** (0.620 g, 0.93 mmol) in DCM (5 ml),

acetic acid (5 mL) was added followed by the addition of zinc (1.2 g, 18.66 mmol). The reaction mixture was stirred for 2 h, then it was filtered through celite, neutralised using sat. $NaHCO_3$ and extracted three times with dichloromethane. The combined organic layers were dried over Na_2SO_4, filtered and concentrated to give a white solid. The residue (0.590 g, 0.92 mmol) was re-dissolved in dichloromethane (10 mL). Pyridine (1 mL) and 2,2,2-trichloroacetyl chloride (0.207 mL, 1.84 mmol) was added to this solution at 0 °C. After 30 min, the solvent was removed and the residue was recrystallized from MeOH to give galactoside **9** (0.630 g, 87 % yield) as a white solid *[13]*. Rf 0.38 (Hexanes/EtOAc = 4 : 1); 1H NMR (400 MHz, $CDCl_3$) δ 7.72-7.68 (m, 3H), 7.63 (br, 1H), 7.39-7.38 (m, 3H), 7.45-7.44 (m, 2H), 7.40-7.37 (m, 3H), 7.30-7.10 (m, 13H), 6.73 (d, *J* = 7.1 Hz, 1H, NH), 6.03 (d, *J* = 4.8 Hz, 1H, H1), 4.99 (d, *J* = 11.6 Hz, 1H, -CH2-), 4.70 (m, 1H, H2), 4.69 (d, *J* = 11.6 Hz, 1H, -CH2-), 4.62 (d, *J* = 12.0 Hz, 1H, -CH2-), 4.41 (d, *J* = 12.0 Hz, 1H, -CH2-), 4.38 (d, *J* = 11.6 Hz, 1H, -CH2-), 4.32 (d, *J* = 11.6 Hz, 1H, -CH2-), 4.28 (br-t, *J* = 6.6 Hz, H5), 4.11 (br, H4), 3.66 (dd, *J* = 7.5, 9.2 Hz, 1H, H6), 3.54-3.50 (m, H3 & H6); 13C NMR (75 MHz, $CDCl_3$) δ 161.57 (amide), 137.74, 137.06, 135.52, 134.36, 133.18, 133.04, 129.26, 128.81, 128.80, 128.49, 128.48, 128.26, 128.19, 128.04, 127.96, 127.92, 127.91, 127.90, 127.88, 127.86, 127.84, 127.71, 126.84, 126.23, 126.10, 125.95, 92.44 (CCl3), 88.69 (C1), 77.99 (C3), 74.74 (-CH2-), 73.58 (-CH2-), 72.81 (C5), 71.64 C4), 71.33 (-CH2-), 68.30 (C6), 52.20 (C2); ESI-MS: m/z [M+Na]+ cald 806.0722, obsd 806.0733.

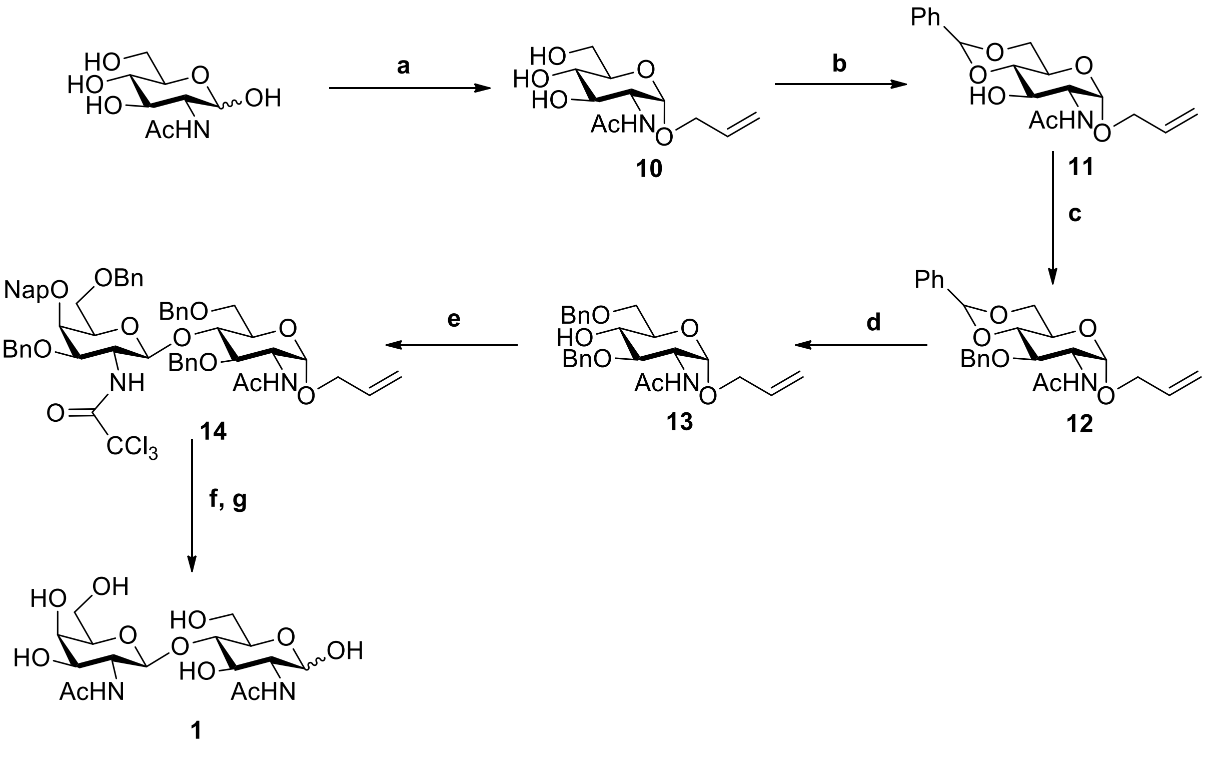

Supplementary Figure 25. Conditions: a) $BF_3.OEt_2$, Allyl alcohol, rt, 14 h, 85 %; b) Benzaldehyde dimethyl acetal, camphorsulfonic acid, ACN, rt, 2 h, 80 %; c) NaH, BnBr, DMF, 0-25 °C, 2 h, 55 %; d) Trifluoroacetic anhydride (TFAA), triethylsilane (TES), Trifluoroacetic acid (TFA), 0-25 °C, 2 h, 79 %; e) **9**, NIS, TMS-OTf, 0 °C, 2 h, 65 %; f) $PdCl_2$, sodium acetate, Acetic acid, H_2O, rt, 3-4 h, 71 %; g) Pd-C, H_2, $CHCl_3$: MeOH (4:1), formic acid, 72 h, 63 %.

Allyl-2-acetamido-2-deoxy-α-D-glucopyranoside (10). To a stirred solution of *N*-acetylgluco-samine (0.650 g, 2.94 mmol) in allyl alcohol (10 mL), BF_3. OEt_2 (1.5 mL, 5.88 mmol) was added at room temperature under argon atmosphere. After 14 h, the reaction mixture was quenched using Et_3N. The volatiles were evaporated and the product was crystallized using methanol and hexane

to give **10** (0.655 g, 85 %) as a white solid. Spectral data was in agreement with the data previously reported by Feng *et al. [14].*

Allyl 2-acetamido-4,6-*O*-benzylidene-2-deoxy-α-D-glucopyranoside (11). To a stirred solution of **10** (0.768 g, 2.94 mmol) in acetonitrile (15 mL) was added benzaldehyde dimethyl acetal (1.1 mL, 7.35 mmol) followed by the addition of camphorsulphonic acid (0.205 g, 0.88 mmol) at room temperature under argon atmosphere. After 2 h, the reaction was quenched using triethylamine and volatiles were removed using rotavapour. The residue was dissolved in the DCM and washed with sat. $NaHCO_3$ and brine. The organic layer was dried using Na_2SO_4, filtered and concentrated. The crude product was purified using silica gel column chromatography to give **11** (0.820 g, 80 % yield). Spectral data was in agreement with the previous reported data *[14].*

Allyl 2-acetamido-3-*O*-benzyl-4,6-*O*-benzylidene-2-deoxy-α-D-glucopyranoside (12). To a stirred solution of benzylidene **11** (1.0 g, 2.92 mmol) in anhydrous dimethylformamide (15 mL), sodium hydride (0.08 g, 3.21 mmol) was added under argon atmosphere at 0 °C. After 30 min, benzyl bromide (0.38 mL, 3.21 mmol) was added dropwise and the reaction was stirred at room temperature. After 2 h, the reaction mixture was quenched using methanol and volatiles were removed using rotavapour. The residue was dissolved in DCM and the reaction mixture was neutralized with 1N aq. HCl and extracted three times with DCM. The combined organic layers were dried over Na_2SO_4, filtered and concentrated. The crude product was purified using silica gel column chromatography to give **12** (0.715 g, 55 % yield) as a white solid. Spectral data was in agreement with the previous reported data *[14].*

Allyl 2-acetamido-3,6-di-*O*-benzyl-2-deoxy-α-D-glucopyranoside (13). To a stirred solution of glucoside **12** (0.08 g, 0.19 mmol) in DCM (3 mL), trifluoroacetic anhydride (0.08 mL, 0.57 mmol) and triethylsilane (0.15 mL, 0.94 mmol) were added at 0 °C followed by the addition of trifluoroacetic acid (0.07 mL, 0.94 mmol) drop wise. After 5 h at 0 °C, Et_3N was added and the reaction mixture was filtered. The filtrate was concentrated to give a yellow oil that was purified by silica gel column chromatography to give galactoside **13** (0.066 g, 79 % yield) as a colorless oil. Spectral data was in agreement with the previous reported data *[14].*

^{1}H NMR (400 MHz, $CDCl_3$) δ 7.33 (s, 10H), 5.87 (m, 1H), 5.49 (d, *J* = 9.2 Hz, 1H), 5.22 (dd, *J* = 22.4, 13.7 Hz, 2H), 4.83 (d, *J* = 3.4 Hz, 1H), 4.77 (d, *J* = 11.7 Hz, 1H), 4.69 (d, *J* = 11.8 Hz, 1H), 4.58 (q, *J* = 12.0 Hz, 2H), 4.25 (td, *J* = 10.2, 3.4 Hz, 1H), 4.16 (dd, *J* = 12.9, 4.8 Hz, 1H), 3.95 (dd, *J* = 12.9, 6.1 Hz, 1H), 3.75 (dd, *J* = 19.1, 6.4 Hz, 4H), 1.89 (s, 3H). ^{13}C NMR (101 MHz, $CDCl_3$) δ 170.00, 138.60, 137.91, 133.69, 128.61(2), 128.51(2), 128.12(2), 127.91, 127.83, 127.72(2), 117.77, 96.94, 79.93, 73.93, 73.72, 72.14, 70.42, 70.21, 68.25, 51.91, 23.51.

Allyl-3,6-di-*O*-benzyl-2-deoxy-4-*O*-(2-naphthylmethyl)-2-trichloracetamido-α-D-galactopyranos-ide-β(1→4)-2-acetamido-3,6-di-*O*-benzyl-2-deoxy-α-D-glucopyranoside (14). Compounds **13** (0.066 g, 0.149 mmol) and **9** (0.141 g, 0.179 mmol) were dissolved in toluene, co-evaporated using toluene (5 mL each) three times and dried under high vacuum overnight. Next morning, N-hydroxysuccinimide (NIS) was added to a stirred mixture of acceptor **13** and donor **9** in DCM at 0 °C. After 20 min, TMS-OTf was added at 0 °C to a reaction mixture. After 1 h, reaction was quenched using saturated solution of $NaHCO_3$ and extracted using DCM two times. The organic layer was dried over Na_2SO_4, filtered and concentrated. The crude product was purified by silica gel chromatography to give disaccharide **14** (0.105 g, 65 % yield). $[\alpha]_D^{20}$ = +89.42 (c = 0.15, $CHCl_3$); ATR-FTIR (cm^{-1}): 3287.27, 3066.62, 2927.93, 1777.86, 1710.48, 1654.24, 1526.98, 1455.75, 1362.97, 1292.19, 1173.90, 1103.45, 1060.38, 821.06, 739.07, 699.21. ^{1}H NMR (400 MHz, $CDCl_3$) δ 7.76 (d, *J* = 7.8 Hz, 1H), 7.71 – 7.57 (m, 3H), 7.12 (dd, *J* = 5.6, 3.6 Hz, 23H), 6.64 (d, *J* = 8.1 Hz, 1H), 5.82 (m, 1H), 5.36 (d, *J* = 9.0 Hz, 1H), 5.23 (d, *J* = 1.5 Hz, 1H), 5.20 – 5.12 (m, 1H), 5.00 (d, *J* = 11.5 Hz, 1H), 4.91 (d, *J* = 11.7 Hz, 1H), 4.86 (d, *J* = 3.7 Hz, 1H), 4.73 – 4.59 (m, 4H), 4.55 (d, *J* = 11.8 Hz, 1H), 4.48 (s, 1H), 4.45 (d, *J* = 2.0 Hz, 1H), 4.35 (d, *J* = 11.7 Hz, 1H), 4.26 (d, *J* = 11.7 Hz, 1H), 4.21 (ddd, *J* = 10.7, 9.1, 3.7 Hz, 1H), 4.17 – 4.09 (m, 2H), 4.09 – 3.99 (m, 3H), 3.93 (dd, *J* = 13.0, 6.2 Hz,

1H), 3.82 (dd, *J* = 11.1, 2.6 Hz, 1H), 3.69 – 3.63 (m, 2H), 3.61 – 3.54 (m, 2H), 3.51 – 3.47 (m, 1H), 3.43 (dd, *J* = 12.4, 5.6 Hz, 2H), 1.87 (s, 3H).^{13}C NMR (101 MHz, $CDCl_3$) δ 177.17, 169.78, 161.75, 139.12, 138.14, 137.77, 137.45, 135.99, 133.58, 133.08, 132.83, 128.54, 128.41, 128.36, 128.07, 128.06, 127.98, 127.93, 127.89, 127.82, 127.77, 127.59, 127.10, 126.25, 125.99, 125.91, 125.72, 117.66, 98.48, 96.54, 74.92, 74.57, 73.70, 73.45, 73.06, 72.92, 71.73, 71.67, 70.50, 68.23, 67.83, 55.78, 51.98, 23.36. ESI-MS: m/z $[M+Na]^+$ cald 1089.3233 , obsd 1089.3208.

2-acetamido-2-deoxy-α-D-galactopyranoside-β(1→4)-2-acetamido-2-deoxy-α-D-glucopyranoside (1). The disaccharide **14** (65 mg, 0.061 mmol) and sodium acetate (5 mg, 0.061 mmol) were dissolved in a mixture of acetic acid (1 mL) and MeOH (3 mL). $PdCl_2$ (10 mg, 0.061 mmol) was added, and the reaction mixture was stirred for 5 h. The reaction mixture was filtered through Celite® and concentrated *in vacuo*. Then, the reaction mixture was poured into saturated aq. $NaHCO_3$, and the aqueous layer was extracted with ethyl acetate. The organic layers were combined and dried over Na_2SO_4, filtered and concentrated. The residue was purified by flash column chromatography yielding the hemiacetal in 71% yield. Next, the deallylated disaccharide was dissolved in a mixture of MeOH and $CHCl_3$ (4:1) containing 50 µL of formic acid, Pd-C (50 mg) was added and the reaction mixture was stirred under hydrogen gas atmosphere for 2 days. The reaction mixture was filtered through Celite® and concentrated in vacuo. The residue was purified using G15 sephadex column with 5 % Ethanol in H_2O to give α:β mixture of disaccharide **1** as a white fluffy powder (10 mg, 64 % yield). ATR-FTIR (cm^{-1}): 3332.41, 2944.46, 2834.21, 1644.79, 1557.72, 1377.99, 1024.81. ^{1}H NMR (600 MHz, Deuterium Oxide) δ 5.21 (d, *J* = 3.2 Hz, 1H), 4.72 (d, *J* = 8.0 Hz, 1H), 4.54 (dd, *J* = 8.4, 5.8 Hz, 2H), 3.97 – 3.94 (m, 4H), 3.93 – 3.88 (m, 4H), 3.86 (d, *J* = 2.0 Hz, 1H), 3.84 – 3.81 (m, 2H), 3.81 – 3.63 (m, 15H), 3.56 – 3.51 (m, 2H), 2.08 (d, *J* = 1.1 Hz, 5H), 2.05 (s, 5H). ^{13}C NMR (151 MHz, D_2O) δ 177.35, 177.04, 104.33, 97.44, 93.02, 82.14, 81.67, 77.93, 77.19, 75.18, 73.29, 72.57, 71.93, 70.21, 63.54, 62.77, 62.65, 58.57, 56.14, 55.18, 24.78, 24.47. ESI-MS: m/z $[M+Na]^+$ cald 447.1585, obsd 447.1750.

Supplemental References:

[1] Tomiya *et al.* (1988) *Analytical Biochemistry* 171:73-90.
[2] Hase *et al.* (1984) *Journal of Biochemistry (Tokyo)* 95:197-203.
[3] Hykollari *et al.* (2017) *Methods in Molecular Biology* 1503:167-184.
[4] Dell *et al.* (1994) *Methods in Enzymology* 230:108-32.
[5] Everest-Dass *et al*. (2013) *Journal of the American Society for Mass Spectrometry*, 24:895-906.
[6] Morchón, *et al.*, 2014, *Veterinary Parasitology* 203:144-152
[7] Thieker *et al.* (2016) *Glycobiology* 26:786-7.
[8] Canales *et al.* (2017) *Angewandte Chemie* 56:14987-91.
[9] Thompson *et al*. (1999) *Structure* 7:169-177.
[10] Jiménez-Castells *et al.* (2016) *Glycobiology* 26:1297-1307.
[11] Yan *et al.* (2013) *Journal of Biological Chemistry* 288:21015-28.
[12] Czernecki and Ayadi (1995) *Canadian Journal of Chemistry* 73:343-350.
[13] Tsai *et al.* (2013) *Chemical Science* 4:468-481.
[14] Feng *et al.* (2004) *Organic and Biomolecular Chemistry* 2:1617-1623.
